# Supplementary material for: Symptoms and signs of lung cancer prior to diagnosis: case–control study using electronic health records from ambulatory care within a large US-based tertiary care centre
Source: BMJ Open. 2023 Apr 20;13(4):e068832. doi: 10.1136/bmjopen-2022-068832 (PMC10124310; doi:10.1136/bmjopen-2022-068832)
Supplement: Supplementary data [file bmjopen-2022-068832supp001.pdf]

Symptoms and signs of lung cancer prior to diagnosis: Comparative study using natural language processing of electronic health records

Appendix 1. Diagnostic codes used to identify cases of lung cancer

ICD 9: 162.2 – 162.9

- 162.2 - Malignant neoplasm of main bronchus
- 162.3 - Malignant neoplasm of upper lobe, bronchus or lung
- 162.4 - Malignant neoplasm of middle lobe, bronchus or lung
- 162.5 - Malignant neoplasm of lower lobe, bronchus or lung
- 162.8 - Malignant neoplasm of other parts of bronchus or lung
- 162.9 - Malignant neoplasm of bronchus and lung, unspecified

ICD 10: C34.0 – C34.9

- C34.0 - Malignant neoplasm of main bronchus
- C34.00 - Malignant neoplasm of unspecified main bronchus
- C34.01 - Malignant neoplasm of right main bronchus
- C34.02 - Malignant neoplasm of left main bronchus
- C34.1 - Malignant neoplasm of upper lobe, bronchus or lung
- C34.10 - Malignant neoplasm of upper lobe, unspecified bronchus or lung
- C34.11 - Malignant neoplasm of upper lobe, right bronchus or lung
- C34.12 - Malignant neoplasm of upper lobe, left bronchus or lung
- C34.2 - Malignant neoplasm of middle lobe, bronchus or lung
- C34.3 - Malignant neoplasm of lower lobe, bronchus or lung
- C34.30 - Malignant neoplasm of lower lobe, unspecified bronchus or lung
- C34.31 - Malignant neoplasm of lower lobe, right bronchus or lung
- C34.32 - Malignant neoplasm of lower lobe, left bronchus or lung
- C34.8 - Malignant neoplasm of overlapping sites of bronchus and lung
- C34.80 - Malignant neoplasm of overlapping sites of unspecified bronchus and lung
- C34.81 - Malignant neoplasm of overlapping sites of right bronchus and lung
- C34.82 - Malignant neoplasm of overlapping sites of left bronchus and lung
- C34.9 - Malignant neoplasm of unspecified part of bronchus or lung
- C34.90 - Malignant neoplasm of unspecified part of unspecified bronchus or lung
- C34.91 - Malignant neoplasm of unspecified part of right bronchus or lung
- C34.92 - Malignant neoplasm of unspecified part of left bronchus or lung

Excluded ICD Diagnostic Codes

- ICD-9: 162.0
- ICD-10: C33

Excluded Histology codes

- Mesothelioma: 9050-9055
- Kaposi Sarcoma: 9140
- Lymphoma/leukemia: M9590-M9992

Appendix 2. Symptoms and signs Identified in peer-reviewed literature previously associated with lung cancer in primary care populations

Symptom or sign	ICD 9 code(s)	ICD10 code(s)	References
Ankle swelling	782.3	R60.9	[1]Ellis (2011)
Back pain	724.1	M54.6	[1]Ellis (2011) [2]Molassiotis (2010)
Bone pain	733.9	M85.80	[3]Gould (2008) [4]Nadpara (2015)
Changes in bowel habits	787.99	R19.4	[5]Corner (2005)
Changes in sleep	780.50	G47.9	[5]Corner (2005)
Chest Pain	786.5<br>786.50<br>786.51<br>786.52<br>786.59	R07.9<br>R07.81	[1]Ellis (2011) [4]Nadpara (2015) [5]Corner (2005) [6]Chowienczyk, Hamilton (2020) [7]Walter (2015) [8]Hamilton (2005) [9]Ades (2014) [10]Redaniel (2015) [11]Tod (2008) [12]Mitchell (2013)
Chest crackles or wheeze	786.7	R09.89	[10]Redaniel (2015)
Cough	786.2<br>491.0	R05	[1]Ellis (2011) [2]Molassiotis (2010) [4]Nadpara (2015) [5]Corner (2005) [6]Chowienczyk, Hamilton (2020) [7]Walter (2015) [9]Ades (2014) [10]Redaniel (2015) [11]Tod (2008) [12]Mitchell (2013) [13]Menon (2019)
Dizziness	780.4	R42	[2]Molassiotis (2010)
Fatigue/tiredness	780.79	R53.81<br>R53.8<br>R53.83<br>R53.1	[1]Ellis (2011) [2]Molassiotis (2010) [5]Corner (2005) [6]Chowienczyk, Hamilton (2020) [7]Walter (2015) [8]Hamilton (2005) [10]Redaniel (2015) [11]Tod (2008) [13]Menon (2019)
Fever	780.6<br>780.60	R50.9	[4]Nadpara (2015)
Finger clubbing	781.5	R68.3	[4]Nadpara (2015) [8]Hamilton (2005) [10]Redaniel (2015)
Headache	784.0	R51	[1]Ellis (2011)
Hemoptysis	786.3<br>786.30<br>786.39	R04.2	[1]Ellis (2011) [4]Nadpara (2015) [5]Corner [6]Chowienczyk, Hamilton (2020) [7]Walter (2015) [8]Hamilton (2005) [10]Redaniel (2015) (2005) [11]Tod (2008) [12]Mitchell (2013) [13]Menon (2019) [14]Hippisley-Cox (2011)

Hoarseness	784.49<br>784.42	R49.8<br>R49.0	[1]Ellis (2011) [2]Molassiotis (2010) [7]Walter (2015) [10]Redaniel (2015) [11]Tod (2008) [12]Mitchell (2013)
Lack of appetite	783	R63.0	[1]Ellis (2011) [2]Molassiotis (2010) [5]Corner (2005) [6]Chowienczyk, Hamilton (2020) [7]Walter (2015) [8]Hamilton (2005) [13]Menon (2019)
Lympadenopathy	785.6	R59.9	[10]Redaniel (2015) [12]Mitchell (2013)
Muscle weakness	728.87	M62.81	[4]Nadpara (2015) [12]Mitchell (2013)
Night sweats	780.8	R61	[3]Gould (2008) [5]Corner (2005)
Shortness of breath	786.05<br>786.0<br>786.9	R06.02<br>R06.00<br>R06.09	[1]Ellis (2011) [2]Molassiotis (2010) [4]Nadpara (2015) [5]Corner (2005) [6]Chowienczyk, Hamilton (2020) [7]Walter (2015) [8]Hamilton (2005) [10]Redaniel (2015) [12]Mitchell (2013) [13]Menon (2019)
Shoulder pain	719.41	M25.511<br>M25.512<br>M25.519	[10]Redaniel (2015) [12]Mitchell (2013)
Weight loss	783.21	R63.4	[1]Ellis (2011) [4]Nadpara (2015) [5]Corner (2005) [6]Chowienczyk, Hamilton (2020) [7]Walter (2015) [8]Hamilton (2005) [10]Redaniel (2015) [11]Tod (2008) [12]Mitchell (2013)
Wheezing and stridor	786.07<br>786.1	R06.2<br>R06.1	[4]Nadpara (2015) [10]Redaniel (2015)

1. Ellis PM, Vandermeer R. Delays in the diagnosis of lung cancer. *J Thorac Dis*. 2011;3(3):183-188. doi:10.3978/j.issn.2072-1439.2011.01.01

2. Molassiotis A, Wilson B, Brunton L, Chandler C. Mapping patients' experiences from initial change in health to cancer diagnosis: a qualitative exploration of patient and system factors mediating this process. *Eur J Cancer Care (Engl)*. 2010;19(1):98-109. doi:10.1111/j.1365-2354.2008.01020.x

3. Gould MK, Ghaus SJ, Olsson JK, Schultz EM. Timeliness of Care in Veterans With Non-small Cell Lung Cancer. *Chest*. 2008;133(5):1167-1173. doi:10.1378/chest.07-2654

4. Nadpara PA, Madhavan SS, Tworek C, Sambamoorthi U, Hendryx M, Almubarak M. Guideline-concordant lung cancer care and associated health outcomes among elderly patients in the United States. *J Geriatr Oncol*. 2015;6(2):101-110. doi:10.1016/j.jgo.2015.01.001

5. Corner J, Hopkinson J, Fitzsimmons D, Barclay S, Muers M. Is late diagnosis of lung cancer inevitable? Interview study of patients' recollections of symptoms before diagnosis. *Thorax*. 2005;60(4):314-319. doi:10.1136/thx.2004.029264

6. Chowienczyk S, Price S, Hamilton W. Changes in the presenting symptoms of lung cancer from 2000–2017: a serial cross-sectional study of observational records in UK primary care. *Br J Gen Pract*. 2020;70(692):e193-e199. doi:10.3399/bjgp20X708137

7. Walter FM, Rubin G, Bankhead C, et al. Symptoms and other factors associated with time to diagnosis and stage of lung cancer: a prospective cohort study. *Br J Cancer*. 2015;112(S1):S6-S13. doi:10.1038/bjc.2015.30

8. Hamilton W. What are the clinical features of lung cancer before the diagnosis is made? A population based case-control study. *Thorax*. 2005;60(12):1059-1065. doi:10.1136/thx.2005.045880

9. Ades AE, Biswas M, Welton NJ, Hamilton W. Symptom lead time distribution in lung cancer: natural history and prospects for early diagnosis. *Int J Epidemiol*. 2014;43(6):1865-1873. doi:10.1093/ije/dyu174

10. Redaniel MT, Martin RM, Ridd MJ, Wade J, Jeffreys M. Diagnostic Intervals and Its Association with Breast, Prostate, Lung and Colorectal Cancer Survival in England: Historical Cohort Study Using the Clinical Practice Research Datalink. Metze K, ed. *PLOS ONE*. 2015;10(5):e0126608. doi:10.1371/journal.pone.0126608

11. Tod AM, Craven J, Allmark P. Diagnostic delay in lung cancer: a qualitative study: Diagnostic delay in lung cancer. *J Adv Nurs*. 2008;61(3):336-343. doi:10.1111/j.1365-2648.2007.04542.x

12. Mitchell ED, Rubin G, Macleod U. Understanding diagnosis of lung cancer in primary care: qualitative synthesis of significant event audit reports. *Br J Gen Pract*. 2013;63(606):e37-e46. doi:10.3399/bjgp13X660760

13. Menon U, Vedsted P, Zalounina Falborg A, et al. Time intervals and routes to diagnosis for lung cancer in 10 jurisdictions: cross-sectional study findings from the International Cancer Benchmarking Partnership (ICBP). *BMJ Open*. 2019;9(11):e025895. doi:10.1136/bmjopen-2018-025895

Appendix 3. Span-based Event Extractor

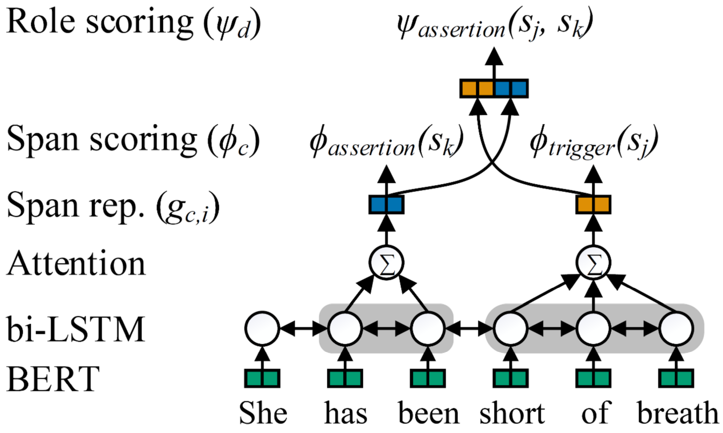

Appendix 4. Comparison of the number of patients with symptoms and signs extracted from the electronic medical record of cases or controls from coded fields versus free-text data using natural language processing (NLP)

Symptom or sign	Identified from NLP (% of patients)	Identified from coded data (% of patients)	Identified from either coded data or NLP (% of patients)	NLP adds (NLP adds n/coded or NLP n)
Cough	1700 (22.6%)	1139 (15.1%)	2227 (29.5%)	1088 (48.9%)
Shortness of breath	1580 (21.0%)	1111 (14.7%)	2128 (28.2%)	1017 (47.8%)
Chest Pain	1241 (16.5%)	981 (13.0%)	1804 (23.9%)	823 (45.6%)
Fatigue	1489 (19.8%)	959 (12.7%)	2063 (27.4%)	1104 (53.5%)
Shoulder pain	513 (6.8%)	594 (7.9%)	893 (11.9%)	299 (33.5%)
Dizziness	1331 (17.7%)	536 (7.1%)	1618 (21.5%)	1082 (66.9%)
Ankle swelling	2081 (27.6%)	509 (6.8%)	2285 (30.3%)	1776 (77.7%)
Headache	1281 (17.0%)	415 (5.5%)	1509 (20.0%)	1094 (72.5%)
Weight loss	646 (8.6%)	328 (4.4%)	830 (11.0%)	502 (60.5%)
Fever	1517 (20.1%)	252 (3.3%)	1656 (22.0%)	1404 (84.8%)
Chest crackles or wheeze	834 (11.1%)	242 (3.2%)	972 (12.9%)	730 (75.1%)
Lympadenopathy	52 (0.7%)	223 (3.0%)	256 (3.4%)	33 (12.9%)
Bone pain	829 (11.0%)	216 (2.9%)	995 (13.2%)	779 (78.3%)
Muscle weakness	1327 (17.6%)	205 (2.7%)	1436 (19.1%)	1231 (85.7%)
Back pain	1220 (16.2%)	154 (2.0%)	1296 (17.2%)	1142 (88.1%)
Changes in sleep	662 (8.8%)	137 (1.8%)	765 (10.2%)	628 (82.1%)
Hoarseness	130 (1.7%)	118 (1.6%)	200 (2.7%)	82 (41.0%)
Hemoptysis	133 (1.8%)	94 (1.3%)	182 (2.4%)	88 (48.4%)
Night sweats	480 (6.4%)	72 (1.0%)	521 (6.9%)	449 (86.2%)
Lack of appetite	626 (8.3%)	59 (0.8%)	653 (8.7%)	594 (91.0%)
Change in bowel habits	1465 (19.4%)	59 (0.8%)	1491 (19.8%)	1432 (96.0%)
Finger clubbing	41 (0.5%)	1 (0.0%)	41 (0.5%)	40 (97.6%)

Appendix 5. Multivariable analysis of symptoms or signs of cases compared to controls at 1, 3, 6 and 12 months prior to diagnosis/index date

Symptom or sign	12 months OR	6 months OR	3 months OR	1 month OR	At diagnosis OR
**Finger clubbing**	>1,000 (0.0 - >1,000)	>1,000 (0.0 - >1,000)	>1,000 (0.0 - >1,000)	60.7 (10.6 - 348.7)***	50.1 (8.9 - 283.3)***
**Lymphadenopathy**	0.7 (0.3 - 1.4)	1.3 (0.7 - 2.4)	1.3 (0.8 - 2.3)	1.7 (1.0 - 2.8)*	5.8 (3.8 - 8.8)***
**Cough**	1.9 (1.5 - 2.4)***	3.1 (2.4 - 4.0)***	4.0 (3.1 - 5.2)***	5.0 (3.8 - 6.5)***	4.7 (3.5 - 6.3)***
**Hemoptysis**	2.1 (1.0 - 4.4)*	3.2 (1.9 - 5.3)***	3.1 (1.9 - 4.9)***	3.4 (2.2 - 5.4)***	3.5 (2.2 - 5.5)***
**Chest crackles or wheeze**	2.5 (1.9 - 3.5)***	3.1 (2.3 - 4.1)***	3.0 (2.3 - 4.0)***	3.0 (2.3 - 4.0)***	3.2 (2.4 - 4.3)***
**Weight loss**	1.2 (0.9 - 1.8)	2.1 (1.5 - 2.8)***	2.6 (1.9 - 3.4)***	2.8 (2.1 - 3.7)***	2.9 (2.2 - 3.9)***
**Back pain**	2.8 (2.1 - 3.6)***	2.5 (1.9 - 3.2)***	2.5 (1.9 - 3.2)***	2.4 (1.9 - 3.1)***	2.4 (1.8 - 3.1)***
**Bone pain**	2.8 (2.1 - 3.7)***	2.7 (2.1 - 3.6)***	2.4 (1.8 - 3.2)***	2.3 (1.7 - 3.0)***	2.3 (1.7 - 3.0)***
**Shortness of breath**	0.7 (0.5 - 1.0)*	1.0 (0.7 - 1.3)	1.3 (1.0 - 1.7)	1.6 (1.2 - 2.1)**	1.9 (1.4 - 2.5)***
**Fatigue**	1.6 (1.2 - 2.1)***	1.6 (1.3 - 2.1)***	1.9 (1.4 - 2.5)***	1.8 (1.4 - 2.4)***	1.8 (1.3 - 2.3)***
**Chest Pain**	1.1 (0.8 - 1.4)	1.2 (0.9 - 1.5)	1.2 (1.0 - 1.6)	1.3 (1.0 - 1.6)	1.4 (1.1 - 1.8)*
**Shoulder pain**	1.3 (0.9 - 1.7)	1.4 (1.0 - 1.8)*	1.3 (1.0 - 1.7)	1.3 (1.0 - 1.7)	1.3 (0.9 - 1.7)
**Ankle swelling**	1.5 (1.1 - 1.9)**	1.3 (1.0 - 1.7)	1.3 (1.0 - 1.7)	1.3 (1.0 - 1.7)	1.1 (0.9 - 1.5)
**Headache**	1.0 (0.7 - 1.3)	1.1 (0.8 - 1.4)	1.0 (0.8 - 1.3)	1.0 (0.8 - 1.3)	1.1 (0.8 - 1.4)
**Hoarseness**	0.9 (0.5 - 1.7)	1.1 (0.7 - 1.8)	1.0 (0.6 - 1.6)	1.1 (0.7 - 1.7)	1.0 (0.7 - 1.7)
**Changes in bowel habits**	1.2 (0.9 - 1.6)	1.0 (0.8 - 1.4)	1.1 (0.8 - 1.5)	1.0 (0.8 - 1.4)	1.0 (0.8 - 1.4)
**Muscle weakness**	1.0 (0.7 - 1.3)	0.9 (0.7 - 1.2)	1.0 (0.7 - 1.3)	1.0 (0.8 - 1.3)	1.0 (0.7 - 1.3)
**Night sweats**	0.9 (0.6 - 1.4)	0.9 (0.7 - 1.4)	0.9 (0.7 - 1.3)	0.9 (0.6 - 1.3)	0.8 (0.6 - 1.2)
**Lack of appetite**	0.5 (0.3 - 0.7)***	0.6 (0.4 - 0.8)**	0.6 (0.4 - 0.8)**	0.6 (0.4 - 0.9)**	0.7 (0.5 - 0.9)*
**Dizziness**	0.8 (0.6 - 1.0)	0.7 (0.5 - 0.9)**	0.7 (0.5 - 0.9)**	0.6 (0.5 - 0.8)**	0.6 (0.4 - 0.8)***
**Changes in sleep**	0.8 (0.5 - 1.1)	0.5 (0.4 - 0.7)***	0.4 (0.3 - 0.6)***	0.4 (0.3 - 0.6)***	0.4 (0.3 - 0.6)***
**Fever**	0.6 (0.4 - 0.8)***	0.5 (0.4 - 0.7)***	0.5 (0.4 - 0.6)***	0.5 (0.3 - 0.6)***	0.4 (0.3 - 0.6)***

Note: Models adjusted for comorbidities using van Walraven weighted score. Confidence intervals for significant ORs do not incorporate 1.0 due to rounding.

***** p<0.05

****** p<0.01

******* p<0.001

Appendix 6. Frequency of symptoms and signs in cases and controls with and without chronic respiratory disease

Symptom or sign	Chronic respiratory disease		No chronic respiratory disease	
	Control (n=1252)	Case (n=353)	Control (n=5589)	Case (n=345)
Cough	636 (50.8%)	312 (88.4%)	1018 (18.2%)	261 (75.7%)
Shortness of breath	623 (49.8%)	307 (87.0%)	990 (17.7%)	208 (60.3%)
Fatigue	459 (36.7%)	266 (75.4%)	1128 (20.2%)	210 (60.9%)
Ankle swelling	516 (41.2%)	250 (70.8%)	1322 (23.7%)	197 (57.1%)
Chest Pain	439 (35.1%)	228 (64.6%)	962 (17.2%)	175 (50.7%)
Chest crackles or wheeze	307 (24.5%)	268 (75.9%)	268 (4.8%)	129 (37.4%)
Back pain	278 (22.2%)	191 (54.1%)	668 (12.0%)	159 (46.1%)
Changes in bowel habits	337 (26.9%)	195 (55.2%)	818 (14.6%)	141 (40.9%)
Muscle weakness	327 (26.1%)	177 (50.1%)	775 (13.9%)	157 (45.5%)
Fever	433 (34.6%)	177 (50.1%)	901 (16.1%)	145 (42.0%)
Weight loss	165 (13.2%)	191 (54.1%)	357 (6.4%)	117 (33.9%)
Headache	324 (25.9%)	175 (49.6%)	881 (15.8%)	129 (37.4%)
Dizziness	366 (29.2%)	174 (49.3%)	953 (17.1%)	125 (36.2%)
Bone pain	207 (16.5%)	141 (39.9%)	518 (9.3%)	129 (37.4%)
Lack of appetite	142 (11.3%)	116 (32.9%)	315 (5.6%)	80 (23.2%)
Shoulder pain	200 (16.0%)	92 (26.1%)	513 (9.2%)	88 (25.5%)
Lymphadenopathy	35 (2.8%)	79 (22.4%)	70 (1.3%)	72 (20.9%)
Night sweats	113 (9.0%)	89 (25.2%)	258 (4.6%)	61 (17.7%)
Changes in sleep	178 (14.2%)	90 (25.5%)	453 (8.1%)	44 (12.8%)
Hemoptysis	31 (2.5%)	72 (20.4%)	36 (0.6%)	43 (12.5%)
Hoarseness	55 (4.4%)	45 (12.7%)	78 (1.4%)	22 (6.4%)
Finger clubbing	1 (0.1%)	28 (7.9%)	1 (0.0%)	11 (3.2%)

Appendix 7. Multivariate analysis of symptoms and signs in patients with and without chronic respiratory disease

Symptom or sign	Chronic respiratory disease			No chronic respiratory disease		
	Univariate Odds ratio (95%CI)	Multivariate Odds ratio (95%CI)	Multivariate P value	Univariate Odds ratio (95%CI)	Multivariate Odds ratio (95%CI)	Multivariate P value
Finger clubbing	47.3 (6.1 - 364.5)	17.8 (1.3 - 247.1)	0.0322	>1,000 (0.0 - >1,000)	267.7 (0.1 - >1,000)	0.1783
Chest crackles or wheeze	9.4 (6.3 - 14.2)*	4.9 (2.6 - 9.0)	<0.0001	9.8 (7.0 - 13.9)*	3.2 (2.0 - 5.2)	<0.0001
Hemoptysis	12.5 (6.2 - 25.3)*	4.4 (1.7 - 11.5)	0.0028	20.3 (10.2 - 40.5)*	3.8 (1.5 - 9.8)	0.0049
Weight loss	7.1 (4.7 - 10.5)*	4.0 (2.2 - 7.4)	<0.0001	3.8 (2.8 - 5.3)*	1.6 (1.0 - 2.5)	0.0643
Lympadenopathy	7.1 (3.9 - 13.0)*	3.3 (1.3 - 7.9)	0.0089	12.0 (7.2 - 19.9)*	8.5 (4.3 - 17.0)	<0.0001
Fatigue	5.2 (3.6 - 7.6)*	2.9 (1.6 - 5.5)	0.0008	4.2 (3.2 - 5.6)*	1.7 (1.1 - 2.6)	0.0128
Back pain	4.6 (3.2 - 6.6)*	2.4 (1.4 - 4.1)	0.0014	4.8 (3.6 - 6.4)*	2.1 (1.4 - 3.2)	0.0003
Cough	6.5 (4.2 - 10.2)*	2.2 (1.1 - 4.3)	0.0189	12.2 (9.0 - 16.6)*	6.3 (4.2 - 9.3)	<0.0001
Bone pain	3.8 (2.6 - 5.5)*	2.1 (1.1 - 4.0)	0.0168	5.3 (3.9 - 7.2)*	2.5 (1.6 - 3.9)	0.0001
Shortness of breath	6.5 (4.1 - 10.3)*	1.6 (0.8 - 3.2)	0.1688	5.1 (3.9 - 6.7)*	1.9 (1.3 - 2.9)	0.0024
Changes in bowel habits	2.7 (2.0 - 3.8)*	1.3 (0.7 - 2.3)	0.4474	2.5 (1.9 - 3.4)*	0.9 (0.6 - 1.4)	0.7286
Night sweats	3.1 (2.1 - 4.7)*	1.2 (0.6 - 2.4)	0.5393	3.8 (2.6 - 5.7)*	0.9 (0.5 - 1.7)	0.8542
Ankle swelling	2.8 (2.0 - 3.9)*	1.1 (0.6 - 2.0)	0.6696	3.1 (2.4 - 4.0)*	1.2 (0.8 - 1.8)	0.3121
Shoulder pain	1.6 (1.1 - 2.4)	1.1 (0.6 - 2.0)	0.7589	2.9 (2.1 - 4.0)*	1.6 (1.0 - 2.5)	0.0484
Hoarseness	2.5 (1.4 - 4.4)	1.0 (0.5 - 2.3)	0.9617	4.1 (2.2 - 7.7)*	0.9 (0.4 - 2.2)	0.8729
Headache	2.5 (1.9 - 3.5)*	0.9 (0.5 - 1.7)	0.8551	2.2 (1.7 - 2.9)*	1.0 (0.7 - 1.6)	0.8319
Chest Pain	2.6 (1.9 - 3.6)*	0.9 (0.5 - 1.6)	0.7953	3.7 (2.8 - 4.8)*	1.5 (1.0 - 2.2)	0.0494
Muscle weakness	2.3 (1.7 - 3.2)*	0.9 (0.5 - 1.7)	0.7901	3.1 (2.3 - 4.1)*	1.1 (0.7 - 1.7)	0.6809
Dizziness	2.3 (1.7 - 3.3)*	0.9 (0.5 - 1.6)	0.7450	1.8 (1.3 - 2.4)*	0.5 (0.3 - 0.8)	0.0027
Lack of appetite	2.6 (1.8 - 3.8)*	0.5 (0.3 - 1.0)	0.0667	1.8 (1.3 - 2.6)	0.5 (0.3 - 0.9)	0.0122
Changes in sleep	1.6 (1.1 - 2.3)	0.5 (0.3 - 0.9)	0.0233	1.1 (0.7 - 1.6)	0.3 (0.2 - 0.6)	0.0004
Fever	1.6 (1.2 - 2.2)	0.3 (0.2 - 0.6)	0.0003	2.5 (1.9 - 3.3)*	0.6 (0.4 - 0.9)	0.0229

Note: Models adjusted for comorbidities using van Walraven weighted score

*Significant at p<0.0001
