# Supplementary material for: Symptoms and signs of lung cancer prior to diagnosis: case–control study using electronic health records from ambulatory care within a large US-based tertiary care centre
Source: BMJ Open. 2023 Apr 20;13(4):e068832. doi: 10.1136/bmjopen-2022-068832 (PMC10124310; doi:10.1136/bmjopen-2022-068832)
Supplement: Author's manuscript [file bmjopen-2022-068832.draft_revisions.pdf]

BMJ Open

BMJ Open is committed to open peer review. As part of this commitment we make the peer review history of every article we publish publicly available.

When an article is published we post the peer reviewers' comments and the authors' responses online. We also post the versions of the paper that were used during peer review. These are the versions that the peer review comments apply to.

The versions of the paper that follow are the versions that were submitted during the peer review process. They are not the versions of record or the final published versions. They should not be cited or distributed as the published version of this manuscript.

BMJ Open is an open access journal and the full, final, typeset and author-corrected version of record of the manuscript is available on our site with no access controls, subscription charges or pay-per-view fees (http://bmjopen.bmj.com).

If you have any questions on BMJ Open's open peer review process please email info.bmjopen@bmj.com

BMJ Open

Symptoms and signs of lung cancer prior to diagnosis: Comparative study using electronic health records

Journal:	*BMJ Open*
Manuscript ID	bmjopen-2022-068832
Article Type:	Original research
Date Submitted by the Author:	01-Oct-2022
Complete List of Authors:	Prado, Maria G.; University of Washington, Department of Family Medicine<br>Kessler, Larry ; University of Washington, Health Services<br>Au, Margaret A; University of Washington, Department of Family Medicine<br>Burkhardt, Hannah; University of Washington, Department of Biomedical Informatics and Medical Education<br>Zigman Suchsland, Monica; University of Washington<br>Kowalski, Lesleigh; University of Washington, Department of Family Medicine<br>Stephens, KA; University of Washington,<br>Yetisgen, Meliha; University of Washington, Department of Biomedical Informatics and Medical Education<br>Walter, Fiona M; Queen Mary University of London; University of Cambridge, Department of Public Health and Primary Care<br>Neal, Richard; University of Exeter<br>Lybarger, Kevin; University of Washington, Department of Biomedical Informatics and Medical Education<br>Thompson, Caroline; The University of North Carolina at Chapel Hill, Department of Epidemiology; San Diego State University<br>Al Achkar, Morhaf; University of Washington, Family Medicine<br>Sarma, Elizabeth; NIH, National Cancer Institute<br>Turner, Grace; University of Washington, Department of Biomedical Informatics and Medical Education<br>Farjah, Farhood ; University of Washington<br>Thompson, Matthew; University of Washington, Department of Family Medicine
Keywords:	Health informatics < BIOTECHNOLOGY & BIOINFORMATICS, Thoracic medicine < INTERNAL MEDICINE, Respiratory tract tumours < ONCOLOGY, PRIMARY CARE

SCHOLARONE™ Manuscripts

I, the Submitting Author has the right to grant and does grant on behalf of all authors of the Work (as defined in the below author licence), an exclusive licence and/or a non-exclusive licence for contributions from authors who are: i) UK Crown employees; ii) where BMJ has agreed a CC-BY licence shall apply, and/or iii) in accordance with the terms applicable for US Federal Government officers or employees acting as part of their official duties; on a worldwide, perpetual, irrevocable, royalty-free basis to BMJ Publishing Group Ltd ("BMJ") its licensees and where the relevant Journal is co-owned by BMJ to the co-owners of the Journal, to publish the Work in this journal and any other BMJ products and to exploit all rights, as set out in our licence.

The Submitting Author accepts and understands that any supply made under these terms is made by BMJ to the Submitting Author unless you are acting as an employee on behalf of your employer or a postgraduate student of an affiliated institution which is paying any applicable article publishing charge ("APC") for Open Access articles. Where the Submitting Author wishes to make the Work available on an Open Access basis (and intends to pay the relevant APC), the terms of reuse of such Open Access shall be governed by a Creative Commons licence – details of these licences and which Creative Commons licence will apply to this Work are set out in our licence referred to above.

Other than as permitted in any relevant BMJ Author's Self Archiving Policies, I confirm this Work has not been accepted for publication elsewhere, is not being considered for publication elsewhere and does not duplicate material already published. I confirm all authors consent to publication of this Work and authorise the granting of this licence.

For peer review only

Symptoms and signs of lung cancer prior to diagnosis: Case-control study using electronic health records

Maria G. Prado 1

Larry G. Kessler 3

Margaret A Au 1

Hannah Burkhardt 2

Monica Zigman Suchsland 1

Lesleigh Kowalski 1

Kari A. Stephens 1

Meliha Yetisgen 2

Fiona M. Walter 5,6

Richard D Neal 7

Kevin Lybarger 2

Caroline Thompson 8, 9

Morhaf Al Achkar 1

Elizabeth A. Sarma 10

Grace Turner 2

Farhood Farjah 4

Matthew Thompson 1

Affiliations

1 Department of Family Medicine, University of Washington, Seattle, WA, USA

2 Department of Biomedical Informatics and Medical Education, University of Washington, Seattle, WA, USA

For peer review only

For peer review only - http://bmjopen.bmj.com/site/about/guidelines.xhtml

3 Department of Health Systems and Population Health, School of Public Health, University of Washington, Seattle, WA, USA

4 Department of Surgery, University of Washington, Seattle, WA, USA

5 Wolfson Institute of Population Health, Barts and The London School of Medicine and Dentistry, Queen Mary University of London, UK

6 The Primary Care Unit, Department of Public Health and Primary Care, University of Cambridge, UK

7 University of Exeter Medical School, University of Exeter, Exeter

8 Department of Epidemiology, Gillings School of Global Public Health, The University of North Carolina at Chapel Hill, Chapel Hill, NC

9 Division of Epidemiology and Biostatistics, School of Public Health, San Diego State University, San Diego, CA

10 Healthcare Delivery Research Program, Division of Cancer Control and Population Sciences, National Cancer Institute, Bethesda, Maryland

Corresponding author: Matthew Thompson.

mjt@uw.edu

University of Washington, Box 354696

4225 Roosevelt NE, Suite 308, Seattle, WA 98105

Tel #: (206) 616-8149

For peer review only

Abstract

Objective: Lung cancer is the most common cause of cancer-related death in the United States (US). While most patients are diagnosed following symptomatic presentation, no studies have compared symptoms and physical examination signs at or prior to diagnosis from electronic health records (EHR) in the US. We aimed to identify symptoms and signs in patients prior to diagnosis in EHR data.

Design: Case-control study

Setting: Ambulatory care clinics at a large tertiary care academic health center in the US

Participants, Outcomes: We studied 698 primary lung cancer cases in adults diagnosed between January 1, 2012 and December 31, 2019, and 6,841 controls matched by age, sex, smoking status, and type of clinic. Coded and free-text data from the EHR were extracted from 2 years prior to diagnosis date for cases and index date for controls. Univariate and multivariate conditional logistic regression were used to identify symptoms and signs associated with lung cancer at time of diagnosis, and 1, 3, 6, and 12 months before the diagnosis/index dates.

Results: Eleven symptoms and signs recorded during the study period were associated with a significantly higher chance of being a lung cancer case in multivariate analyses. Of these, seven were significantly associated with lung cancer six months prior to diagnosis: hemoptysis (OR 3.2, 95%CI 1.9-5.3), cough (OR 3.1, 95%CI 2.4-4.0), chest crackles or wheeze (OR 3.1, 95%CI 2.3-4.1), bone pain (OR 2.7, 95%CI 2.1-3.6), back pain (OR 2.5, 95%CI 1.9-3.2), weight loss (OR 2.1, 95%CI 1.5-2.8) and fatigue (OR 1.6, 95%CI 1.3-2.1).

Conclusions: Patients diagnosed with lung cancer appear to have symptoms and signs recorded in the EHR that distinguish them from similar matched patients in ambulatory care, often six months or more before diagnosis. These findings suggest opportunities to improve the diagnostic process for lung cancer.

For peer review only - http://bmjopen.bmj.com/site/about/guidelines.xhtml

Strengths and limitations of this study

Strengths

- First case-control study in the US to use routine, prospectively collected EHR data to describe the frequency of symptoms and signs of lung cancer and estimate associations with incident lung cancer cases compared to non-lung cancer patients in ambulatory care.
- Using Natural Language Processing (NLP) techniques to extract symptoms and signs from unstructured data provides a more complete dataset of clinical features presence compared to using coded data alone.
- Case control design recruited cases from ambulatory care population, and controls were randomly selected in a 10:1 ratio based on case clinic type, to reduce the possibility of bias
- Symptoms and signs differentiated patients with lung cancer at least six months prior to diagnosis, suggesting opportunities to improve early detection.

Limitations

- Single center study based at ambulatory care clinics associated with a large academic medical center.
- Criteria for selection of cases and controls differed slightly; Cases were selected based on a date of the first lung cancer diagnostic code in the EHR, whereas controls were selected based on having a visit to the matched type of clinic type within 3 months of the case diagnosis date
- EHR data could be subject to misclassification for characteristics such as smoking status or comorbidity, but we attempted to control for these.
- Availability and timing of symptom data for cases and controls is based on number and frequency of patient interactions with the healthcare system which could be due to a range of factors.

For peer review only - http://bmjopen.bmj.com/site/about/guidelines.xhtml

Introduction

Lung cancer is the third most common cancer and the leading cause of cancer death in the United States (US).[1] Most patients with lung cancer are diagnosed following presentation to healthcare settings with symptoms or diagnosed incidentally, and many patients (47%) present with late-stage disease (stages 3 or 4).[2] Screening for lung cancer remains low in the US.[3,4] In addition to optimizing screening, early detection efforts have focused on recognition of lung cancer symptoms with an overall goal of identifying patients at earlier, more treatable stages of the disease.[5–7] These symptoms range from 'alarm' symptoms, such as hemoptysis (a rare symptom), to relatively non-specific symptoms, such as persistent cough or unexpected weight loss.[6]

Diagnosing lung cancer based on non-specific symptom presentation is challenging, as these symptoms are more commonly associated with benign conditions or may be overlooked for long periods of time. A study of over 43 million patients using Medicare claims data identified a median time from symptom onset to diagnosis of approximately six months.[8] However, claims data lack the granularity needed to identify which clinical features patients present and how these might be used to differentiate patients with lung cancer from the vast majority of patients with benign conditions. To fill this gap, we examined the frequency and association of symptoms and physical examination signs in patients in ambulatory care prior to lung cancer diagnosis and matched controls.

Methods

Study design

We performed a case-control study using data from the University of Washington Medicine (UWM) electronic health records (EHR) and the Seattle/Puget Sound Surveillance, Epidemiology, and End Results (SEER) Program, a National Cancer Institute-supported national cancer registry.[9] This study was approved by the University of Washington Human Subjects Division (STUDY 000013191). A patient and caregiver stakeholder group was involved over a period of 2 years involving regular meetings in the design of this study and in the interpretation of the findings.

For peer review only

For peer review only - http://bmjopen.bmj.com/site/about/guidelines.xhtml

Setting

Cases and controls were identified from patients who received ambulatory care at UWM, a large tertiary care academic health center.

Participants

Cases were identified from UWM patients aged 18 years or older, with a first primary lung cancer diagnosis (see International Classification of Diseases (ICD) 9 and 10 codes in Appendix 1) between January 1, 2012 and December 31, 2019, who had an established relationship with a UWM ambulatory care setting in the 2 years before the date of their first recorded lung cancer ICD code in the EHR (EHR diagnosis date). We chose the above study period because of the limited quality of the UWM EHR data prior to 2012. We defined ambulatory care as at least one encounter in family medicine, internal medicine, women's health, obstetrics and gynecology, urgent care, and/or emergency medicine. We used linkage to the regional SEER registry to verify cancer incident cases. Cases were excluded if they did not match with the SEER registry or had evidence of a history of any of the following cancers identified using histology codes in SEER: tracheal cancer, mesothelioma, Kaposi sarcoma, lymphoma, or leukemia. Controls were identified from UWM patients with at least one encounter with the same type of ambulatory clinic within 3 months of the EHR diagnosis date of the index case (matching date). For each case, 10 controls were individually matched to the index case by age, sex (male, female), smoking status (ever vs. never), and type of ambulatory care clinic where lung cancer case presented (emergency medicine vs other clinics listed above). We chose a 10:1 control: case match because we recognize the wide variety of patients presenting to ambulatory care settings. Controls were excluded if they had any lung cancer ICD codes in their EHR prior to their matched case diagnosis (index) date. Excluded cancers in cases (based on histology codes from the SEER registry) were not identified in controls as registry data was not available for controls. We also excluded any cases and controls who did not have any ICD codes in any encounter in the 2 years prior to diagnosis date (cases) or index date (controls) to ensure availability of data on pre-diagnosis symptoms and signs.

For peer review only - http://bmjopen.bmj.com/site/about/guidelines.xhtml

Data Collection

The UWM enterprise-wide data warehouse (EDW) was used to obtain data; this provides a central repository that integrates EHR across the UWM health care system including ambulatory care, specialty care and hospital services. Cases were identified during the study period using ICD codes (Appendix 1) and were linked to SEER to ensure accuracy of case identification and obtain history of previous cancers, histology (for exclusions and lung cancer type), and stage at diagnosis. The date of diagnosis was determined by date of pathology report at UWM. For cases that did not have a diagnosis through pathology or had a discrepancy greater than 30 days between date of pathology and first recorded lung cancer ICD code, two of three clinicians (MT, LKF, MAlA) reviewed the EHR of these cases to adjudicate dates. Controls were randomly sampled from within the matching strata, based on this adjudicated date of diagnosis.

Cases who had undergone lung cancer screening using low-dose computed tomography (LDCT) within the 12 months prior to diagnosis date were identified from billing code (Current Procedural Terminology or CPT 71271) and/or ICD codes (V76.0 [ICD-9] or Z12.2 [ICD-10].

An EHR data extraction protocol was applied to all encounters in the 2-year period prior and up to six months following the diagnosis date (cases) and index date (controls). These data comprised of demographics (e.g., age, sex, race, ethnicity), all ICD codes and CPT procedure codes linked to encounters such as laboratory tests, imaging procedures, and pathology data. We also extracted corresponding unstructured clinical notes for any of the above encounters. ICD codes recorded during the 2-year period prior to diagnosis for cases or prior to index date for controls were searched for the presence of 31 potential comorbidities to calculate the Elixhauser comorbidity index.[10] We excluded lung cancer ICD code information from this calculation. These index scores were then used to calculate van Walraven weighted scores for each patient, a range of -19 to 89.[11,12]

Symptoms and signs

For peer review only - http://bmjopen.bmj.com/site/about/guidelines.xhtml

We identified symptoms and signs using coded data and unstructured data. A list of symptoms and signs which have previously been reported in cohort or case-control studies of individuals with lung cancer were identified from systematic reviews, hand review of individual studies, and from contact with experts in oncology, cardiothoracic surgery, and primary care (FW, RN, FF, MT, see Appendix 2).[5,6,13–18] These were mapped to ICD codes, and used to search the extracted EHR coded data for any encounters that included any of these ICD codes in the 2-year observation period.

Symptoms and signs were automatically extracted from free-text clinical notes using natural language processing (NLP), including notes for all visit types in the 2-year period. In previous work, we developed a deep learning symptom extraction model using the COVID-19 Annotated Clinical Text Corpus (CACT),[19] which was then adapted to the lung cancer domain. This involved creating the Lung Cancer Annotated Clinical Text (LACT) Corpus, composed of 270 notes from lung cancer patients (170 training and 100 test notes).[20] We trained the lung cancer symptom extractor by combining the CACT and LACT training sets. On the LACT test set, the lung cancer symptom extractor achieved 0.72 F1 for symptom identification and 0.65 F1 for assertion prediction. This extraction performance is comparable to the LACT inter-rater agreement of 0.82 F1 for symptom identification and 0.79 F1 for assertion prediction, indicating the model is achieving approximately human-level performance. We included the extracted symptoms and signs with assertion value present.

Data analysis

Frequencies and counts were calculated for characteristics of cases and controls. The number of symptoms and signs obtained from coded data was compared to that obtained from free-text data using descriptive statistics. The proportion of patients with evidence of each symptom/sign occurring in the 2-year period prior to the diagnosis or index date was described for cases and controls. Odds of patients' case status, based on symptoms and signs identified from a combined dataset of coded and free-text data, were estimated using unadjusted conditional logistic regression. Symptoms and signs associated with lung cancer in unadjusted regressions ($p < 0.1$) were included into multivariate conditional logistic regression analyses.

For peer review only

We used the van Walraven comorbidity score to adjust for population differences in comorbidity burden. Analyses were repeated excluding symptom and sign data from 1, 3, 6, and 12 months before the diagnosis (or index) date. Lag times were chosen to provide information on the pattern of symptom-related visits over time and identify the symptoms and signs presenting furthest from diagnosis. We conducted secondary analyses investigating the potential effect of chronic respiratory disease (CRD) status, as defined by the presence of ICD codes within the Elixhauser chronic respiratory disease subgroup, on presence of symptoms and signs in the pre-diagnostic interval. We expected patients with CRD to present with symptoms and signs similar to those that present in early lung cancer. We assessed the effect of CRD by repeating the conditional logistic regression model including CRD as a covariate.

Statistical analyses were conducted using Python 3.7 with the packages SciPy (version 1.4.1) and Statsmodels (version 0.11.1). The study was reported in line with the STROBE guidelines.[21]

Results

Participants

Selection of cases & controls

A total of 7,883 patients with lung cancer ICD codes were identified in the UWM EDW over the study period. Following linkage of these patients and those identified as having a primary lung tumor from SEER, 4,115 patients were identified common to both, including 741 cases. After matching 7,410 controls, a chart review resulted in exclusion of 43 additional cases. Controls that were matched to these 43 cases were excluded (n = 422), resulting in 698 cases matched to 6,841 controls.

Description of cases and controls

Cases and controls were similar in terms of sex and race (cases 50.6% male, 75.5% White; controls 50.5% male, 75.7% White, see Table 1). Cases had higher comorbidity scores (M = 14.9, SD = 11.6) than controls (M = 4.4, SD = 8.6). Cases also had a greater median number of health care visits over the 2-year period prior to diagnosis (51.0, 95%CI: 28.0-97.8) than controls (23.0,

For peer review only - http://bmjopen.bmj.com/site/about/guidelines.xhtml

95%CI: 9.0-53.0). The difference in median number of health care visits was greater in the last 3-month period prior to the diagnosis/index date (cases 21.0, 95%CI: 12.0-35.0 vs. controls 5.0, 95%CI: 2.0-11.0) than in the 2nd, 3rd, or 4th quarters prior to diagnosis. The stage distribution of cases was as follows: Stage 1- 29%, Stage 2- 7%, Stage 3- 17%, and Stage 4 -42% (5% were Stage 0 or Unknown Stage).

Frequency of symptoms and signs extracted from coded and free-text data

Of the 22 symptoms and signs that we systematically examined, NLP identified 20 of the 22 symptoms and signs in greater proportions of patients affected than from the coded data alone (see Appendix 3). In comparison to coded data, we saw a range of 12.9% to 97.6% greater symptom and signs reports with NLP of textual clinical notes. In contrast, a greater proportion of patients had two symptoms and signs (shoulder pain, lymphadenopathy) identified from coded rather than free-text data.

Comparison of frequency of symptoms and signs between cases and controls

The frequency of all 22 symptoms and signs examined was higher in cases than controls (see Table 2). Moreover, the ranking of symptoms and signs differed slightly between cases and controls, with cases reporting cough (82.1%), shortness of breath (73.8%), fatigue (68.2%), ankle swelling (64.0%), and chest pain (57.7%), whereas controls reported ankle swelling (26.9%), cough (24.2%), shortness of breath (23.6%), fatigue (23.2%) and chest pain (20.5%) most frequently. Hemoptysis occurred relatively infrequently among cases (16.5%) and rarely among controls (1.0%).

Univariate associations of symptoms and signs between cases and controls

In models adjusted for comorbidity score, when considered independently, all 22 symptoms and signs had odds ratios that were significantly different between cases and controls (all $p < 0.0001$, see Table 3). The symptoms and signs with the largest odds ratios (OR) significantly associated with a higher chance of being a case were finger clubbing (OR 175.7, 95%CI: 40.1-

For peer review only

770.0), hemoptysis (OR 14.5, 95%CI: 10.2-20.8), cough (OR 11.1, 95%CI: 8.8-13.9), chest crackles or wheeze (OR 9.9, 95%CI: 8.1-12.2), and lympadenopathy (OR 9.4, 95%CI: 6.9-12.8).

Multivariable associations of symptoms and signs between cases and controls

We included all 22 symptoms and signs from the univariate analysis and comorbidity score in a multivariate analysis. After mutual adjustment, 15 had significant ORs (all $p < 0.05$, see Table 3). The presence of 11 symptoms and signs were associated with a significantly higher odds of being a case, with ORs ranging from 1.4 (chest pain) to 50.1 (finger clubbing). The largest ORs were noted for finger clubbing (OR 50.1, 95%CI: 8.9-283.3), lymphadenopathy (OR 5.8, 95%CI: 3.8-8.8), cough (OR 4.7, 95%CI: 3.5-6.3), hemoptysis (OR 3.5, 95%CI: 2.2-5.5) and chest crackles or wheeze (OR 3.2, 95%CI: 2.4-4.3). In contrast, the presence of four symptoms was associated with a significantly higher odds of being a control: fever (OR 0.4, 95%CI: 0.3-0.6), changes in sleep (OR 0.5, 95%CI: 0.3-0.6), dizziness (OR 0.6, 95%CI: 0.4-0.8), and lack of appetite (OR 0.7, 95%CI: 0.5-0.9).

We repeated the multivariate analysis, excluding symptoms and signs recorded in periods of 1, 3, 6 and 12 months prior to diagnosis (see Figure 2). Some symptoms and signs remained significantly associated with cases up to 6 months prior to diagnosis (cough, hemoptysis, chest crackles and wheeze, weight loss, back pain, bone pain, fatigue). Of these, all except weight loss were also significantly associated with cases 12 months prior to diagnosis. Other symptoms and signs became significantly associated with being a case closer to the date of diagnosis: shortness of breath and chest pain (3 months prior to diagnosis), lymphadenopathy and finger clubbing (1 month prior) (see Appendix 4).

Secondary analyses

To determine whether the associations were robust to the presence of CRD, we performed a secondary conditional logistic regression that was adjusted for CRD, along with all our matching variables and comorbidity score. The presence of CRD appeared to have no statistically significant effect when directly added as a covariate (OR: 1.05, 95%CI: (0.81, 1.36, $p = 0.7229$, see Appendices 5 & 6).

For peer review only - http://bmjopen.bmj.com/site/about/guidelines.xhtml

Discussion

Main findings

This is the first case-control study in the US to use routine, prospectively collected EHR data to describe the frequency of symptoms and signs of lung cancer and estimate associations with incident lung cancer cases compared to non-lung cancer patients receiving routine ambulatory care in the same time period. Our findings provide unique information on symptoms and signs associated with a higher chance of a patient in ambulatory care being diagnosed with lung cancer, and the duration of these associations prior to their cancer diagnosis. In contrast to prior work on national databases, extracting clinicians' documentation of clinical features from their free text clinical notes using NLP provided more complete symptom identification data, rather than relying on data available only in coded, structured data collected in routine care. Our findings provide evidence-based, quantitative support for the development of decision rules around the diagnostic workup of symptomatic patients, which could lead to the improvement of earlier diagnosis of lung cancer. Of the 22 symptoms and signs studied, 11 were found in adjusted models to be associated with a higher chance of being a lung cancer case, and most of these 11 were present and still significantly associated up to 12 months prior to diagnosis; this suggests opportunities for improved screening practices that may lead to earlier diagnosis and possibly improved outcomes.

Our findings also suggest that the clinical presentation of lung cancer appears to be similar, regardless of the presence of other comorbidities, CRD, or smoking. For patients and clinicians this is important as several of the symptoms or signs we identified may currently be dismissed as being attributable to underlying smoking or comorbid conditions.

Comparison with existing literature

Several of the symptoms and signs we found as having statistically significant odds ratios have been identified in studies using data from ambulatory care in other healthcare systems, especially hemoptysis and cough. However, among the symptoms and signs Hamilton and colleagues (2005) found to be associated with being a lung cancer case in the United Kingdom (UK), loss of appetite had the highest OR (86.0), whereas we failed to identify an association

For peer review only - http://bmjopen.bmj.com/site/about/guidelines.xhtml

with lung cancer.[5] This may be due to a difference in study populations or our use of NLP in EHR data.

Our findings also provide evidence of the temporality of a 'clinical signal' for lung cancer based on symptoms and signs documented in the EHR, at least six and up to 12 months prior to diagnosis, consistent with a Medicare claims study. Data from our study and Nadpara and colleagues' (2015) study, which used claims data, provide evidence for time intervals from first presentation with symptoms to diagnosis that are on the upper range (six months) of those reported using analysis of coded symptoms in primary care databases in several UK and European studies.[8] These describe the overall time interval from first symptom recording in medical records to diagnosis ranging from 3- to 6-months.[6,22,23] While not directly comparable, qualitative research from patients with lung cancer and caregivers describe changes noticeable to the individual more than 12 months before attending a health care visit.[17,24,25]

Strengths and limitations

Using NLP to extract symptoms and signs from unstructured data allowed us to capture a more complete dataset of symptom presence compared to using coded data alone. We selected cases from an empaneled ambulatory care population, where we expected EHR data would be available for the period of interest in this study and attempted to exclude patients who were attending only for secondary or tertiary care provided at UWM. Controls were randomly selected based on case clinic type, to reduce the possibility of bias, and duration of follow-up time and availability of data for cases and controls were similar, particularly in visit frequency. We used a robust design where we matched 10 controls to 1 case, providing greater power and precision, and matched on smoking so that our analyses could not be confounded based on ever vs. never exposure to smoking.

Limitations included criteria for selection of cases and controls differed slightly. As is customary in incident case-control studies, cases were selected based on a diagnosis date defined as the date of the first lung cancer ICD code in the EHR. In this way, we captured the diagnostic path

For peer review only - http://bmjopen.bmj.com/site/about/guidelines.xhtml

from symptom presentation to diagnosis for all cases. Controls were selected based on having a visit to the matched case clinic type (to account for difference in emergency vs other forms of ambulatory care) within 3 months of the case diagnosis date (to avoid potential seasonal differences in respiratory symptoms), however the timing of control selection does not necessarily reflect a “pathway to diagnosis” for some other condition, just recent routine care. Additionally, because we did not link to SEER for the control population, we were unable to apply two of the case exclusion criteria to our control sample: no current or prior history of lung cancer in SEER, although we did check the UW EHR for concurrent lung-cancer related ICD codes and medical history so this should be rare, and no prior history of tracheal cancer, mesothelioma, Kaposi sarcoma, lymphoma, or leukemia in SEER. Additionally, EHR data can sometimes be subject to misclassification. For example, detailed EHR smoking history may be unreliable and the EHR does not reliably capture health literacy or socioeconomic status; however, we used a very broad definition of smoking (ever vs. never) and used a comorbidity score to control for health status. Finally, availability and timing of symptom data for cases and controls is based on patient interactions with the healthcare system, not a pre-specified protocol of data collection. Patients who have more contact with their providers (which could be due to a range of factors) may have had more data captured.

Implications for clinicians, researchers, policy makers

Differentiating patients who may have symptoms or signs of lung cancer from those attending ambulatory care is a critical and challenging step in the earlier detection of this cancer. Our findings not only identify the ‘red flag’ (highly specific, but infrequent) symptoms and signs that primary care providers should be aware of (e.g., hemoptysis), but also highlight which of a larger range of ‘non-specific’ symptoms and signs should equally raise suspicion such as bone pain and weight loss. Furthermore, our findings support the importance of clinical documentation, and continuity of care to identify and act on sustained changes in patients’ clinical presentations.

For peer review only - http://bmjopen.bmj.com/site/about/guidelines.xhtml

Confirmation of our findings using datasets from other healthcare systems in the U.S. are needed and could be enhanced by more advanced machine learning modelling to incorporate additional clinical variable including quantitative data such as changes in body weight or results of routinely collected laboratory tests, given emerging evidence for associations between weight loss and minor deviations of hemoglobin or platelet count with incident cancer.[26] Given the low uptake of low dose CT screening for lung cancer in the U.S., our findings provide support for revising current priorities to improve early diagnosis of lung cancer.[27]

Conclusions

Patients in ambulatory care settings who are subsequently diagnosed with lung cancer appear to have symptoms and signs that distinguish them from other patients, often months before lung cancer diagnosis. To improve earlier detection of lung cancer, interventions are urgently needed that promote earlier screening based on symptomatic presentations in ambulatory care that may lead to an earlier detection and treatment of lung cancer.

Author Contributions: MT was the Principal Investigator for the study and is its guarantor. MT, MZS, LK, LGK, FMW, RDN, CT, designed the study and supervised its execution. KS, MA, MGP, MZS, HB extracted data from UW Medicine and linked to SEER Cancer Registry. MA, HB, MZS performed the analyses. MY, KL, GT created the natural language annotation tool and extracted free text data. LGK, KS, FF, FMW, RDN, CT, MAA, EAS and MT provided further advice and expertise for study design, clinical guidance, analyses and interpretation of data. MP, LK, MT wrote the manuscript. All authors provided critical comments, edited the manuscript, and approved its final version. All authors have read and agreed to the published version of the manuscript.

Funding: This research was funded by the Gordon and Betty Moore Foundation through Grant GBMF8837 to the University of Washington. This research is linked to the CanTest Collaborative, which is funded by Cancer Research UK [C8640/A23385], of which FMW is Director and RDN and MT are Associate Directors. This research was supported by the Cancer

For peer review only - http://bmjopen.bmj.com/site/about/guidelines.xhtml

Surveillance System of the Fred Hutchinson Cancer Research Center, which is funded by Contract Numbers HHSN261201800004I & N01 PC-2018-00004 from the Surveillance, Epidemiology and End Results (SEER) Program of the National Cancer Institute with additional support from the Fred Hutchinson Cancer Research Center and the State of Washington.

The views expressed are those of the authors and do not necessarily represent the official position of the National Cancer Institute, the National Institute of Health, or Department of Health and Human Services.

Institutional Review Board Statement: The study was conducted according to the guidelines of the Declaration of Helsinki, and was classified as Exempt by the University of Washington Human Subjects Division.

Informed Consent Statement: Not applicable.

Data Sharing Statement: Fully anonymized data may be available on reasonable request to the corresponding author, once appropriate data sharing and ethics approvals have been obtained.

Acknowledgments: We would like to thank the patients and clinicians at University of Washington Medicine.

Conflicts of Interest: The authors declare no conflict of interest.

For peer review only

References

1. Centers for Diseases Control and Prevention. Leading cancer cases and deaths, all Races/Ethnicities, male and female, 2018. Accessed January 16, 2022. https://gis.cdc.gov/grasp/USCS/DataViz.html

2. American Lung Association. State of Lung Cancer 2020 Report. Published online 2020:15.

For peer review only - http://bmjopen.bmj.com/site/about/guidelines.xhtml

3. Fedewa SA, Bandi P, Smith RA, Silvestri GA, Jemal A. Lung Cancer Screening Rates During the COVID-19 Pandemic. *Chest*. Published online July 2021:S0012369221013647. doi:10.1016/j.chest.2021.07.030

4. The National Lung Screening Trial Research Team. Reduced Lung-Cancer Mortality with Low-Dose Computed Tomographic Screening. *N Engl J Med*. 2011;365(5):395-409. doi:10.1056/NEJMoa1102873

5. Hamilton W. What are the clinical features of lung cancer before the diagnosis is made? A population based case-control study. *Thorax*. 2005;60(12):1059-1065. doi:10.1136/thx.2005.045880

6. Walter FM, Rubin G, Bankhead C, et al. Symptoms and other factors associated with time to diagnosis and stage of lung cancer: a prospective cohort study. *Br J Cancer*. 2015;112(S1):S6-S13. doi:10.1038/bjc.2015.30

7. Koo MM, Hamilton W, Walter FM, Rubin GP, Lyratzopoulos G. Symptom Signatures and Diagnostic Timeliness in Cancer Patients: A Review of Current Evidence. *Neoplasia*. 2018;20(2):165-174. doi:10.1016/j.neo.2017.11.005

8. Nadpara PA, Madhavan SS, Tworek C, Sambamoorthi U, Hendryx M, Almubarak M. Guideline-concordant lung cancer care and associated health outcomes among elderly patients in the United States. *J Geriatr Oncol*. 2015;6(2):101-110. doi:10.1016/j.jgo.2015.01.001

9. Cancer Statistics Review, 1975-2018 - SEER Statistics. Accessed January 16, 2022. https://seer.cancer.gov/csr/1975_2018/

10. Elixhauser A, Steiner C, Harris DR, Coffey RM. Comorbidity Measures for Use with Administrative Data: *Med Care*. 1998;36(1):8-27. doi:10.1097/00005650-199801000-00004

11. van Walraven C, Austin PC, Jennings A, Quan H, Forster AJ. A Modification of the Elixhauser Comorbidity Measures Into a Point System for Hospital Death Using Administrative Data. *Med Care*. 2009;47(6):626-633. doi:10.1097/MLR.0b013e31819432e5

12. Thompson NR, Fan Y, Dalton JE, et al. A New Elixhauser-based Comorbidity Summary Measure to Predict In-Hospital Mortality. *Med Care*. 2015;53(4):374-379. doi:10.1097/MLR.0000000000000326

13. Hippisley-Cox J, Coupland C. Symptoms and risk factors to identify men with suspected cancer in primary care: derivation and validation of an algorithm. *Br J Gen Pract*. 2013;63(606):e1-e10. doi:10.3399/bjgp13X660724

14. Gould MK, Ghaus SJ, Olsson JK, Schultz EM. Timeliness of Care in Veterans With Non-small Cell Lung Cancer. *Chest*. 2008;133(5):1167-1173. doi:10.1378/chest.07-2654

For peer review only

15. Ades AE, Biswas M, Welton NJ, Hamilton W. Symptom lead time distribution in lung cancer: natural history and prospects for early diagnosis. *Int J Epidemiol*. 2014;43(6):1865-1873. doi:10.1093/ije/dyu174

16. Redaniel MT, Martin RM, Ridd MJ, Wade J, Jeffreys M. Diagnostic Intervals and Its Association with Breast, Prostate, Lung and Colorectal Cancer Survival in England: Historical Cohort Study Using the Clinical Practice Research Datalink. Metze K, ed. *PLOS ONE*. 2015;10(5):e0126608. doi:10.1371/journal.pone.0126608

17. Corner J, Hopkinson J, Fitzsimmons D, Barclay S, Muers M. Is late diagnosis of lung cancer inevitable? Interview study of patients' recollections of symptoms before diagnosis. *Thorax*. 2005;60(4):314-319. doi:10.1136/thx.2004.029264

18. Tod AM, Craven J, Allmark P. Diagnostic delay in lung cancer: a qualitative study: Diagnostic delay in lung cancer. *J Adv Nurs*. 2008;61(3):336-343. doi:10.1111/j.1365-2648.2007.04542.x

19. Lybarger K, Ostendorf M, Thompson M, Yetisgen M. Extracting COVID-19 diagnoses and symptoms from clinical text: A new annotated corpus and neural event extraction framework. *J Biomed Inform*. 2021;117:103761. doi:10.1016/j.jbi.2021.103761

20. Grace Turner, J Chang, Nianiella Dorvall, et al. Domain Adaptation of a Deep Learning Symptom Extractor for Different Patient Populations and Clinical Settings. In: *AMIA 2022 Informatics Summit*.

21. von Elm E, Altman DG, Egger M, Pocock SJ, Gøtzsche PC, Vandenbroucke JP. The Strengthening the Reporting of Observational Studies in Epidemiology (STROBE) Statement: Guidelines for reporting observational studies. *Int J Surg*. 2014;12(12):1495-1499. doi:10.1016/j.ijsu.2014.07.013

22. Ellis PM, Vandermeer R. Delays in the diagnosis of lung cancer. *J Thorac Dis*. 2011;3(3):183-188. doi:10.3978/j.issn.2072-1439.2011.01.01

23. Koyi H, Hillerdal G, Brandén E. Patient's and doctors' delays in the diagnosis of chest tumors. *Lung Cancer*. 2002;35(1):53-57. doi:10.1016/S0169-5002(01)00293-8

24. Al Achkar M, Zigman Suchsland M, Walter FM, Neal RD, Goulart BHL, Thompson MJ. Experiences along the diagnostic pathway for patients with advanced lung cancer in the USA: a qualitative study. *BMJ Open*. 2021;11(4):e045056. doi:10.1136/bmjopen-2020-045056

25. Corner J, Hopkinson J, Roffe L. Experience of health changes and reasons for delay in seeking care: a UK study of the months prior to the diagnosis of lung cancer. *Soc Sci Med 1982*. 2006;62(6):1381-1391. doi:10.1016/j.socscimed.2005.08.012

26. Nicholson BD, Aveyard P, Koshiaris C, et al. Combining simple blood tests to identify primary care patients with unexpected weight loss for cancer investigation: Clinical risk score

For peer review only

For peer review only - http://bmjopen.bmj.com/site/about/guidelines.xhtml

development, internal validation, and net benefit analysis. *PLOS Med*. 2021;18(8):e1003728. doi:10.1371/journal.pmed.1003728

27. Sarma EA, Kobrin SC, Thompson MJ. A Proposal to Improve the Early Diagnosis of Symptomatic Cancers in the United States. *Cancer Prev Res (Phila Pa)*. 2020;13(9):715-720. doi:10.1158/1940-6207.CAPR-20-0115

For peer review only

For peer review only - http://bmjopen.bmj.com/site/about/guidelines.xhtml

Symptoms and signs of lung cancer prior to diagnosis: Comparative study using natural language processing of electronic health records

Figure 1. Flow chart of case and control selection

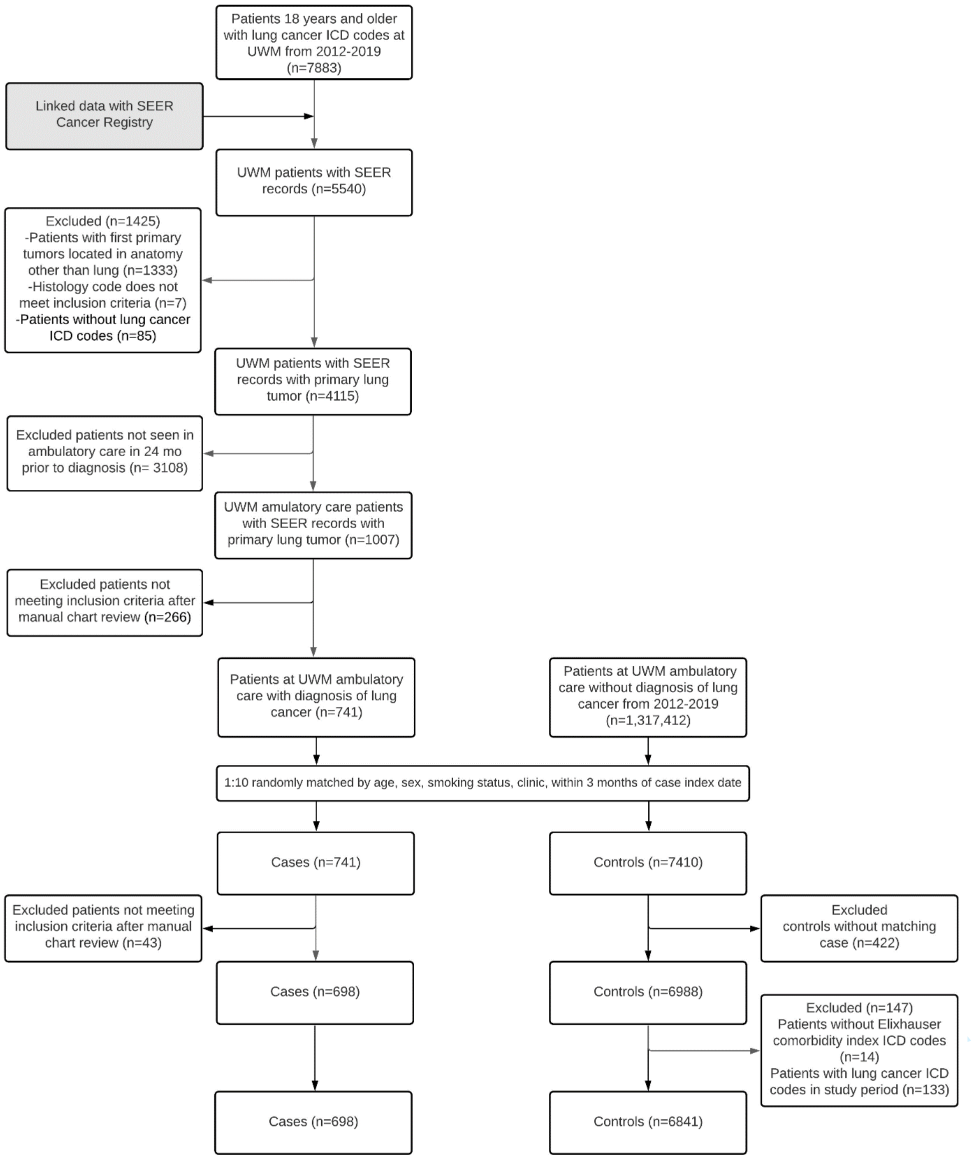

Table 1. Characteristics of patients with lung cancer (cases) and matched controls in ambulatory care

Characteristic	Cases (n=698)	Controls (n=6841)
**Age, years**		
<60	161 (23.1%)	1479 (21.6%)
60-69	257 (36.8%)	2514 (36.7%)
70-79	183 (26.2%)	1865 (27.3%)
80+	97 (13.9%)	983 (14.4%)
**Race**		
American Indian or Alaska Native	6 (0.9%)	78 (1.1%)
Asian	76 (10.9%)	535 (7.8%)
Black or African American	69 (9.9%)	525 (7.7%)
Multiple races	5 (0.7%)	44 (0.6%)
Native Hawaiian or Other Pacific Islander	4 (0.6%)	40 (0.6%)
Unknown	11 (1.6%)	442 (6.5%)
White	527 (75.5%)	5177 (75.7%)
**Ethnicity**		
Hispanic or Latino	23 (3.3%)	244 (3.6%)
Not Hispanic or Latino	630 (90.3%)	5782 (84.5%)
Unknown	45 (6.4%)	815 (11.9%)
**Sex**		
Male	353 (50.6%)	3452 (50.5%)
**Comorbidity - Elixhauser van Walraven weighted Score, mean (SD)**	14.9 (11.6)	4.4 (8.6)
**Number of clinic visits per patient, median (IQR)**		
In entire data window prior to diagnosis/index	51.0 (28.0 - 97.8)	23.0 (9.0 - 53.0)
In 1st quarter prior to diagnosis/index	21.0 (12.0 - 35.0)	5.0 (2.0 - 11.0)
In 2nd quarter prior to diagnosis/index	7.0 (3.0 - 14.0)	5.0 (2.0 - 11.0)
In 3rd quarter prior to diagnosis/index	7.0 (3.0 - 12.0)	5.0 (2.0 - 11.0)
In 4th quarter prior to diagnosis/index	6.0 (3.0 - 13.0)	5.0 (2.0 - 11.0)

For peer review only

Table 2. Comparison of frequency of symptoms and signs identified in coded or free-text data in cases compared to controls

Symptom or sign	Cases (n=698)	Controls (n=6841)
Cough	573 (82.1%)	1654 (24.2%)
Shortness of breath	515 (73.8%)	1613 (23.6%)
Fatigue	476 (68.2%)	1587 (23.2%)
Ankle swelling	447 (64.0%)	1838 (26.9%)
Chest Pain	403 (57.7%)	1401 (20.5%)
Chest crackles or wheeze	397 (56.9%)	575 (8.4%)
Back pain	350 (50.1%)	946 (13.8%)
Change in bowel habits	336 (48.1%)	1155 (16.9%)
Muscle weakness	334 (47.9%)	1102 (16.1%)
Fever	322 (46.1%)	1334 (19.5%)
Weight loss	308 (44.1%)	522 (7.6%)
Headache	304 (43.6%)	1205 (17.6%)
Dizziness	299 (42.8%)	1319 (19.3%)
Bone pain	270 (38.7%)	725 (10.6%)
Lack of appetite	196 (28.1%)	457 (6.7%)
Shoulder pain	180 (25.8%)	713 (10.4%)
Lympadenopathy	151 (21.6%)	105 (1.5%)
Night sweats	150 (21.5%)	371 (5.4%)
Changes in sleep	134 (19.2%)	631 (9.2%)
Hemoptysis	115 (16.5%)	67 (1.0%)
Hoarseness	67 (9.6%)	133 (1.9%)
Finger clubbing	39 (5.6%)	2 (0.0%)

For peer review only

Table 3. Univariate and multivariate analyses of symptoms and signs identified in coded or free-text data of cases compared to controls, adjusted for comorbidity (descending order by multivariate odds ratios)

Symptom or sign	Univariate Odds ratio (95%CI)	Multivariate Odds ratio (95%CI)	Multivariate P value
Finger clubbing	175.7 (40.1 - 770.0)*	50.1 (8.9 - 283.3)	<0.0001
Lymphadenopathy	9.4 (6.9 - 12.8)*	5.8 (3.8 - 8.8)	<0.0001
Cough	11.1 (8.8 - 13.9)*	4.7 (3.5 - 6.3)	<0.0001
Hemoptysis	14.5 (10.2 - 20.8)*	3.5 (2.2 - 5.5)	<0.0001
Chest crackles or wheeze	9.9 (8.1 - 12.2)*	3.2 (2.4 - 4.3)	<0.0001
Weight loss	5.9 (4.8 - 7.2)*	2.9 (2.2 - 3.9)	<0.0001
Back pain	4.7 (3.9 - 5.7)*	2.4 (1.8 - 3.1)	<0.0001
Bone pain	4.6 (3.8 - 5.7)*	2.3 (1.7 - 3.1)	<0.0001
Shortness of breath	6.0 (4.9 - 7.3)*	1.9 (1.4 - 2.5)	<0.0001
Fatigue	4.8 (4.0 - 5.8)*	1.8 (1.4 - 2.4)	<0.0001
Chest Pain	3.6 (3.0 - 4.3)*	1.4 (1.1 - 1.8)	0.0118
Shoulder pain	2.3 (1.8 - 2.8)*	1.3 (1.0 - 1.7)	0.1111
Ankle swelling	3.3 (2.7 - 4.0)*	1.1 (0.9 - 1.5)	0.3643
Headache	2.5 (2.1 - 3.0)*	1.1 (0.8 - 1.4)	0.5619
Hoarseness	3.5 (2.5 - 5.0)*	1.1 (0.7 - 1.7)	0.8447
Change in bowel habits	3.0 (2.5 - 3.6)*	1.0 (0.8 - 1.4)	0.8880
Muscle weakness	2.9 (2.4 - 3.5)*	1.0 (0.7 - 1.3)	0.9581
Night sweats	3.3 (2.6 - 4.2)*	0.8 (0.6 - 1.2)	0.2998
Lack of appetite	2.6 (2.1 - 3.3)*	0.7 (0.5 - 0.9)	0.0193
Dizziness	2.0 (1.7 - 2.4)*	0.6 (0.4 - 0.8)	0.0004
Changes in sleep	1.3 (1.1 - 1.7)*	0.5 (0.3 - 0.6)	<0.0001
Fever	2.1 (1.7 - 2.5)*	0.4 (0.3 - 0.6)	<0.0001

Note: Conditional logistic regression models adjusted for comorbidities using van Walraven weighted score with each symptom or sign modeled individually (univariate) and mutually adjusted (multivariate)
*Significant at p<0.0001 for univariate analysis

For peer review only

Figure 2: Multivariable analysis of symptoms or signs of cases compared to controls with symptom and sign data excluded from 1, 3, 6, and 12 months prior to diagnosis/index date

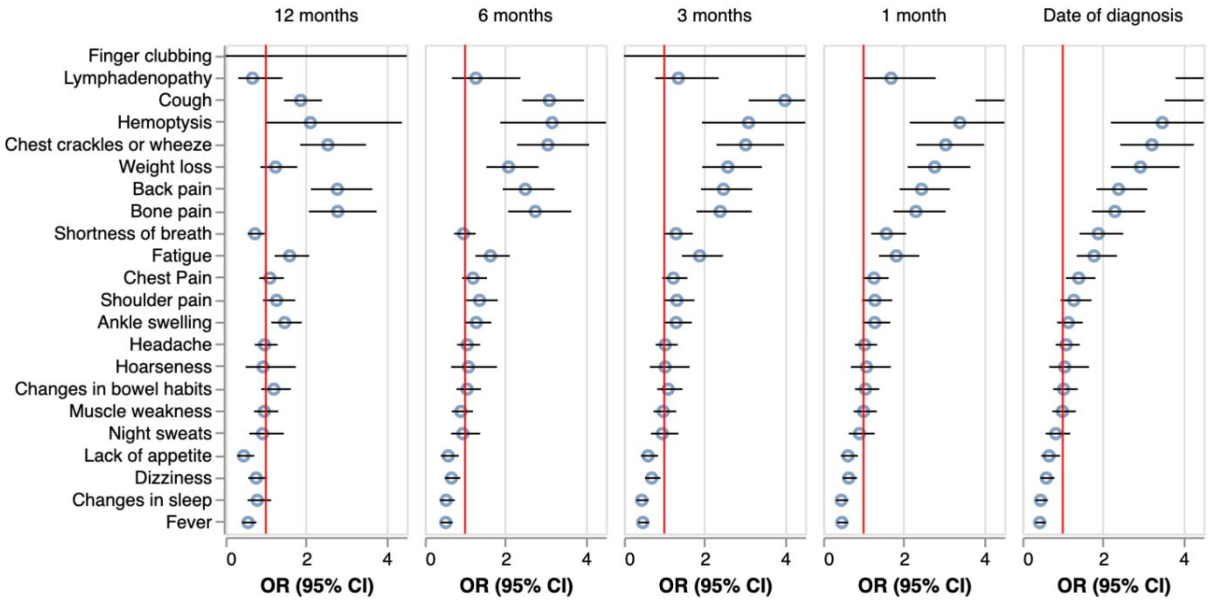

Note: Mutual adjustment of all symptoms and signs in using a conditional logistic regression model stratified by time prior to date of diagnosis. Models additionally adjusted for comorbidities using van Walraven weighted score.

For peer review only

Symptoms and signs of lung cancer prior to diagnosis: Comparative study using natural language processing of electronic health records

Appendix 1. Diagnostic codes used to identify cases of lung cancer

ICD 9: 162.2 – 162.9

- 162.2 - Malignant neoplasm of main bronchus
- 162.3 - Malignant neoplasm of upper lobe, bronchus or lung
- 162.4 - Malignant neoplasm of middle lobe, bronchus or lung
- 162.5 - Malignant neoplasm of lower lobe, bronchus or lung
- 162.8 - Malignant neoplasm of other parts of bronchus or lung
- 162.9 - Malignant neoplasm of bronchus and lung, unspecified

ICD 10: C34.0 – C34.9

- C34.0 - Malignant neoplasm of main bronchus
- C34.00 - Malignant neoplasm of unspecified main bronchus
- C34.01 - Malignant neoplasm of right main bronchus
- C34.02 - Malignant neoplasm of left main bronchus
- C34.1 - Malignant neoplasm of upper lobe, bronchus or lung
- C34.10 - Malignant neoplasm of upper lobe, unspecified bronchus or lung
- C34.11 - Malignant neoplasm of upper lobe, right bronchus or lung
- C34.12 - Malignant neoplasm of upper lobe, left bronchus or lung
- C34.2 - Malignant neoplasm of middle lobe, bronchus or lung
- C34.3 - Malignant neoplasm of lower lobe, bronchus or lung
- C34.30 - Malignant neoplasm of lower lobe, unspecified bronchus or lung
- C34.31 - Malignant neoplasm of lower lobe, right bronchus or lung
- C34.32 - Malignant neoplasm of lower lobe, left bronchus or lung
- C34.8 - Malignant neoplasm of overlapping sites of bronchus and lung
- C34.80 - Malignant neoplasm of overlapping sites of unspecified bronchus and lung
- C34.81 - Malignant neoplasm of overlapping sites of right bronchus and lung
- C34.82 - Malignant neoplasm of overlapping sites of left bronchus and lung
- C34.9 - Malignant neoplasm of unspecified part of bronchus or lung
- C34.90 - Malignant neoplasm of unspecified part of unspecified bronchus or lung
- C34.91 - Malignant neoplasm of unspecified part of right bronchus or lung
- C34.92 - Malignant neoplasm of unspecified part of left bronchus or lung

Excluded ICD Diagnostic Codes

- ICD-9: 162.0
- ICD-10: C33

Excluded Histology codes

- Mesothelioma: 9050-9055
- Kaposi Sarcoma: 9140
- Lymphoma/leukemia: M9590-M9992

For peer review only

Appendix 2. Symptoms and signs Identified in peer-reviewed literature previously associated with lung cancer in primary care populations

Symptom or sign	ICD 9 code(s)	ICD10 code(s)	References
Ankle swelling	782.3	R60.9	[1]Ellis (2011)
Back pain	724.1	M54.6	[1]Ellis (2011) [2]Molassiotis (2010)
Bone pain	733.9	M85.80	[3]Gould (2008) [4]Nadpara (2015)
Changes in bowel habits	787.99	R19.4	[5]Corner (2005)
Changes in sleep	780.50	G47.9	[5]Corner (2005)
Chest Pain	786.5<br>786.50<br>786.51<br>786.52<br>786.59	R07.9<br>R07.81	[1]Ellis (2011) [4]Nadpara (2015) [5]Corner (2005) [6]Chowienczyk, Hamilton (2020) [7]Walter (2015) [8]Hamilton (2005) [9]Ades (2014) [10]Redaniel (2015) [11]Tod (2008) [12]Mitchell (2013)
Chest crackles or wheeze	786.7	R09.89	[10]Redaniel (2015)
Cough	786.2<br>491.0	R05	[1]Ellis (2011) [2]Molassiotis (2010) [4]Nadpara (2015) [5]Corner (2005) [6]Chowienczyk, Hamilton (2020) [7]Walter (2015) [9]Ades (2014) [10]Redaniel (2015) [11]Tod (2008) [12]Mitchell (2013) [13]Menon (2019)
Dizziness	780.4	R42	[2]Molassiotis (2010)
Fatigue/tiredness	780.79	R53.81<br>R53.8<br>R53.83<br>R53.1	[1]Ellis (2011) [2]Molassiotis (2010) [5]Corner (2005) [6]Chowienczyk, Hamilton (2020) [7]Walter (2015) [8]Hamilton (2005) [10]Redaniel (2015) [11]Tod (2008) [13]Menon (2019)
Fever	780.6<br>780.60	R50.9	[4]Nadpara (2015)
Finger clubbing	781.5	R68.3	[4]Nadpara (2015) [8]Hamilton (2005) [10]Redaniel (2015)
Headache	784.0	R51	[1]Ellis (2011)
Hemoptysis	786.3<br>786.30<br>786.39	R04.2	[1]Ellis (2011) [4]Nadpara (2015) [5]Corner [6]Chowienczyk, Hamilton (2020) [7]Walter (2015) [8]Hamilton (2005) [10]Redaniel (2015) (2005) [11]Tod (2008) [12]Mitchell (2013) [13]Menon (2019) [14]Hippisley-Cox (2011)

For peer review only

Hoarseness	784.49<br>784.42	R49.8<br>R49.0	[1]Ellis (2011) [2]Molassiotis (2010) [7]Walter (2015) [10]Redaniel (2015) [11]Tod (2008) [12]Mitchell (2013)
Lack of appetite	783	R63.0	[1]Ellis (2011) [2]Molassiotis (2010) [5]Corner (2005) [6]Chowienczyk, Hamilton (2020) [7]Walter (2015) [8]Hamilton (2005) [13]Menon (2019)
Lympadenopathy	785.6	R59.9	[10]Redaniel (2015) [12]Mitchell (2013)
Muscle weakness	728.87	M62.81	[4]Nadpara (2015) [12]Mitchell (2013)
Night sweats	780.8	R61	[3]Gould (2008) [5]Corner (2005)
Shortness of breath	786.05<br>786.0<br>786.9	R06.02<br>R06.00<br>R06.09	[1]Ellis (2011) [2]Molassiotis (2010) [4]Nadpara (2015) [5]Corner (2005) [6]Chowienczyk, Hamilton (2020) [7]Walter (2015) [8]Hamilton (2005) [10]Redaniel (2015) [12]Mitchell (2013) [13]Menon (2019)
Shoulder pain	719.41	M25.511<br>M25.512<br>M25.519	[10]Redaniel (2015) [12]Mitchell (2013)
Weight loss	783.21	R63.4	[1]Ellis (2011) [4]Nadpara (2015) [5]Corner (2005) [6]Chowienczyk, Hamilton (2020) [7]Walter (2015) [8]Hamilton (2005) [10]Redaniel (2015) [11]Tod (2008) [12]Mitchell (2013)
Wheezing and stridor	786.07<br>786.1	R06.2<br>R06.1	[4]Nadpara (2015) [10]Redaniel (2015)

1. Ellis PM, Vandermeer R. Delays in the diagnosis of lung cancer. *J Thorac Dis*. 2011;3(3):183-188. doi:10.3978/j.issn.2072-1439.2011.01.01

2. Molassiotis A, Wilson B, Brunton L, Chandler C. Mapping patients' experiences from initial change in health to cancer diagnosis: a qualitative exploration of patient and system factors mediating this process. *Eur J Cancer Care (Engl)*. 2010;19(1):98-109. doi:10.1111/j.1365-2354.2008.01020.x

3. Gould MK, Ghaus SJ, Olsson JK, Schultz EM. Timeliness of Care in Veterans With Non-small Cell Lung Cancer. *Chest*. 2008;133(5):1167-1173. doi:10.1378/chest.07-2654

4. Nadpara PA, Madhavan SS, Tworek C, Sambamoorthi U, Hendryx M, Almubarak M. Guideline-concordant lung cancer care and associated health outcomes among elderly patients in the United States. *J Geriatr Oncol*. 2015;6(2):101-110. doi:10.1016/j.jgo.2015.01.001

5. Corner J, Hopkinson J, Fitzsimmons D, Barclay S, Muers M. Is late diagnosis of lung cancer inevitable? Interview study of patients' recollections of symptoms before diagnosis. *Thorax*. 2005;60(4):314-319. doi:10.1136/thx.2004.029264

For peer review only

6. Chowienczyk S, Price S, Hamilton W. Changes in the presenting symptoms of lung cancer from 2000–2017: a serial cross-sectional study of observational records in UK primary care. *Br J Gen Pract*. 2020;70(692):e193-e199. doi:10.3399/bjgp20X708137

7. Walter FM, Rubin G, Bankhead C, et al. Symptoms and other factors associated with time to diagnosis and stage of lung cancer: a prospective cohort study. *Br J Cancer*. 2015;112(S1):S6-S13. doi:10.1038/bjc.2015.30

8. Hamilton W. What are the clinical features of lung cancer before the diagnosis is made? A population based case-control study. *Thorax*. 2005;60(12):1059-1065. doi:10.1136/thx.2005.045880

9. Ades AE, Biswas M, Welton NJ, Hamilton W. Symptom lead time distribution in lung cancer: natural history and prospects for early diagnosis. *Int J Epidemiol*. 2014;43(6):1865-1873. doi:10.1093/ije/dyu174

10. Redaniel MT, Martin RM, Ridd MJ, Wade J, Jeffreys M. Diagnostic Intervals and Its Association with Breast, Prostate, Lung and Colorectal Cancer Survival in England: Historical Cohort Study Using the Clinical Practice Research Datalink. Metze K, ed. *PLOS ONE*. 2015;10(5):e0126608. doi:10.1371/journal.pone.0126608

11. Tod AM, Craven J, Allmark P. Diagnostic delay in lung cancer: a qualitative study: Diagnostic delay in lung cancer. *J Adv Nurs*. 2008;61(3):336-343. doi:10.1111/j.1365-2648.2007.04542.x

12. Mitchell ED, Rubin G, Macleod U. Understanding diagnosis of lung cancer in primary care: qualitative synthesis of significant event audit reports. *Br J Gen Pract*. 2013;63(606):e37-e46. doi:10.3399/bjgp13X660760

13. Menon U, Vedsted P, Zalounina Falborg A, et al. Time intervals and routes to diagnosis for lung cancer in 10 jurisdictions: cross-sectional study findings from the International Cancer Benchmarking Partnership (ICBP). *BMJ Open*. 2019;9(11):e025895. doi:10.1136/bmjopen-2018-025895

For peer review only

Appendix 3. Comparison of the number of patients with symptoms and signs extracted from the electronic medical record of cases or controls from coded fields versus free-text data using natural language processing (NLP)

Symptom or sign	Identified from NLP (% of patients)	Identified from coded data (% of patients)	Identified from either coded data or NLP (% of patients)	NLP adds (NLP adds n/coded or NLP n)
Cough	1700 (22.6%)	1139 (15.1%)	2227 (29.5%)	1088 (48.9%)
Shortness of breath	1580 (21.0%)	1111 (14.7%)	2128 (28.2%)	1017 (47.8%)
Chest Pain	1241 (16.5%)	981 (13.0%)	1804 (23.9%)	823 (45.6%)
Fatigue	1489 (19.8%)	959 (12.7%)	2063 (27.4%)	1104 (53.5%)
Shoulder pain	513 (6.8%)	594 (7.9%)	893 (11.9%)	299 (33.5%)
Dizziness	1331 (17.7%)	536 (7.1%)	1618 (21.5%)	1082 (66.9%)
Ankle swelling	2081 (27.6%)	509 (6.8%)	2285 (30.3%)	1776 (77.7%)
Headache	1281 (17.0%)	415 (5.5%)	1509 (20.0%)	1094 (72.5%)
Weight loss	646 (8.6%)	328 (4.4%)	830 (11.0%)	502 (60.5%)
Fever	1517 (20.1%)	252 (3.3%)	1656 (22.0%)	1404 (84.8%)
Chest crackles or wheeze	834 (11.1%)	242 (3.2%)	972 (12.9%)	730 (75.1%)
Lympadenopathy	52 (0.7%)	223 (3.0%)	256 (3.4%)	33 (12.9%)
Bone pain	829 (11.0%)	216 (2.9%)	995 (13.2%)	779 (78.3%)
Muscle weakness	1327 (17.6%)	205 (2.7%)	1436 (19.1%)	1231 (85.7%)
Back pain	1220 (16.2%)	154 (2.0%)	1296 (17.2%)	1142 (88.1%)
Changes in sleep	662 (8.8%)	137 (1.8%)	765 (10.2%)	628 (82.1%)
Hoarseness	130 (1.7%)	118 (1.6%)	200 (2.7%)	82 (41.0%)
Hemoptysis	133 (1.8%)	94 (1.3%)	182 (2.4%)	88 (48.4%)
Night sweats	480 (6.4%)	72 (1.0%)	521 (6.9%)	449 (86.2%)
Lack of appetite	626 (8.3%)	59 (0.8%)	653 (8.7%)	594 (91.0%)
Change in bowel habits	1465 (19.4%)	59 (0.8%)	1491 (19.8%)	1432 (96.0%)
Finger clubbing	41 (0.5%)	1 (0.0%)	41 (0.5%)	40 (97.6%)

For peer review only

Appendix 4. Multivariable analysis of symptoms or signs of cases compared to controls at 1, 3, 6 and 12 months prior to diagnosis/index date

	12 months	6 months	3 months	1 month	At diagnosis
**Symptom or sign**	**OR**	**OR**	**OR**	**OR**	**OR**
**Finger clubbing**	>1,000 (0.0 - >1,000)	>1,000 (0.0 - >1,000)	>1,000 (0.0 - >1,000)	60.7 (10.6 - 348.7)***	50.1 (8.9 - 283.3)***
**Lymphadenopathy**	0.7 (0.3 - 1.4)	1.3 (0.7 - 2.4)	1.3 (0.8 - 2.3)	1.7 (1.0 - 2.8)*	5.8 (3.8 - 8.8)***
**Cough**	1.9 (1.5 - 2.4)***	3.1 (2.4 - 4.0)***	4.0 (3.1 - 5.2)***	5.0 (3.8 - 6.5)***	4.7 (3.5 - 6.3)***
**Hemoptysis**	2.1 (1.0 - 4.4)*	3.2 (1.9 - 5.3)***	3.1 (1.9 - 4.9)***	3.4 (2.2 - 5.4)***	3.5 (2.2 - 5.5)***
**Chest crackles or wheeze**	2.5 (1.9 - 3.5)***	3.1 (2.3 - 4.1)***	3.0 (2.3 - 4.0)***	3.0 (2.3 - 4.0)***	3.2 (2.4 - 4.3)***
**Weight loss**	1.2 (0.9 - 1.8)	2.1 (1.5 - 2.8)***	2.6 (1.9 - 3.4)***	2.8 (2.1 - 3.7)***	2.9 (2.2 - 3.9)***
**Back pain**	2.8 (2.1 - 3.6)***	2.5 (1.9 - 3.2)***	2.5 (1.9 - 3.2)***	2.4 (1.9 - 3.1)***	2.4 (1.8 - 3.1)***
**Bone pain**	2.8 (2.1 - 3.7)***	2.7 (2.1 - 3.6)***	2.4 (1.8 - 3.2)***	2.3 (1.7 - 3.0)***	2.3 (1.7 - 3.0)***
**Shortness of breath**	0.7 (0.5 - 1.0)*	1.0 (0.7 - 1.3)	1.3 (1.0 - 1.7)	1.6 (1.2 - 2.1)**	1.9 (1.4 - 2.5)***
**Fatigue**	1.6 (1.2 - 2.1)***	1.6 (1.3 - 2.1)***	1.9 (1.4 - 2.5)***	1.8 (1.4 - 2.4)***	1.8 (1.3 - 2.3)***
**Chest Pain**	1.1 (0.8 - 1.4)	1.2 (0.9 - 1.5)	1.2 (1.0 - 1.6)	1.3 (1.0 - 1.6)	1.4 (1.1 - 1.8)*
**Shoulder pain**	1.3 (0.9 - 1.7)	1.4 (1.0 - 1.8)*	1.3 (1.0 - 1.7)	1.3 (1.0 - 1.7)	1.3 (0.9 - 1.7)
**Ankle swelling**	1.5 (1.1 - 1.9)**	1.3 (1.0 - 1.7)	1.3 (1.0 - 1.7)	1.3 (1.0 - 1.7)	1.1 (0.9 - 1.5)
**Headache**	1.0 (0.7 - 1.3)	1.1 (0.8 - 1.4)	1.0 (0.8 - 1.3)	1.0 (0.8 - 1.3)	1.1 (0.8 - 1.4)
**Hoarseness**	0.9 (0.5 - 1.7)	1.1 (0.7 - 1.8)	1.0 (0.6 - 1.6)	1.1 (0.7 - 1.7)	1.0 (0.7 - 1.7)
**Changes in bowel habits**	1.2 (0.9 - 1.6)	1.0 (0.8 - 1.4)	1.1 (0.8 - 1.5)	1.0 (0.8 - 1.4)	1.0 (0.8 - 1.4)
**Muscle weakness**	1.0 (0.7 - 1.3)	0.9 (0.7 - 1.2)	1.0 (0.7 - 1.3)	1.0 (0.8 - 1.3)	1.0 (0.7 - 1.3)
**Night sweats**	0.9 (0.6 - 1.4)	0.9 (0.7 - 1.4)	0.9 (0.7 - 1.3)	0.9 (0.6 - 1.3)	0.8 (0.6 - 1.2)
**Lack of appetite**	0.5 (0.3 - 0.7)***	0.6 (0.4 - 0.8)**	0.6 (0.4 - 0.8)**	0.6 (0.4 - 0.9)**	0.7 (0.5 - 0.9)*
**Dizziness**	0.8 (0.6 - 1.0)	0.7 (0.5 - 0.9)**	0.7 (0.5 - 0.9)**	0.6 (0.5 - 0.8)**	0.6 (0.4 - 0.8)***
**Changes in sleep**	0.8 (0.5 - 1.1)	0.5 (0.4 - 0.7)***	0.4 (0.3 - 0.6)***	0.4 (0.3 - 0.6)***	0.4 (0.3 - 0.6)***
**Fever**	0.6 (0.4 - 0.8)***	0.5 (0.4 - 0.7)***	0.5 (0.4 - 0.6)***	0.5 (0.3 - 0.6)***	0.4 (0.3 - 0.6)***

Note: Models adjusted for comorbidities using van Walraven weighted score. Confidence intervals for significant ORs do not incorporate 1.0 due to rounding.

* p<0.05

** p<0.01

*** p<0.001

For peer review only

Appendix 5. Frequency of symptoms and signs in cases and controls with and without chronic respiratory disease

Symptom or sign	Chronic respiratory disease		No chronic respiratory disease	
	Control (n=1252)	Case (n=353)	Control (n=5589)	Case (n=345)
Cough	636 (50.8%)	312 (88.4%)	1018 (18.2%)	261 (75.7%)
Shortness of breath	623 (49.8%)	307 (87.0%)	990 (17.7%)	208 (60.3%)
Fatigue	459 (36.7%)	266 (75.4%)	1128 (20.2%)	210 (60.9%)
Ankle swelling	516 (41.2%)	250 (70.8%)	1322 (23.7%)	197 (57.1%)
Chest Pain	439 (35.1%)	228 (64.6%)	962 (17.2%)	175 (50.7%)
Chest crackles or wheeze	307 (24.5%)	268 (75.9%)	268 (4.8%)	129 (37.4%)
Back pain	278 (22.2%)	191 (54.1%)	668 (12.0%)	159 (46.1%)
Changes in bowel habits	337 (26.9%)	195 (55.2%)	818 (14.6%)	141 (40.9%)
Muscle weakness	327 (26.1%)	177 (50.1%)	775 (13.9%)	157 (45.5%)
Fever	433 (34.6%)	177 (50.1%)	901 (16.1%)	145 (42.0%)
Weight loss	165 (13.2%)	191 (54.1%)	357 (6.4%)	117 (33.9%)
Headache	324 (25.9%)	175 (49.6%)	881 (15.8%)	129 (37.4%)
Dizziness	366 (29.2%)	174 (49.3%)	953 (17.1%)	125 (36.2%)
Bone pain	207 (16.5%)	141 (39.9%)	518 (9.3%)	129 (37.4%)
Lack of appetite	142 (11.3%)	116 (32.9%)	315 (5.6%)	80 (23.2%)
Shoulder pain	200 (16.0%)	92 (26.1%)	513 (9.2%)	88 (25.5%)
Lymphadenopathy	35 (2.8%)	79 (22.4%)	70 (1.3%)	72 (20.9%)
Night sweats	113 (9.0%)	89 (25.2%)	258 (4.6%)	61 (17.7%)
Changes in sleep	178 (14.2%)	90 (25.5%)	453 (8.1%)	44 (12.8%)
Hemoptysis	31 (2.5%)	72 (20.4%)	36 (0.6%)	43 (12.5%)
Hoarseness	55 (4.4%)	45 (12.7%)	78 (1.4%)	22 (6.4%)
Finger clubbing	1 (0.1%)	28 (7.9%)	1 (0.0%)	11 (3.2%)

For peer review only

Appendix 6. Multivariate analysis of symptoms and signs in patients with and without chronic respiratory disease

Symptom or sign	Chronic respiratory disease: Univariate Odds ratio (95%CI)	Chronic respiratory disease: Multivariate Odds ratio (95%CI)	Chronic respiratory disease: Multivariate P value	No chronic respiratory disease: Univariate Odds ratio (95%CI)	No chronic respiratory disease: Multivariate Odds ratio (95%CI)	No chronic respiratory disease: Multivariate P value
Finger clubbing	47.3 (6.1 - 364.5)	17.8 (1.3 - 247.1)	0.0322	>1,000 (0.0 - >1,000)	267.7 (0.1 - >1,000)	0.1783
Chest crackles or wheeze	9.4 (6.3 - 14.2)*	4.9 (2.6 - 9.0)	<0.0001	9.8 (7.0 - 13.9)*	3.2 (2.0 - 5.2)	<0.0001
Hemoptysis	12.5 (6.2 - 25.3)*	4.4 (1.7 - 11.5)	0.0028	20.3 (10.2 - 40.5)*	3.8 (1.5 - 9.8)	0.0049
Weight loss	7.1 (4.7 - 10.5)*	4.0 (2.2 - 7.4)	<0.0001	3.8 (2.8 - 5.3)*	1.6 (1.0 - 2.5)	0.0643
Lympadenopathy	7.1 (3.9 - 13.0)*	3.3 (1.3 - 7.9)	0.0089	12.0 (7.2 - 19.9)*	8.5 (4.3 - 17.0)	<0.0001
Fatigue	5.2 (3.6 - 7.6)*	2.9 (1.6 - 5.5)	0.0008	4.2 (3.2 - 5.6)*	1.7 (1.1 - 2.6)	0.0128
Back pain	4.6 (3.2 - 6.6)*	2.4 (1.4 - 4.1)	0.0014	4.8 (3.6 - 6.4)*	2.1 (1.4 - 3.2)	0.0003
Cough	6.5 (4.2 - 10.2)*	2.2 (1.1 - 4.3)	0.0189	12.2 (9.0 - 16.6)*	6.3 (4.2 - 9.3)	<0.0001
Bone pain	3.8 (2.6 - 5.5)*	2.1 (1.1 - 4.0)	0.0168	5.3 (3.9 - 7.2)*	2.5 (1.6 - 3.9)	0.0001
Shortness of breath	6.5 (4.1 - 10.3)*	1.6 (0.8 - 3.2)	0.1688	5.1 (3.9 - 6.7)*	1.9 (1.3 - 2.9)	0.0024
Changes in bowel habits	2.7 (2.0 - 3.8)*	1.3 (0.7 - 2.3)	0.4474	2.5 (1.9 - 3.4)*	0.9 (0.6 - 1.4)	0.7286
Night sweats	3.1 (2.1 - 4.7)*	1.2 (0.6 - 2.4)	0.5393	3.8 (2.6 - 5.7)*	0.9 (0.5 - 1.7)	0.8542
Ankle swelling	2.8 (2.0 - 3.9)*	1.1 (0.6 - 2.0)	0.6696	3.1 (2.4 - 4.0)*	1.2 (0.8 - 1.8)	0.3121
Shoulder pain	1.6 (1.1 - 2.4)	1.1 (0.6 - 2.0)	0.7589	2.9 (2.1 - 4.0)*	1.6 (1.0 - 2.5)	0.0484
Hoarseness	2.5 (1.4 - 4.4)	1.0 (0.5 - 2.3)	0.9617	4.1 (2.2 - 7.7)*	0.9 (0.4 - 2.2)	0.8729
Headache	2.5 (1.9 - 3.5)*	0.9 (0.5 - 1.7)	0.8551	2.2 (1.7 - 2.9)*	1.0 (0.7 - 1.6)	0.8319
Chest Pain	2.6 (1.9 - 3.6)*	0.9 (0.5 - 1.6)	0.7953	3.7 (2.8 - 4.8)*	1.5 (1.0 - 2.2)	0.0494
Muscle weakness	2.3 (1.7 - 3.2)*	0.9 (0.5 - 1.7)	0.7901	3.1 (2.3 - 4.1)*	1.1 (0.7 - 1.7)	0.6809
Dizziness	2.3 (1.7 - 3.3)*	0.9 (0.5 - 1.6)	0.7450	1.8 (1.3 - 2.4)*	0.5 (0.3 - 0.8)	0.0027
Lack of appetite	2.6 (1.8 - 3.8)*	0.5 (0.3 - 1.0)	0.0667	1.8 (1.3 - 2.6)	0.5 (0.3 - 0.9)	0.0122
Changes in sleep	1.6 (1.1 - 2.3)	0.5 (0.3 - 0.9)	0.0233	1.1 (0.7 - 1.6)	0.3 (0.2 - 0.6)	0.0004
Fever	1.6 (1.2 - 2.2)	0.3 (0.2 - 0.6)	0.0003	2.5 (1.9 - 3.3)*	0.6 (0.4 - 0.9)	0.0229

Note: Models adjusted for comorbidities using van Walraven weighted score

*Significant at p<0.0001

For peer review only

For peer review only

For peer review only

BMJ Open

Symptoms and signs of lung cancer prior to diagnosis: Case-control study using electronic health records from ambulatory care within a large US-based tertiary care center

Journal:	*BMJ Open*
Manuscript ID	bmjopen-2022-068832.R1
Article Type:	Original research
Date Submitted by the Author:	13-Jan-2023
Complete List of Authors:	Prado, Maria G.; University of Washington, Department of Family Medicine<br>Kessler, Larry ; University of Washington, Health Services<br>Au, Margaret A; University of Washington, Department of Family Medicine<br>Burkhardt, Hannah; University of Washington, Department of Biomedical Informatics and Medical Education<br>Zigman Suchsland, Monica; University of Washington<br>Kowalski, Lesleigh; University of Washington, Department of Family Medicine<br>Stephens, KA; University of Washington,<br>Yetisgen, Meliha; University of Washington, Department of Biomedical Informatics and Medical Education<br>Walter, Fiona M; Queen Mary University of London; University of Cambridge, Department of Public Health and Primary Care<br>Neal, Richard; University of Exeter<br>Lybarger, Kevin; University of Washington, Department of Biomedical Informatics and Medical Education<br>Thompson, Caroline; The University of North Carolina at Chapel Hill, Department of Epidemiology; San Diego State University<br>Al Achkar, Morhaf; University of Washington, Family Medicine<br>Sarma, Elizabeth; NIH, National Cancer Institute<br>Turner, Grace; University of Washington, Department of Biomedical Informatics and Medical Education<br>Farjah, Farhood ; University of Washington<br>Thompson, Matthew; University of Washington, Department of Family Medicine
**Primary Subject Heading**:	Oncology
Secondary Subject Heading:	Health informatics, Oncology
Keywords:	Health informatics < BIOTECHNOLOGY & BIOINFORMATICS, Thoracic medicine < INTERNAL MEDICINE, Respiratory tract tumours < ONCOLOGY, PRIMARY CARE

SCHOLARONE™
Manuscripts

I, the Submitting Author has the right to grant and does grant on behalf of all authors of the Work (as defined in the below author licence), an exclusive licence and/or a non-exclusive licence for contributions from authors who are: i) UK Crown employees; ii) where BMJ has agreed a CC-BY licence shall apply, and/or iii) in accordance with the terms applicable for US Federal Government officers or employees acting as part of their official duties; on a worldwide, perpetual, irrevocable, royalty-free basis to BMJ Publishing Group Ltd ("BMJ") its licensees and where the relevant Journal is co-owned by BMJ to the co-owners of the Journal, to publish the Work in this journal and any other BMJ products and to exploit all rights, as set out in our licence.

The Submitting Author accepts and understands that any supply made under these terms is made by BMJ to the Submitting Author unless you are acting as an employee on behalf of your employer or a postgraduate student of an affiliated institution which is paying any applicable article publishing charge ("APC") for Open Access articles. Where the Submitting Author wishes to make the Work available on an Open Access basis (and intends to pay the relevant APC), the terms of reuse of such Open Access shall be governed by a Creative Commons licence – details of these licences and which Creative Commons licence will apply to this Work are set out in our licence referred to above.

Other than as permitted in any relevant BMJ Author's Self Archiving Policies, I confirm this Work has not been accepted for publication elsewhere, is not being considered for publication elsewhere and does not duplicate material already published. I confirm all authors consent to publication of this Work and authorise the granting of this licence.

For peer review only

Symptoms and signs of lung cancer prior to diagnosis: Case-control study using electronic health records from ambulatory care within a large US-based tertiary care center

Maria G. Prado 1

Larry G. Kessler 3

Margaret A Au 1

Hannah Burkhardt 2

Monica Zigman Suchsland 1

Lesleigh Kowalski 1

Kari A. Stephens 1

Meliha Yetisgen 2

Fiona M. Walter 5,6

Richard D Neal 7

Kevin Lybarger 11

Caroline Thompson 8, 9

Morhaf Al Achkar 1

Elizabeth A. Sarma 10

Grace Turner 2

Farhood Farjah 4

Matthew Thompson 1

Affiliations

1 Department of Family Medicine, University of Washington, Seattle, WA, USA

2 Department of Biomedical Informatics and Medical Education, University of Washington, Seattle, WA, USA

For peer review only

3 Department of Health Systems and Population Health, School of Public Health, University of Washington, Seattle, WA, USA

4 Department of Surgery, University of Washington, Seattle, WA, USA

5 Wolfson Institute of Population Health, Barts and The London School of Medicine and Dentistry, Queen Mary University of London, UK

6 The Primary Care Unit, Department of Public Health and Primary Care, University of Cambridge, UK

7 University of Exeter Medical School, University of Exeter, Exeter, UK

8 Department of Epidemiology, Gillings School of Global Public Health, The University of North Carolina at Chapel Hill, Chapel Hill, NC, USA

9 Division of Epidemiology and Biostatistics, School of Public Health, San Diego State University, San Diego, CA, USA

10 Healthcare Delivery Research Program, Division of Cancer Control and Population Sciences, National Cancer Institute, Bethesda, MD, USA

11 Department of Information Sciences and Technology, George Mason University, Fairfax, VA, USA

Corresponding author: Matthew Thompson.

mjt@uw.edu

University of Washington, Box 354696

4225 Roosevelt NE, Suite 308, Seattle, WA 98105

Tel #: (206) 616-8149

For peer review only

Abstract

Objective: Lung cancer is the most common cause of cancer-related death in the United States (US). While most patients are diagnosed following symptomatic presentation, no studies have compared symptoms and physical examination signs at or prior to diagnosis from electronic health records (EHR) in the US. We aimed to identify symptoms and signs in patients prior to diagnosis in EHR data.

Design: Case-control study

Setting: Ambulatory care clinics at a large tertiary care academic health center in the US

Participants, Outcomes: We studied 698 primary lung cancer cases in adults diagnosed between January 1, 2012 and December 31, 2019, and 6,841 controls matched by age, sex, smoking status, and type of clinic. Coded and free-text data from the EHR were extracted from 2 years prior to diagnosis date for cases and index date for controls. Univariate and multivariable conditional logistic regression were used to identify symptoms and signs associated with lung cancer at time of diagnosis, and 1, 3, 6, and 12 months before the diagnosis/index dates.

Results: Eleven symptoms and signs recorded during the study period were associated with a significantly higher chance of being a lung cancer case in multivariable analyses. Of these, seven were significantly associated with lung cancer six months prior to diagnosis: hemoptysis (OR 3.2, 95%CI 1.9-5.3), cough (OR 3.1, 95%CI 2.4-4.0), chest crackles or wheeze (OR 3.1, 95%CI 2.3-4.1), bone pain (OR 2.7, 95%CI 2.1-3.6), back pain (OR 2.5, 95%CI 1.9-3.2), weight loss (OR 2.1, 95%CI 1.5-2.8) and fatigue (OR 1.6, 95%CI 1.3-2.1).

Conclusions: Patients diagnosed with lung cancer appear to have symptoms and signs recorded in the EHR that distinguish them from similar matched patients in ambulatory care, often six months or more before diagnosis. These findings suggest opportunities to improve the diagnostic process for lung cancer.

For peer review only - http://bmjopen.bmj.com/site/about/guidelines.xhtml

Strengths and limitations of this study

Strengths

- Using Natural Language Processing (NLP) techniques to extract symptoms and signs from unstructured data provides a more complete dataset of clinical features presence compared to using coded data alone.
- Case control design recruited cases from ambulatory care population, and controls were randomly selected in a 10:1 ratio based on case clinic type, to reduce the possibility of bias.

Limitations

- Criteria for selection of cases and controls differed slightly; Cases were selected based on a date of the first lung cancer diagnostic code in the EHR, whereas controls were selected based on having a visit to the matched type of clinic type within 3 months of the case diagnosis date.
- Controls were not linked to cancer registry. It is possible that there were a few cases among our controls who had a diagnosis of lung cancer in the cancer registry but no such diagnosis recorded in the EHR at any time (in our time window). Though possible, we believe this highly unlikely. In addition, this lack of linkage to SEER means we were unable to exclude cases of tracheal cancer, mesothelioma, Kaposi sarcoma, lymphoma, or leukemia among controls.
- Availability and timing of symptom data for cases and controls is based on number and frequency of patient interactions with the healthcare system which could be due to a range of factors.

For peer review only

Introduction

Lung cancer is the third most common cancer and the leading cause of cancer death in the United States (US).[1] Most patients with lung cancer are diagnosed following presentation to healthcare settings with symptoms or diagnosed incidentally, and many patients (47%) present with late-stage disease (stages 3 or 4).[2] Screening for lung cancer remains low in the US, with an estimated 6.6% of adults receiving screening in 2019.[3,4] In addition to optimizing screening, early detection efforts have focused on recognition of lung cancer symptoms with an overall goal of identifying patients at earlier, more treatable stages of the disease.[5–7] These symptoms range from ‘alarm’ symptoms, such as hemoptysis (a rare symptom), to relatively non-specific symptoms, such as persistent cough or unexpected weight loss.[6]

Diagnosing lung cancer based on non-specific symptom presentation is challenging, as these symptoms are more commonly associated with benign conditions or may be overlooked for long periods of time. A study of over 43 million patients using Medicare claims data identified a median time from symptom onset to diagnosis of approximately six months.[8] However, claims data lack the granularity needed to identify which clinical features patients present and how these might be used to differentiate patients with lung cancer from the vast majority of patients with benign conditions. To fill this gap, we examined the frequency and association of symptoms and physical examination signs in patients in ambulatory care prior to lung cancer diagnosis and matched controls.

Methods

Study design

We performed a case-control study using data from the University of Washington Medicine (UWM) electronic health records (EHR) and the Seattle/Puget Sound Surveillance, Epidemiology, and End Results (SEER) Program, a National Cancer Institute-supported national cancer registry.[9] This study was approved by the University of Washington Human Subjects Division (STUDY 000013191). A patient and caregiver stakeholder group was involved over a period of 2 years involving regular meetings in the design of this study and in the interpretation of the findings.

For peer review only

Setting

Cases and controls were identified from patients who received ambulatory care at UWM, a large tertiary care academic health center.

Participants

Cases were identified from UWM patients aged 18 years or older, with a first primary lung cancer diagnosis (see International Classification of Diseases (ICD) 9 and 10 codes in Appendix 1) between January 1, 2012 and December 31, 2019, who had an established relationship with a UWM ambulatory care setting in the 2 years before the date of their first recorded lung cancer ICD code in the EHR (EHR diagnosis date). We chose the above study period because of the limited quality of the UWM EHR data prior to 2012. We defined ambulatory care as at least one encounter in family medicine, internal medicine, women's health, obstetrics and gynecology, urgent care, and/or emergency medicine. We used linkage to the regional SEER registry to verify cancer incident cases. Cases were excluded if they did not match with the SEER registry, or if they had a first primary tumor located in anatomy other than the lung, or had evidence of a history of any of the following cancers identified using histology codes in SEER: tracheal cancer, mesothelioma, Kaposi sarcoma, lymphoma, or leukemia. Controls were identified from UWM patients with at least one encounter with the same type of ambulatory clinic within 3 months of the EHR diagnosis date of the index case (matching date). This 3-month window was chosen to avoid potential seasonal differences in respiratory symptoms. For each case, 10 controls were individually matched to the index case by age, sex (male, female), smoking status (ever vs. never), and type of ambulatory care clinic where lung cancer case presented (emergency medicine vs other clinics listed above). We chose a 10:1 control: case match because we recognize the wide variety of patients presenting to ambulatory care settings. Controls were excluded if they had any lung cancer ICD codes in their EHR prior to their matched case diagnosis (index) date. Excluded cancers in cases (based on histology codes from the SEER registry) were not identified in controls as registry data was not available for controls. We also excluded any cases and controls who did not have any ICD codes in any

For peer review only - http://bmjopen.bmj.com/site/about/guidelines.xhtml

encounter in the 2 years prior to diagnosis date (cases) or index date (controls) to ensure availability of data on pre-diagnosis symptoms and signs.

Data Collection

The UWM enterprise-wide data warehouse (EDW) was used to obtain data; this provides a central repository that integrates EHR across the UWM health care system including ambulatory care, specialty care and hospital services. Cases were identified during the study period using ICD codes (Appendix 1) and were linked to SEER to ensure accuracy of case identification and obtain history of previous cancers, histology (for exclusions and lung cancer type), and stage at diagnosis. The date of diagnosis was determined by date of pathology report at UWM. For cases that did not have a diagnosis through pathology or had a discrepancy greater than 30 days between date of pathology and first recorded lung cancer ICD code, two of three clinicians (MT, LKF, MAlA) reviewed the EHR of these cases to adjudicate dates. Controls were randomly sampled from within the matching strata, based on this adjudicated date of diagnosis.

Cases who had undergone lung cancer screening using low-dose computed tomography (LDCT) within the 12 months prior to diagnosis date were identified from billing code (Current Procedural Terminology or CPT 71271) and/or ICD codes (V76.0 [ICD-9] or Z12.2 [ICD-10].

An EHR data extraction protocol was applied to all encounters in the 2-year period prior and up to six months following the diagnosis date (cases) and index date (controls). These data comprised of demographics (e.g., age, sex, race, ethnicity), all ICD codes and CPT procedure codes linked to encounters such as laboratory tests, imaging procedures, and pathology data. We also extracted corresponding unstructured clinical notes for any of the above encounters. ICD codes recorded during the 2-year period prior to diagnosis for cases or prior to index date for controls were searched for the presence of 31 potential comorbidities to calculate the Elixhauser comorbidity index.[10] We excluded lung cancer ICD code information from this calculation. These index scores were then used to calculate van Walraven weighted scores for each patient, a range of -19 to 89.[11,12]

For peer review only - http://bmjopen.bmj.com/site/about/guidelines.xhtml

Symptoms and signs

We identified symptoms and signs using coded data and unstructured data. A list of symptoms and signs which have previously been reported in cohort or case-control studies of individuals with lung cancer were identified from systematic reviews, hand review of individual studies, and from contact with experts in oncology, cardiothoracic surgery, and primary care (FW, RN, FF, MT, see Appendix 2).[5,6,13–18] These were mapped to ICD codes, and used to search the extracted EHR coded data for any encounters that included any of these ICD codes in the 2-year observation period.

Symptoms and signs were automatically extracted from free-text clinical notes using natural language processing (NLP), including notes for all visit types in the 2-year period. In previous work, we developed a deep learning symptom extraction model that generates structured semantic representations of symptoms.[19] The annotation scheme and extraction architecture from this prior work represents symptoms using event-based approach. Each symptom event includes a trigger span that identifies the specific symptom (e.g. “cough” or “shortness of breath”) and multiple attributes that characterize the symptom. The attributes most relevant to this work are the *Assertion* value, which indicates whether the symptom is *present*, *absent*, *possible*, etc., and the *Anatomy*, which indicates the anatomical location of the symptom (e.g. “chest wall” or “lower back”).

Structured symptom predictions were generated using the Span-based Event Extractor architecture in Appendix 3. Each clinical note is split into sentences, which feed into the extractor. The words (tokens) of each sentence are mapped to a vector space using a clinical version of the Bidirectional Encoder Representations from Transformers (BERT) model (no model fine-tuning) [20, 21]. The BERT mapping of each sentence then feeds into a bidirectional Long Short-Term Memory (LSTM) network, which adapts the BERT encoding to the target extraction task. All possible token spans for the sentence are enumerated, and self-attention is used to create a representation for each span, $g_{c,i}$. Each of the enumerated spans is then classified using feedforward neural networks, ϕ_c, that operate on the span representation, $g_{c,i}$. The span scoring layer, ϕ_c, identifies the symptom triggers and attributes. Clinical notes

For peer review only - http://bmjopen.bmj.com/site/about/guidelines.xhtml

frequently describe multiple symptoms within a sentence, and the relationships between the identified symptoms and attributes must be resolved. The identified symptom triggers are paired with the associated symptom attributes through the role scoring layer, ψ_d, which consists of a feedforward neural network that operates on span representation pairs. The output of the Span-based Event Extractor is a structured symptom representation, where identified symptoms are assigned multiple attributes.

In our original symptom work, we trained the Span-based Event Extractor on the COVID-19 Annotated Clinical Text Corpus (CACT).[19] To support the current research, we adapted the symptom extractor to the lung cancer domain. The domain adaptation involved creating the Lung Cancer Annotated Clinical Text (LACT) Corpus, composed of 270 notes from lung cancer patients (170 training and 100 test notes).[22] We trained the lung cancer symptom extractor by combining the CACT and LACT training sets. On the LACT test set, the lung cancer symptom extractor achieved 0.72 F1 for symptom identification and 0.65 F1 for assertion prediction. This extraction performance is comparable to the LACT inter-rater agreement of 0.82 F1 for symptom identification and 0.79 F1 for assertion prediction, indicating the model is achieving approximately human-level performance. We included the extracted symptoms and signs with assertion value present.

Data analysis

Frequencies and counts were calculated for characteristics of cases and controls. The number of symptoms and signs obtained from coded data was compared to that obtained from free-text data using descriptive statistics. The proportion of patients with evidence of each symptom/sign occurring in the 2-year period prior to the diagnosis or index date was described for cases and controls. Odds of patients' case status, based on symptoms and signs identified from a combined dataset of coded and free-text data, were estimated using unadjusted conditional logistic regression. Symptoms and signs associated with lung cancer in unadjusted regressions (p < 0.1) were included into multivariable conditional logistic regression analyses. We used the van Walraven comorbidity score to adjust for population differences in

For peer review only - http://bmjopen.bmj.com/site/about/guidelines.xhtml

comorbidity burden. Analyses were repeated excluding symptom and sign data from 1, 3, 6, and 12 months before the diagnosis (or index) date. Lag times were chosen to provide information on the pattern of symptom-related visits over time and identify the symptoms and signs presenting furthest from diagnosis. We conducted secondary analyses investigating the potential effect of chronic respiratory disease (CRD) status, as defined by the presence of ICD codes within the Elixhauser chronic respiratory disease subgroup, on presence of symptoms and signs in the pre-diagnostic interval. We expected patients with CRD to present with symptoms and signs similar to those that present in early lung cancer. We assessed the effect of CRD by repeating the conditional logistic regression model including CRD as a covariate.

Statistical analyses were conducted using Python 3.7 with the packages SciPy (version 1.4.1) and Statsmodels (version 0.11.1). The study was reported in line with the STROBE guidelines.[23]

Patient and public involvement

We established a technical expert panel (TEP) that included patients with lung cancer and caregivers of patients with lung cancer. The TEP reflected on their personal experience with lung cancer symptoms as well as the lung cancer symptoms we identified in the EHR. They discussed and advised on study methods, data analysis, and communication and visualization of results.

Results

Participants

Selection of cases & controls

A total of 7,883 patients with lung cancer ICD codes were identified in the UWM EDW over the study period (Figure 1). Following linkage of these patients and those identified as having a primary lung tumor from SEER, 4,115 patients were identified common to both, including 741 cases. After matching 7,410 controls, a chart review resulted in exclusion of 43 additional cases.

For peer review only - http://bmjopen.bmj.com/site/about/guidelines.xhtml
For peer review only

Controls that were matched to these 43 cases were excluded (n = 422), resulting in 698 cases matched to 6,841 controls.

Description of cases and controls

Cases and controls were similar in terms of sex and race (cases 50.6% male, 75.5% White; controls 50.5% male, 75.7% White, see Table 1), as well as ethnicity (cases 3.3% Hispanic, controls 3.6%). Cases had higher comorbidity scores (*M* = 14.9, *SD* = 11.6) than controls (*M* = 4.4, *SD* = 8.6). Cases also had a greater median number of health care visits over the 2-year period prior to diagnosis (51.0, 95%CI: 28.0-97.8) than controls (23.0, 95%CI: 9.0-53.0). The difference in median number of health care visits was greater in the last 3-month period prior to the diagnosis/index date (cases 21.0, 95%CI: 12.0-35.0 vs. controls 5.0, 95%CI: 2.0-11.0) than in the 2nd, 3rd, or 4th quarters prior to diagnosis. The stage distribution of cases was as follows: Stage 1- 29%, Stage 2- 7%, Stage 3- 17%, and Stage 4 -42% (5% were Stage 0 or Unknown Stage).

Table 1. Characteristics of patients with lung cancer (cases) and matched controls in ambulatory care

Characteristic	Cases (n=698)	Controls (n=6841)
**Age, years**		
<60	161 (23.1%)	1479 (21.6%)
60-69	257 (36.8%)	2514 (36.7%)
70-79	183 (26.2%)	1865 (27.3%)
80+	97 (13.9%)	983 (14.4%)
**Race**		
American Indian or Alaska Native	6 (0.9%)	78 (1.1%)
Asian	76 (10.9%)	535 (7.8%)
Black or African American	69 (9.9%)	525 (7.7%)
Multiple races	5 (0.7%)	44 (0.6%)
Native Hawaiian or Other Pacific Islander	4 (0.6%)	40 (0.6%)
Unknown	11 (1.6%)	442 (6.5%)

For peer review only - http://bmjopen.bmj.com/site/about/guidelines.xhtml

White	527 (75.5%)	5177 (75.7%)
**Ethnicity**		
Hispanic or Latino	23 (3.3%)	244 (3.6%)
Not Hispanic or Latino	630 (90.3%)	5782 (84.5%)
Unknown	45 (6.4%)	815 (11.9%)
**Sex**		
Male	353 (50.6%)	3452 (50.5%)
**Comorbidity - Elixhauser van Walraven weighted Score, mean (SD)**	14.9 (11.6)	4.4 (8.6)
**Number of clinic visits per patient, median (IQR)**		
In entire data window prior to diagnosis/index	51.0 (28.0 - 97.8)	23.0 (9.0 - 53.0)
In 1st quarter prior to diagnosis/index	21.0 (12.0 - 35.0)	5.0 (2.0 - 11.0)
In 2nd quarter prior to diagnosis/index	7.0 (3.0 - 14.0)	5.0 (2.0 - 11.0)
In 3rd quarter prior to diagnosis/index	7.0 (3.0 - 12.0)	5.0 (2.0 - 11.0)
In 4th quarter prior to diagnosis/index	6.0 (3.0 - 13.0)	5.0 (2.0 - 11.0)

Frequency of symptoms and signs extracted from coded and free-text data

Of the 22 symptoms and signs that we systematically examined, NLP identified 20 of the 22 symptoms and signs in greater proportions of patients affected than from the coded data alone (see Appendix 4). In comparison to coded data, we saw a range of 12.9% to 97.6% greater symptom and signs reports with NLP of textual clinical notes. In contrast, a greater proportion of patients had two symptoms and signs (shoulder pain, lymphadenopathy) identified from coded rather than free-text data.

Comparison of frequency of symptoms and signs between cases and controls

The frequency of all 22 symptoms and signs examined was higher in cases than controls (see Table 2). Moreover, the ranking of symptoms and signs differed slightly between cases and controls, with cases reporting cough (82.1%), shortness of breath (73.8%), fatigue (68.2%), ankle swelling (64.0%), and chest pain (57.7%), whereas controls reported ankle swelling (26.9%), cough (24.2%), shortness of breath (23.6%), fatigue (23.2%) and chest pain (20.5%)

For peer review only - http://bmjopen.bmj.com/site/about/guidelines.xhtml

most frequently. Hemoptysis occurred relatively infrequently among cases (16.5%) and rarely among controls (1.0%).

Table 2. Comparison of frequency of symptoms and signs identified in coded or free-text data in cases compared to controls

Symptom or sign	Cases (n=698)	Controls (n=6841)
Cough	573 (82.1%)	1654 (24.2%)
Shortness of breath	515 (73.8%)	1613 (23.6%)
Fatigue	476 (68.2%)	1587 (23.2%)
Ankle swelling	447 (64.0%)	1838 (26.9%)
Chest Pain	403 (57.7%)	1401 (20.5%)
Chest crackles or wheeze	397 (56.9%)	575 (8.4%)
Back pain	350 (50.1%)	946 (13.8%)
Change in bowel habits	336 (48.1%)	1155 (16.9%)
Muscle weakness	334 (47.9%)	1102 (16.1%)
Fever	322 (46.1%)	1334 (19.5%)
Weight loss	308 (44.1%)	522 (7.6%)
Headache	304 (43.6%)	1205 (17.6%)
Dizziness	299 (42.8%)	1319 (19.3%)
Bone pain	270 (38.7%)	725 (10.6%)
Lack of appetite	196 (28.1%)	457 (6.7%)
Shoulder pain	180 (25.8%)	713 (10.4%)
Lymphadenopathy	151 (21.6%)	105 (1.5%)
Night sweats	150 (21.5%)	371 (5.4%)
Changes in sleep	134 (19.2%)	631 (9.2%)
Hemoptysis	115 (16.5%)	67 (1.0%)
Hoarseness	67 (9.6%)	133 (1.9%)
Finger clubbing	39 (5.6%)	2 (0.0%)

Univariate associations of symptoms and signs between cases and controls

In models adjusted for comorbidity score, when considered independently, all 22 symptoms and signs had odds ratios that were significantly different between cases and controls (all $p < 0.0001$, see Table 3). The symptoms and signs with the largest odds ratios (OR) significantly associated with a higher chance of being a case were finger clubbing (OR 175.7, 95%CI: 40.1-770.0), hemoptysis (OR 14.5, 95%CI: 10.2-20.8), cough (OR 11.1, 95%CI: 8.8-13.9), chest crackles or wheeze (OR 9.9, 95%CI: 8.1-12.2), and lymphadenopathy (OR 9.4, 95%CI: 6.9-12.8).

For peer review only

Table 3. Univariate and multivariate analyses of symptoms and signs identified in coded or free-text data of cases compared to controls, adjusted for comorbidity (descending order by multivariate odds ratios)

Symptom or sign	Univariate Odds ratio (95%CI)	Multivariate Odds ratio (95%CI)	Multivariate P value
Finger clubbing	175.7 (40.1 - 770.0)*	50.1 (8.9 - 283.3)	<0.0001
Lymphadenopathy	9.4 (6.9 - 12.8)*	5.8 (3.8 - 8.8)	<0.0001
Cough	11.1 (8.8 - 13.9)*	4.7 (3.5 - 6.3)	<0.0001
Hemoptysis	14.5 (10.2 - 20.8)*	3.5 (2.2 - 5.5)	<0.0001
Chest crackles or wheeze	9.9 (8.1 - 12.2)*	3.2 (2.4 - 4.3)	<0.0001
Weight loss	5.9 (4.8 - 7.2)*	2.9 (2.2 - 3.9)	<0.0001
Back pain	4.7 (3.9 - 5.7)*	2.4 (1.8 - 3.1)	<0.0001
Bone pain	4.6 (3.8 - 5.7)*	2.3 (1.7 - 3.1)	<0.0001
Shortness of breath	6.0 (4.9 - 7.3)*	1.9 (1.4 - 2.5)	<0.0001
Fatigue	4.8 (4.0 - 5.8)*	1.8 (1.4 - 2.4)	<0.0001
Chest Pain	3.6 (3.0 - 4.3)*	1.4 (1.1 - 1.8)	0.0118
Shoulder pain	2.3 (1.8 - 2.8)*	1.3 (1.0 - 1.7)	0.1111
Ankle swelling	3.3 (2.7 - 4.0)*	1.1 (0.9 - 1.5)	0.3643
Headache	2.5 (2.1 - 3.0)*	1.1 (0.8 - 1.4)	0.5619
Hoarseness	3.5 (2.5 - 5.0)*	1.1 (0.7 - 1.7)	0.8447
Change in bowel habits	3.0 (2.5 - 3.6)*	1.0 (0.8 - 1.4)	0.8880
Muscle weakness	2.9 (2.4 - 3.5)*	1.0 (0.7 - 1.3)	0.9581
Night sweats	3.3 (2.6 - 4.2)*	0.8 (0.6 - 1.2)	0.2998
Lack of appetite	2.6 (2.1 - 3.3)*	0.7 (0.5 - 0.9)	0.0193
Dizziness	2.0 (1.7 - 2.4)*	0.6 (0.4 - 0.8)	0.0004
Changes in sleep	1.3 (1.1 - 1.7)*	0.5 (0.3 - 0.6)	<0.0001
Fever	2.1 (1.7 - 2.5)*	0.4 (0.3 - 0.6)	<0.0001

Note: Conditional logistic regression models adjusted for comorbidities using van Walraven weighted score with each symptom or sign modeled individually (univariate) and mutually adjusted (multivariate)
*Significant at p<0.0001 for univariate analysis

Multivariable associations of symptoms and signs between cases and controls

We included all 22 symptoms and signs from the univariate analysis and comorbidity score in a multivariable analysis. After mutual adjustment, 15 had significant ORs (all $p < 0.05$, see Table 3). The presence of 11 symptoms and signs were associated with a significantly higher odds of being a case, with ORs ranging from 1.4 (chest pain) to 50.1 (finger clubbing). The largest ORs were noted for finger clubbing (OR 50.1, 95%CI: 8.9-283.3), lymphadenopathy (OR 5.8, 95%CI: 3.8-8.8), cough (OR 4.7, 95%CI: 3.5-6.3), hemoptysis (OR 3.5, 95%CI: 2.2-5.5) and chest crackles or wheeze (OR 3.2, 95%CI: 2.4-4.3). In contrast, the presence of four symptoms was associated with a significantly higher odds of being a control: fever (OR 0.4, 95%CI: 0.3-0.6), changes in

For peer review only - http://bmjopen.bmj.com/site/about/guidelines.xhtml

sleep (OR 0.5, 95%CI: 0.3-0.6), dizziness (OR 0.6, 95%CI: 0.4-0.8), and lack of appetite (OR 0.7, 95%CI: 0.5-0.9).

We repeated the multivariable analysis, excluding symptoms and signs recorded in periods of 1, 3, 6 and 12 months prior to diagnosis (see Figure 2). Some symptoms and signs remained significantly associated with cases up to 6 months prior to diagnosis (cough, hemoptysis, chest crackles and wheeze, weight loss, back pain, bone pain, fatigue). Of these, all except weight loss were also significantly associated with cases 12 months prior to diagnosis. Other symptoms and signs became significantly associated with being a case closer to the date of diagnosis: shortness of breath and chest pain (3 months prior to diagnosis), lymphadenopathy and finger clubbing (1 month prior) (see Appendix 5).

Secondary analyses

To determine whether the associations were robust to the presence of CRD, we performed a secondary conditional logistic regression that was adjusted for CRD, along with all our matching variables and comorbidity score. The presence of CRD appeared to have no statistically significant effect when directly added as a covariate (OR: 1.05, 95%CI: (0.81, 1.36, p = 0.7229, see Appendices 6 & 7).

Discussion

Main findings

This is the first case-control study in the US to use routine, prospectively collected EHR data to describe the frequency of symptoms and signs of lung cancer and estimate associations with incident lung cancer cases compared to non-lung cancer patients receiving routine ambulatory care in the same time period. Our findings provide unique information on symptoms and signs associated with a higher chance of a patient in ambulatory care being diagnosed with lung cancer, and the duration of these associations prior to their cancer diagnosis. In contrast to prior work on national databases, extracting clinicians' documentation of clinical features from their free text clinical notes using NLP provided more complete symptom identification data, rather than relying on data available only in coded, structured data collected in routine care. Our findings provide evidence-based, quantitative support for the development of decision

For peer review only - http://bmjopen.bmj.com/site/about/guidelines.xhtml

rules around the diagnostic workup of symptomatic patients, which could lead to the improvement of earlier diagnosis of lung cancer. Of the 22 symptoms and signs studied, 11 were found in adjusted models to be associated with a higher chance of being a lung cancer case, and most of these 11 were present and still significantly associated up to 12 months prior to diagnosis; this suggests opportunities for improved screening practices that may lead to earlier diagnosis and possibly improved outcomes.

Our findings also suggest that the clinical presentation of lung cancer appears to be similar, regardless of the presence of other comorbidities, CRD, or smoking. For patients and clinicians this is important as several of the symptoms or signs we identified may currently be dismissed as being attributable to underlying smoking or comorbid conditions.

Comparison with existing literature

Several of the symptoms and signs we found as having statistically significant odds ratios have been identified in studies using data from ambulatory care in other healthcare systems, especially hemoptysis and cough. However, among the symptoms and signs Hamilton and colleagues (2005) found to be associated with being a lung cancer case in the United Kingdom (UK), loss of appetite had the highest OR (86.0), whereas we failed to identify an association with lung cancer.[5] This may be due to a difference in study populations or our use of NLP in EHR data.

Our findings also provide evidence of the temporality of a 'clinical signal' for lung cancer based on symptoms and signs documented in the EHR, at least six and up to 12 months prior to diagnosis, consistent with a Medicare claims study. Data from our study and Nadpara and colleagues' (2015) study, which used claims data, provide evidence for time intervals from first presentation with symptoms to diagnosis that are on the upper range (six months) of those reported using analysis of coded symptoms in primary care databases in several UK and European studies.[8] These describe the overall time interval from first symptom recording in medical records to diagnosis ranging from 3- to 6-months.[6,24,25] While not directly comparable,

For peer review only - http://bmjopen.bmj.com/site/about/guidelines.xhtml

qualitative research from patients with lung cancer and caregivers describe changes noticeable to the individual more than 12 months before attending a health care visit.[17,26,27]

Strengths and limitations

Using NLP to extract symptoms and signs from unstructured data allowed us to capture a more complete dataset of symptom presence compared to using coded data alone. We selected cases from an empaneled ambulatory care population, where we expected EHR data would be available for the period of interest in this study and attempted to exclude patients who were attending only for secondary or tertiary care provided at UWM. Controls were randomly selected based on case clinic type, to reduce the possibility of bias, and duration of follow-up time and availability of data for cases and controls were similar, particularly in visit frequency. We used a robust design where we matched 10 controls to 1 case, providing greater power and precision, and matched on smoking so that our analyses could not be confounded based on ever vs. never exposure to smoking.

Limitations included criteria for selection of cases and controls differed slightly. As is customary in incident case-control studies, cases were selected based on a diagnosis date defined as the date of the first lung cancer ICD code in the EHR. In this way, we captured the diagnostic path from symptom presentation to diagnosis for all cases. Controls were selected based on having a visit to the matched case clinic type (to account for difference in emergency vs other forms of ambulatory care) within 3 months of the case diagnosis date, however the timing of control selection does not necessarily reflect a “pathway to diagnosis” for some other condition, just recent routine care. Additionally, because we did not link to SEER for the control population, we were unable to apply two of the case exclusion criteria to our control sample: 1) no current or prior history of lung cancer in SEER, although we did check the UW EHR for concurrent lung-cancer related ICD codes and medical history so this should be rare, and 2) no prior history of tracheal cancer, mesothelioma, Kaposi sarcoma, lymphoma, or leukemia in SEER. Additionally, EHR data can sometimes be subject to misclassification. For example, detailed EHR smoking history may be unreliable and the EHR does not reliably capture health literacy or

For peer review only - http://bmjopen.bmj.com/site/about/guidelines.xhtml

socioeconomic status; however, we used a very broad definition of smoking (ever vs. never) and used a comorbidity score to control for health status. Finally, availability and timing of symptom data for cases and controls is based on patient interactions with the healthcare system, not a pre-specified protocol of data collection. Patients who have more contact with their providers (which could be due to a range of factors) may have had more data captured.

Implications for clinicians, researchers, policy makers

Differentiating patients who may have symptoms or signs of lung cancer from those attending ambulatory care is a critical and challenging step in the earlier detection of this cancer. Our findings not only identify the 'red flag' (highly specific, but infrequent) symptoms and signs that primary care providers should be aware of (e.g., hemoptysis), but also highlight which of a larger range of 'non-specific' symptoms and signs should equally raise suspicion such as bone pain and weight loss. Furthermore, our findings support the importance of clinical documentation, and continuity of care to identify and act on sustained changes in patients' clinical presentations.

Confirmation of our findings using datasets from other healthcare systems in the U.S. are needed and could be enhanced by more advanced machine learning modelling to incorporate additional clinical variable including quantitative data such as changes in body weight or results of routinely collected laboratory tests, given emerging evidence for associations between weight loss and minor deviations of hemoglobin or platelet count with incident cancer.[28] Given the low uptake of low dose CT screening for lung cancer in the U.S., our findings provide support for revising current priorities to improve early diagnosis of lung cancer.[29]

Conclusions

Patients in ambulatory care settings who are subsequently diagnosed with lung cancer appear to have symptoms and signs that distinguish them from other patients, often months before lung cancer diagnosis. To improve earlier detection of lung cancer, interventions are urgently

For peer review only - http://bmjopen.bmj.com/site/about/guidelines.xhtml

needed that promote earlier screening based on symptomatic presentations in ambulatory care that may lead to an earlier detection and treatment of lung cancer.

Author Contributions: MGP extracted data from UW Medicine and linked to SEER Cancer Registry, supported study management and execution, wrote the manuscript, provided critical comments, edited the manuscript, and approved its final version. LGK assisted with design of the study and supported its execution, provided advice and expertise for study design, analyses and interpretation of data, wrote the manuscript, provided critical comments, edited the manuscript, and approved its final version. MAA performed the analyses, provided advice and expertise for study design, conducted analyses and interpretation of data, provided critical comments, edited the manuscript, and approved its final version. HB supported data extraction and data linkage, assisted with analyses, created figures and tables, assisted with interpretation of data, provided critical comments, edited the manuscript, and approved its final version. MZS assisted with design of the study and supported its execution, extracted data from UW Medicine and linked to SEER Cancer Registry, provided further advice and expertise for study design, and interpretation of data, provided critical comments, edited the manuscript, and approved its final version. LK assisted with design of the study and supported its execution, provided advice and expertise for study design, clinical interpretation of data, provided critical comments, edited the manuscript, and approved its final version. KAS assisted with design of the study, extracted data from UW Medicine and linked to SEER Cancer Registry, provided advice and expertise for study design, interpretation of data, provided critical comments, edited the manuscript, and approved its final version. MY created the natural language annotation tool and extracted free text data, assisted with interpretation of data, provided critical comments, edited the manuscript, and approved its final version. FMW provided advice and expertise for study design, clinical input and interpretation of data, provided critical comments, edited the manuscript, and approved its final version. RDN provided advice and expertise for study design, clinical input and interpretation of data, provided critical comments, edited the manuscript, and approved its final version. KL created the natural language

For peer review only

annotation tool and extracted free text data, assisted with interpretation of data, provided critical comments, edited the manuscript, and approved its final version. CT provided advice and expertise for study design, analytic methods and interpretation of data, provided critical comments, edited the manuscript, and approved its final version. MAlA provided advice and expertise for study design, clinical input and interpretation of data, provided critical comments, edited the manuscript, and approved its final version. EAS provided advice and expertise for study design, clinical input and interpretation of data, provided critical comments, edited the manuscript, and approved its final version. GT supported implementation of the natural language annotation tool and extracted free text data, assisted with interpretation of data, provided critical comments, edited the manuscript, and approved its final version. FF provided advice and expertise for study design, clinical input and interpretation of data, provided critical comments, edited the manuscript, and approved its final version. MT was the Principal Investigator for the study and is its guarantor, designed the study and supervised its execution, provided clinical guidance, interpreted data, wrote the manuscript, edited the manuscript, and approved its final version.

Funding: This research was funded by the Gordon and Betty Moore Foundation through Grant GBMF8837 to the University of Washington. This research is linked to the CanTest Collaborative, which is funded by Cancer Research UK [C8640/A23385], of which FMW is Director and RDN and MT are Associate Directors. This research was supported by the Cancer Surveillance System of the Fred Hutchinson Cancer Research Center, which is funded by Contract Numbers HHSN261201800004I & N01 PC-2018-00004 from the Surveillance, Epidemiology and End Results (SEER) Program of the National Cancer Institute with additional support from the Fred Hutchinson Cancer Research Center and the State of Washington.

The views expressed are those of the authors and do not necessarily represent the official position of the National Cancer Institute, the National Institute of Health, or Department of Health and Human Services.

For peer review only

Informed Consent Statement: Not applicable.

Data Sharing Statement: Fully anonymized data may be available on reasonable request to the corresponding author, once appropriate data sharing and ethics approvals have been obtained.

Acknowledgments: We would like to thank the patients and clinicians at University of Washington Medicine, and the members of our TEP.

Conflicts of Interest: The authors declare no conflict of interest.

Ethical approval statement: The study was conducted according to the guidelines of the Declaration of Helsinki, and was classified as Exempt by the University of Washington Human Subjects Division.

Figure legends

Figure 1. Flow chart of case and control selection

Figure 2: Multivariable analysis of symptoms or signs of cases compared to controls with symptom and sign data excluded from 1, 3, 6, and 12 months prior to diagnosis/index date

References

1. Centers for Diseases Control and Prevention. Leading cancer cases and deaths, all Races/Ethnicities, male and female, 2018. Accessed January 16, 2022. https://gis.cdc.gov/grasp/USCS/DataViz.html

2. American Lung Association. State of Lung Cancer 2020 Report. Published online 2020:15.

3. Fedewa SA, Bandi P, Smith RA, Silvestri GA, Jemal A. Lung Cancer Screening Rates During the COVID-19 Pandemic. *Chest*. Published online July 2021:S0012369221013647. doi:10.1016/j.chest.2021.07.030

4. The National Lung Screening Trial Research Team. Reduced Lung-Cancer Mortality with Low-Dose Computed Tomographic Screening. *N Engl J Med*. 2011;365(5):395-409. doi:10.1056/NEJMoa1102873

5. Hamilton W. What are the clinical features of lung cancer before the diagnosis is made? A population based case-control study. *Thorax*. 2005;60(12):1059-1065. doi:10.1136/thx.2005.045880

For peer review only - http://bmjopen.bmj.com/site/about/guidelines.xhtml

6. Walter FM, Rubin G, Bankhead C, et al. Symptoms and other factors associated with time to diagnosis and stage of lung cancer: a prospective cohort study. *Br J Cancer*. 2015;112(S1):S6-S13. doi:10.1038/bjc.2015.30

7. Koo MM, Hamilton W, Walter FM, Rubin GP, Lyratzopoulos G. Symptom Signatures and Diagnostic Timeliness in Cancer Patients: A Review of Current Evidence. *Neoplasia*. 2018;20(2):165-174. doi:10.1016/j.neo.2017.11.005

8. Nadpara PA, Madhavan SS, Tworek C, Sambamoorthi U, Hendryx M, Almubarak M. Guideline-concordant lung cancer care and associated health outcomes among elderly patients in the United States. *J Geriatr Oncol*. 2015;6(2):101-110. doi:10.1016/j.jgo.2015.01.001

9. Cancer Statistics Review, 1975-2018 - SEER Statistics. Accessed January 16, 2022. https://seer.cancer.gov/csr/1975_2018/

10. Elixhauser A, Steiner C, Harris DR, Coffey RM. Comorbidity Measures for Use with Administrative Data: *Med Care*. 1998;36(1):8-27. doi:10.1097/00005650-199801000-00004

11. van Walraven C, Austin PC, Jennings A, Quan H, Forster AJ. A Modification of the Elixhauser Comorbidity Measures Into a Point System for Hospital Death Using Administrative Data. *Med Care*. 2009;47(6):626-633. doi:10.1097/MLR.0b013e31819432e5

12. Thompson NR, Fan Y, Dalton JE, et al. A New Elixhauser-based Comorbidity Summary Measure to Predict In-Hospital Mortality. *Med Care*. 2015;53(4):374-379. doi:10.1097/MLR.0000000000000326

13. Hippisley-Cox J, Coupland C. Symptoms and risk factors to identify men with suspected cancer in primary care: derivation and validation of an algorithm. *Br J Gen Pract*. 2013;63(606):e1-e10. doi:10.3399/bjgp13X660724

14. Gould MK, Ghaus SJ, Olsson JK, Schultz EM. Timeliness of Care in Veterans With Non-small Cell Lung Cancer. *Chest*. 2008;133(5):1167-1173. doi:10.1378/chest.07-2654

15. Ades AE, Biswas M, Welton NJ, Hamilton W. Symptom lead time distribution in lung cancer: natural history and prospects for early diagnosis. *Int J Epidemiol*. 2014;43(6):1865-1873. doi:10.1093/ije/dyu174

16. Redaniel MT, Martin RM, Ridd MJ, Wade J, Jeffreys M. Diagnostic Intervals and Its Association with Breast, Prostate, Lung and Colorectal Cancer Survival in England: Historical Cohort Study Using the Clinical Practice Research Datalink. Metze K, ed. *PLOS ONE*. 2015;10(5):e0126608. doi:10.1371/journal.pone.0126608

17. Corner J, Hopkinson J, Fitzsimmons D, Barclay S, Muers M. Is late diagnosis of lung cancer inevitable? Interview study of patients' recollections of symptoms before diagnosis. *Thorax*. 2005;60(4):314-319. doi:10.1136/thx.2004.029264

For peer review only - http://bmjopen.bmj.com/site/about/guidelines.xhtml

18. Tod AM, Craven J, Allmark P. Diagnostic delay in lung cancer: a qualitative study: Diagnostic delay in lung cancer. *J Adv Nurs*. 2008;61(3):336-343. doi:10.1111/j.1365-2648.2007.04542.x

19. Lybarger K, Ostendorf M, Thompson M, Yetisgen M. Extracting COVID-19 diagnoses and symptoms from clinical text: A new annotated corpus and neural event extraction framework. *J Biomed Inform*. 2021;117:103761. doi:10.1016/j.jbi.2021.103761

20. Devlin J, Chang M, Lee K, Toutanova K, BERT: Pre-training of deep bidirectional transformers for language understanding, in: *North American Chapter of the Association for Computational Linguistics*, 2019, pp. 4171–4186. doi:10:18653/v1/N19-1423.

21. Alsentzer E, Murphy J, Boag W, et al. Publicly available clinical BERT embeddings, in: *Clinical Natural Language Processing Workshop*, 2019, pp. 72–78. doi:10:18653/v1/W19-1909.

22. Turner G, Chang J, Dorvall N, et al. Domain Adaptation of a Deep Learning Symptom Extractor for Different Patient Populations and Clinical Settings. In: *AMIA 2022 Informatics Summit*.

23. von Elm E, Altman DG, Egger M, Pocock SJ, Gøtzsche PC, Vandenbroucke JP. The Strengthening the Reporting of Observational Studies in Epidemiology (STROBE) Statement: Guidelines for reporting observational studies. *Int J Surg*. 2014;12(12):1495-1499. doi:10.1016/j.ijsu.2014.07.013

24. Ellis PM, Vandermeer R. Delays in the diagnosis of lung cancer. *J Thorac Dis*. 2011;3(3):183-188. doi:10.3978/j.issn.2072-1439.2011.01.01

25. Koyi H, Hillerdal G, Brandén E. Patient's and doctors' delays in the diagnosis of chest tumors. *Lung Cancer*. 2002;35(1):53-57. doi:10.1016/S0169-5002(01)00293-8

26. Al Achkar M, Zigman Suchsland M, Walter FM, Neal RD, Goulart BHL, Thompson MJ. Experiences along the diagnostic pathway for patients with advanced lung cancer in the USA: a qualitative study. *BMJ Open*. 2021;11(4):e045056. doi:10.1136/bmjopen-2020-045056

27. Corner J, Hopkinson J, Roffe L. Experience of health changes and reasons for delay in seeking care: a UK study of the months prior to the diagnosis of lung cancer. *Soc Sci Med 1982*. 2006;62(6):1381-1391. doi:10.1016/j.socscimed.2005.08.012

28. Nicholson BD, Aveyard P, Koshiaris C, et al. Combining simple blood tests to identify primary care patients with unexpected weight loss for cancer investigation: Clinical risk score development, internal validation, and net benefit analysis. *PLOS Med*. 2021;18(8):e1003728. doi:10.1371/journal.pmed.1003728

29. Sarma EA, Kobrin SC, Thompson MJ. A Proposal to Improve the Early Diagnosis of Symptomatic Cancers in the United States. *Cancer Prev Res (Phila Pa)*. 2020;13(9):715-720. doi:10.1158/1940-6207.CAPR-20-0115

For peer review only

For peer review only - http://bmjopen.bmj.com/site/about/guidelines.xhtml

For peer review only

For peer review only - http://bmjopen.bmj.com/site/about/guidelines.xhtml

Figure 1. Flow chart of case and control selection

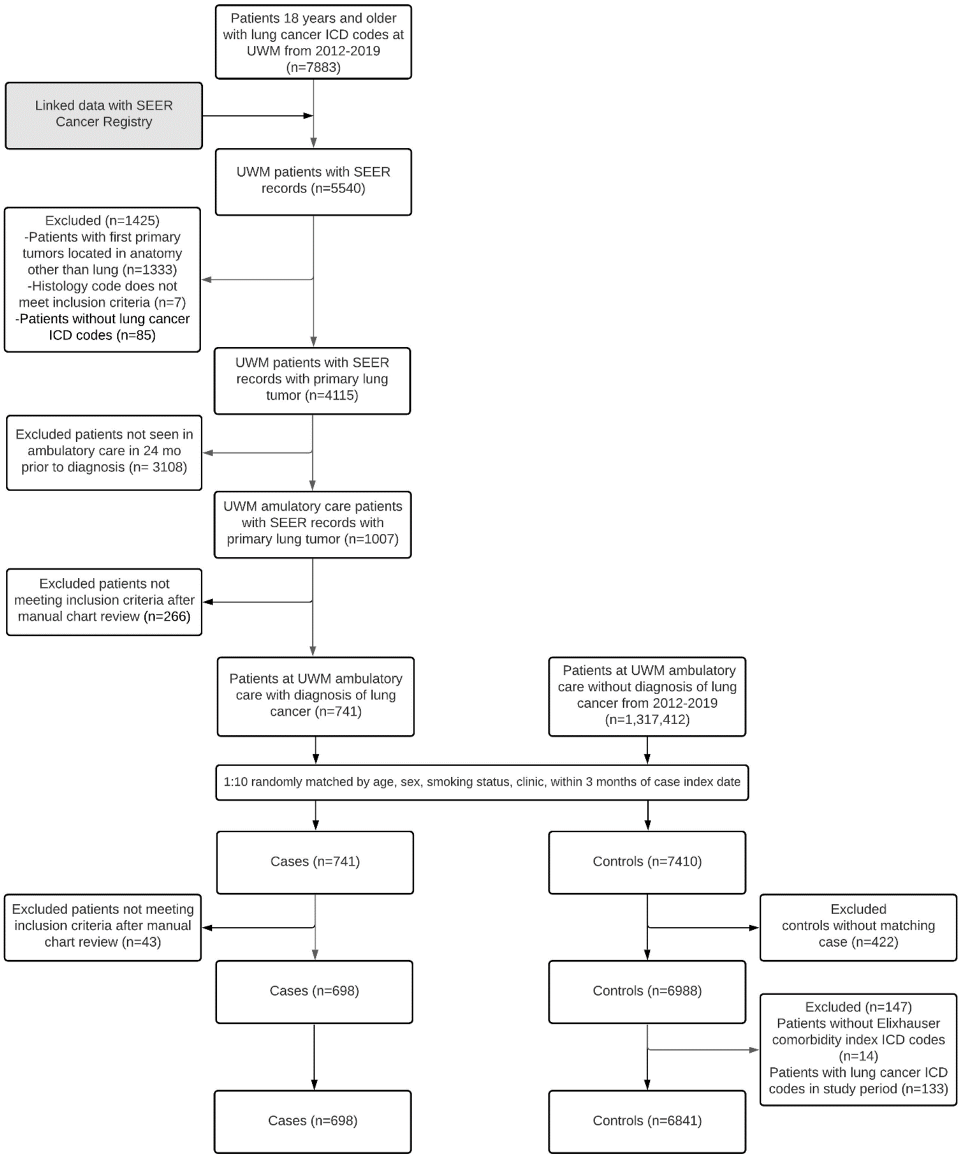

Figure 2: Multivariable analysis of symptoms or signs of cases compared to controls with symptom and sign data excluded from 1, 3, 6, and 12 months prior to diagnosis/index date

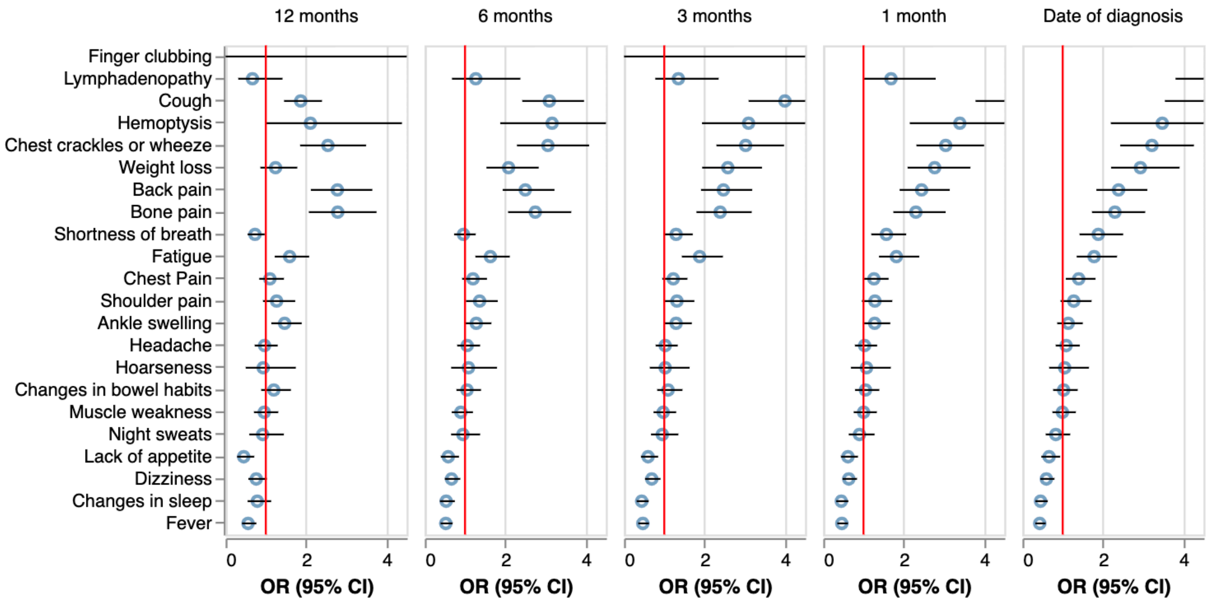

Note: Mutual adjustment of all symptoms and signs in using a conditional logistic regression model stratified by time prior to date of diagnosis. Models additionally adjusted for comorbidities using van Walraven weighted score. For the complete set of results, see Appendix 5.

For peer review only

Symptoms and signs of lung cancer prior to diagnosis: Comparative study using natural language processing of electronic health records

Appendix 1. Diagnostic codes used to identify cases of lung cancer

ICD 9: 162.2 – 162.9

- 162.2 - Malignant neoplasm of main bronchus
- 162.3 - Malignant neoplasm of upper lobe, bronchus or lung
- 162.4 - Malignant neoplasm of middle lobe, bronchus or lung
- 162.5 - Malignant neoplasm of lower lobe, bronchus or lung
- 162.8 - Malignant neoplasm of other parts of bronchus or lung
- 162.9 - Malignant neoplasm of bronchus and lung, unspecified

ICD 10: C34.0 – C34.9

- C34.0 - Malignant neoplasm of main bronchus
- C34.00 - Malignant neoplasm of unspecified main bronchus
- C34.01 - Malignant neoplasm of right main bronchus
- C34.02 - Malignant neoplasm of left main bronchus
- C34.1 - Malignant neoplasm of upper lobe, bronchus or lung
- C34.10 - Malignant neoplasm of upper lobe, unspecified bronchus or lung
- C34.11 - Malignant neoplasm of upper lobe, right bronchus or lung
- C34.12 - Malignant neoplasm of upper lobe, left bronchus or lung
- C34.2 - Malignant neoplasm of middle lobe, bronchus or lung
- C34.3 - Malignant neoplasm of lower lobe, bronchus or lung
- C34.30 - Malignant neoplasm of lower lobe, unspecified bronchus or lung
- C34.31 - Malignant neoplasm of lower lobe, right bronchus or lung
- C34.32 - Malignant neoplasm of lower lobe, left bronchus or lung
- C34.8 - Malignant neoplasm of overlapping sites of bronchus and lung
- C34.80 - Malignant neoplasm of overlapping sites of unspecified bronchus and lung
- C34.81 - Malignant neoplasm of overlapping sites of right bronchus and lung
- C34.82 - Malignant neoplasm of overlapping sites of left bronchus and lung
- C34.9 - Malignant neoplasm of unspecified part of bronchus or lung
- C34.90 - Malignant neoplasm of unspecified part of unspecified bronchus or lung
- C34.91 - Malignant neoplasm of unspecified part of right bronchus or lung
- C34.92 - Malignant neoplasm of unspecified part of left bronchus or lung

Excluded ICD Diagnostic Codes

- ICD-9: 162.0
- ICD-10: C33

Excluded Histology codes

- Mesothelioma: 9050-9055
- Kaposi Sarcoma: 9140
- Lymphoma/leukemia: M9590-M9992

For peer review only

Appendix 2. Symptoms and signs Identified in peer-reviewed literature previously associated with lung cancer in primary care populations

Symptom or sign	ICD 9 code(s)	ICD10 code(s)	References
Ankle swelling	782.3	R60.9	[1]Ellis (2011)
Back pain	724.1	M54.6	[1]Ellis (2011) [2]Molassiotis (2010)
Bone pain	733.9	M85.80	[3]Gould (2008) [4]Nadpara (2015)
Changes in bowel habits	787.99	R19.4	[5]Corner (2005)
Changes in sleep	780.50	G47.9	[5]Corner (2005)
Chest Pain	786.5<br>786.50<br>786.51<br>786.52<br>786.59	R07.9<br>R07.81	[1]Ellis (2011) [4]Nadpara (2015) [5]Corner (2005) [6]Chowienczyk, Hamilton (2020) [7]Walter (2015) [8]Hamilton (2005) [9]Ades (2014) [10]Redaniel (2015) [11]Tod (2008) [12]Mitchell (2013)
Chest crackles or wheeze	786.7	R09.89	[10]Redaniel (2015)
Cough	786.2<br>491.0	R05	[1]Ellis (2011) [2]Molassiotis (2010) [4]Nadpara (2015) [5]Corner (2005) [6]Chowienczyk, Hamilton (2020) [7]Walter (2015) [9]Ades (2014) [10]Redaniel (2015) [11]Tod (2008) [12]Mitchell (2013) [13]Menon (2019)
Dizziness	780.4	R42	[2]Molassiotis (2010)
Fatigue/tiredness	780.79	R53.81<br>R53.8<br>R53.83<br>R53.1	[1]Ellis (2011) [2]Molassiotis (2010) [5]Corner (2005) [6]Chowienczyk, Hamilton (2020) [7]Walter (2015) [8]Hamilton (2005) [10]Redaniel (2015) [11]Tod (2008) [13]Menon (2019)
Fever	780.6<br>780.60	R50.9	[4]Nadpara (2015)
Finger clubbing	781.5	R68.3	[4]Nadpara (2015) [8]Hamilton (2005) [10]Redaniel (2015)
Headache	784.0	R51	[1]Ellis (2011)
Hemoptysis	786.3<br>786.30<br>786.39	R04.2	[1]Ellis (2011) [4]Nadpara (2015) [5]Corner [6]Chowienczyk, Hamilton (2020) [7]Walter (2015) [8]Hamilton (2005) [10]Redaniel (2015) (2005) [11]Tod (2008) [12]Mitchell (2013) [13]Menon (2019) [14]Hippisley-Cox (2011)

For peer review only

Hoarseness	784.49<br>784.42	R49.8<br>R49.0	[1]Ellis (2011) [2]Molassiotis (2010) [7]Walter (2015) [10]Redaniel (2015) [11]Tod (2008) [12]Mitchell (2013)
Lack of appetite	783	R63.0	[1]Ellis (2011) [2]Molassiotis (2010) [5]Corner (2005) [6]Chowienczyk, Hamilton (2020) [7]Walter (2015) [8]Hamilton (2005) [13]Menon (2019)
Lympadenopathy	785.6	R59.9	[10]Redaniel (2015) [12]Mitchell (2013)
Muscle weakness	728.87	M62.81	[4]Nadpara (2015) [12]Mitchell (2013)
Night sweats	780.8	R61	[3]Gould (2008) [5]Corner (2005)
Shortness of breath	786.05<br>786.0<br>786.9	R06.02<br>R06.00<br>R06.09	[1]Ellis (2011) [2]Molassiotis (2010) [4]Nadpara (2015) [5]Corner (2005) [6]Chowienczyk, Hamilton (2020) [7]Walter (2015) [8]Hamilton (2005) [10]Redaniel (2015) [12]Mitchell (2013) [13]Menon (2019)
Shoulder pain	719.41	M25.511<br>M25.512<br>M25.519	[10]Redaniel (2015) [12]Mitchell (2013)
Weight loss	783.21	R63.4	[1]Ellis (2011) [4]Nadpara (2015) [5]Corner (2005) [6]Chowienczyk, Hamilton (2020) [7]Walter (2015) [8]Hamilton (2005) [10]Redaniel (2015) [11]Tod (2008) [12]Mitchell (2013)
Wheezing and stridor	786.07<br>786.1	R06.2<br>R06.1	[4]Nadpara (2015) [10]Redaniel (2015)

1. Ellis PM, Vandermeer R. Delays in the diagnosis of lung cancer. *J Thorac Dis*. 2011;3(3):183-188. doi:10.3978/j.issn.2072-1439.2011.01.01

2. Molassiotis A, Wilson B, Brunton L, Chandler C. Mapping patients' experiences from initial change in health to cancer diagnosis: a qualitative exploration of patient and system factors mediating this process. *Eur J Cancer Care (Engl)*. 2010;19(1):98-109. doi:10.1111/j.1365-2354.2008.01020.x

3. Gould MK, Ghaus SJ, Olsson JK, Schultz EM. Timeliness of Care in Veterans With Non-small Cell Lung Cancer. *Chest*. 2008;133(5):1167-1173. doi:10.1378/chest.07-2654

4. Nadpara PA, Madhavan SS, Tworek C, Sambamoorthi U, Hendryx M, Almubarak M. Guideline-concordant lung cancer care and associated health outcomes among elderly patients in the United States. *J Geriatr Oncol*. 2015;6(2):101-110. doi:10.1016/j.jgo.2015.01.001

5. Corner J, Hopkinson J, Fitzsimmons D, Barclay S, Muers M. Is late diagnosis of lung cancer inevitable? Interview study of patients' recollections of symptoms before diagnosis. *Thorax*. 2005;60(4):314-319. doi:10.1136/thx.2004.029264

For peer review only

6. Chowienczyk S, Price S, Hamilton W. Changes in the presenting symptoms of lung cancer from 2000–2017: a serial cross-sectional study of observational records in UK primary care. *Br J Gen Pract*. 2020;70(692):e193-e199. doi:10.3399/bjgp20X708137

7. Walter FM, Rubin G, Bankhead C, et al. Symptoms and other factors associated with time to diagnosis and stage of lung cancer: a prospective cohort study. *Br J Cancer*. 2015;112(S1):S6-S13. doi:10.1038/bjc.2015.30

8. Hamilton W. What are the clinical features of lung cancer before the diagnosis is made? A population based case-control study. *Thorax*. 2005;60(12):1059-1065. doi:10.1136/thx.2005.045880

9. Ades AE, Biswas M, Welton NJ, Hamilton W. Symptom lead time distribution in lung cancer: natural history and prospects for early diagnosis. *Int J Epidemiol*. 2014;43(6):1865-1873. doi:10.1093/ije/dyu174

10. Redaniel MT, Martin RM, Ridd MJ, Wade J, Jeffreys M. Diagnostic Intervals and Its Association with Breast, Prostate, Lung and Colorectal Cancer Survival in England: Historical Cohort Study Using the Clinical Practice Research Datalink. Metze K, ed. *PLOS ONE*. 2015;10(5):e0126608. doi:10.1371/journal.pone.0126608

11. Tod AM, Craven J, Allmark P. Diagnostic delay in lung cancer: a qualitative study: Diagnostic delay in lung cancer. *J Adv Nurs*. 2008;61(3):336-343. doi:10.1111/j.1365-2648.2007.04542.x

12. Mitchell ED, Rubin G, Macleod U. Understanding diagnosis of lung cancer in primary care: qualitative synthesis of significant event audit reports. *Br J Gen Pract*. 2013;63(606):e37-e46. doi:10.3399/bjgp13X660760

13. Menon U, Vedsted P, Zalounina Falborg A, et al. Time intervals and routes to diagnosis for lung cancer in 10 jurisdictions: cross-sectional study findings from the International Cancer Benchmarking Partnership (ICBP). *BMJ Open*. 2019;9(11):e025895. doi:10.1136/bmjopen-2018-025895

For peer review only

Appendix 3. Span-based Event Extractor

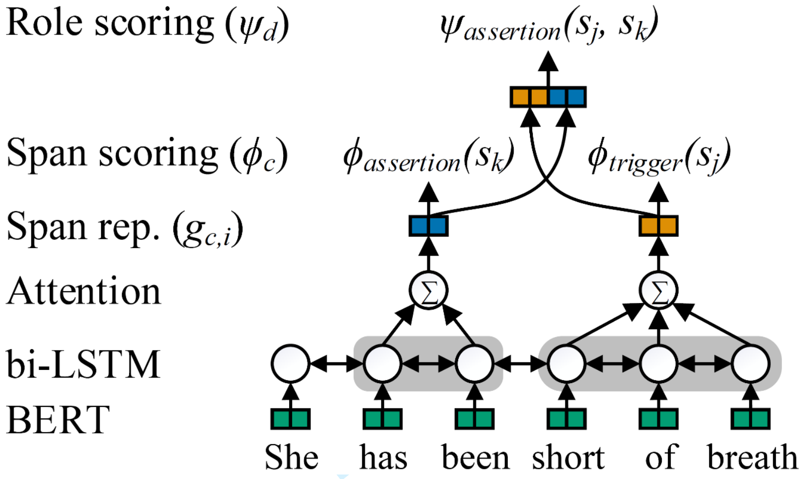

For peer review only

Appendix 4. Comparison of the number of patients with symptoms and signs extracted from the electronic medical record of cases or controls from coded fields versus free-text data using natural language processing (NLP)

Symptom or sign	Identified from NLP (% of patients)	Identified from coded data (% of patients)	Identified from either coded data or NLP (% of patients)	NLP adds (NLP adds n/coded or NLP n)
Cough	1700 (22.6%)	1139 (15.1%)	2227 (29.5%)	1088 (48.9%)
Shortness of breath	1580 (21.0%)	1111 (14.7%)	2128 (28.2%)	1017 (47.8%)
Chest Pain	1241 (16.5%)	981 (13.0%)	1804 (23.9%)	823 (45.6%)
Fatigue	1489 (19.8%)	959 (12.7%)	2063 (27.4%)	1104 (53.5%)
Shoulder pain	513 (6.8%)	594 (7.9%)	893 (11.9%)	299 (33.5%)
Dizziness	1331 (17.7%)	536 (7.1%)	1618 (21.5%)	1082 (66.9%)
Ankle swelling	2081 (27.6%)	509 (6.8%)	2285 (30.3%)	1776 (77.7%)
Headache	1281 (17.0%)	415 (5.5%)	1509 (20.0%)	1094 (72.5%)
Weight loss	646 (8.6%)	328 (4.4%)	830 (11.0%)	502 (60.5%)
Fever	1517 (20.1%)	252 (3.3%)	1656 (22.0%)	1404 (84.8%)
Chest crackles or wheeze	834 (11.1%)	242 (3.2%)	972 (12.9%)	730 (75.1%)
Lympadenopathy	52 (0.7%)	223 (3.0%)	256 (3.4%)	33 (12.9%)
Bone pain	829 (11.0%)	216 (2.9%)	995 (13.2%)	779 (78.3%)
Muscle weakness	1327 (17.6%)	205 (2.7%)	1436 (19.1%)	1231 (85.7%)
Back pain	1220 (16.2%)	154 (2.0%)	1296 (17.2%)	1142 (88.1%)
Changes in sleep	662 (8.8%)	137 (1.8%)	765 (10.2%)	628 (82.1%)
Hoarseness	130 (1.7%)	118 (1.6%)	200 (2.7%)	82 (41.0%)
Hemoptysis	133 (1.8%)	94 (1.3%)	182 (2.4%)	88 (48.4%)
Night sweats	480 (6.4%)	72 (1.0%)	521 (6.9%)	449 (86.2%)
Lack of appetite	626 (8.3%)	59 (0.8%)	653 (8.7%)	594 (91.0%)
Change in bowel habits	1465 (19.4%)	59 (0.8%)	1491 (19.8%)	1432 (96.0%)
Finger clubbing	41 (0.5%)	1 (0.0%)	41 (0.5%)	40 (97.6%)

For peer review only

Appendix 5. Multivariable analysis of symptoms or signs of cases compared to controls at 1, 3, 6 and 12 months prior to diagnosis/index date

Symptom or sign	12 months OR	6 months OR	3 months OR	1 month OR	At diagnosis OR
Finger clubbing	>1,000 (0.0 - >1,000)	>1,000 (0.0 - >1,000)	>1,000 (0.0 - >1,000)	60.7 (10.6 - 348.7)***	50.1 (8.9 - 283.3)***
Lymphadenopathy	0.7 (0.3 - 1.4)	1.3 (0.7 - 2.4)	1.3 (0.8 - 2.3)	1.7 (1.0 - 2.8)*	5.8 (3.8 - 8.8)***
Cough	1.9 (1.5 - 2.4)***	3.1 (2.4 - 4.0)***	4.0 (3.1 - 5.2)***	5.0 (3.8 - 6.5)***	4.7 (3.5 - 6.3)***
Hemoptysis	2.1 (1.0 - 4.4)*	3.2 (1.9 - 5.3)***	3.1 (1.9 - 4.9)***	3.4 (2.2 - 5.4)***	3.5 (2.2 - 5.5)***
Chest crackles or wheeze	2.5 (1.9 - 3.5)***	3.1 (2.3 - 4.1)***	3.0 (2.3 - 4.0)***	3.0 (2.3 - 4.0)***	3.2 (2.4 - 4.3)***
Weight loss	1.2 (0.9 - 1.8)	2.1 (1.5 - 2.8)***	2.6 (1.9 - 3.4)***	2.8 (2.1 - 3.7)***	2.9 (2.2 - 3.9)***
Back pain	2.8 (2.1 - 3.6)***	2.5 (1.9 - 3.2)***	2.5 (1.9 - 3.2)***	2.4 (1.9 - 3.1)***	2.4 (1.8 - 3.1)***
Bone pain	2.8 (2.1 - 3.7)***	2.7 (2.1 - 3.6)***	2.4 (1.8 - 3.2)***	2.3 (1.7 - 3.0)***	2.3 (1.7 - 3.0)***
Shortness of breath	0.7 (0.5 - 1.0)*	1.0 (0.7 - 1.3)	1.3 (1.0 - 1.7)	1.6 (1.2 - 2.1)**	1.9 (1.4 - 2.5)***
Fatigue	1.6 (1.2 - 2.1)***	1.6 (1.3 - 2.1)***	1.9 (1.4 - 2.5)***	1.8 (1.4 - 2.4)***	1.8 (1.3 - 2.3)***
Chest Pain	1.1 (0.8 - 1.4)	1.2 (0.9 - 1.5)	1.2 (1.0 - 1.6)	1.3 (1.0 - 1.6)	1.4 (1.1 - 1.8)*
Shoulder pain	1.3 (0.9 - 1.7)	1.4 (1.0 - 1.8)*	1.3 (1.0 - 1.7)	1.3 (1.0 - 1.7)	1.3 (0.9 - 1.7)
Ankle swelling	1.5 (1.1 - 1.9)**	1.3 (1.0 - 1.7)	1.3 (1.0 - 1.7)	1.3 (1.0 - 1.7)	1.1 (0.9 - 1.5)
Headache	1.0 (0.7 - 1.3)	1.1 (0.8 - 1.4)	1.0 (0.8 - 1.3)	1.0 (0.8 - 1.3)	1.1 (0.8 - 1.4)
Hoarseness	0.9 (0.5 - 1.7)	1.1 (0.7 - 1.8)	1.0 (0.6 - 1.6)	1.1 (0.7 - 1.7)	1.0 (0.7 - 1.7)
Changes in bowel habits	1.2 (0.9 - 1.6)	1.0 (0.8 - 1.4)	1.1 (0.8 - 1.5)	1.0 (0.8 - 1.4)	1.0 (0.8 - 1.4)
Muscle weakness	1.0 (0.7 - 1.3)	0.9 (0.7 - 1.2)	1.0 (0.7 - 1.3)	1.0 (0.8 - 1.3)	1.0 (0.7 - 1.3)
Night sweats	0.9 (0.6 - 1.4)	0.9 (0.7 - 1.4)	0.9 (0.7 - 1.3)	0.9 (0.6 - 1.3)	0.8 (0.6 - 1.2)
Lack of appetite	0.5 (0.3 - 0.7)***	0.6 (0.4 - 0.8)**	0.6 (0.4 - 0.8)**	0.6 (0.4 - 0.9)**	0.7 (0.5 - 0.9)*
Dizziness	0.8 (0.6 - 1.0)	0.7 (0.5 - 0.9)**	0.7 (0.5 - 0.9)**	0.6 (0.5 - 0.8)**	0.6 (0.4 - 0.8)***
Changes in sleep	0.8 (0.5 - 1.1)	0.5 (0.4 - 0.7)***	0.4 (0.3 - 0.6)***	0.4 (0.3 - 0.6)***	0.4 (0.3 - 0.6)***
Fever	0.6 (0.4 - 0.8)***	0.5 (0.4 - 0.7)***	0.5 (0.4 - 0.6)***	0.5 (0.3 - 0.6)***	0.4 (0.3 - 0.6)***

Note: Models adjusted for comorbidities using van Walraven weighted score. Confidence intervals for significant ORs do not incorporate 1.0 due to rounding.

* p<0.05

** p<0.01

*** p<0.001

For peer review only

Appendix 6. Frequency of symptoms and signs in cases and controls with and without chronic respiratory disease

Symptom or sign	Chronic respiratory disease		No chronic respiratory disease	
	Control (n=1252)	Case (n=353)	Control (n=5589)	Case (n=345)
Cough	636 (50.8%)	312 (88.4%)	1018 (18.2%)	261 (75.7%)
Shortness of breath	623 (49.8%)	307 (87.0%)	990 (17.7%)	208 (60.3%)
Fatigue	459 (36.7%)	266 (75.4%)	1128 (20.2%)	210 (60.9%)
Ankle swelling	516 (41.2%)	250 (70.8%)	1322 (23.7%)	197 (57.1%)
Chest Pain	439 (35.1%)	228 (64.6%)	962 (17.2%)	175 (50.7%)
Chest crackles or wheeze	307 (24.5%)	268 (75.9%)	268 (4.8%)	129 (37.4%)
Back pain	278 (22.2%)	191 (54.1%)	668 (12.0%)	159 (46.1%)
Changes in bowel habits	337 (26.9%)	195 (55.2%)	818 (14.6%)	141 (40.9%)
Muscle weakness	327 (26.1%)	177 (50.1%)	775 (13.9%)	157 (45.5%)
Fever	433 (34.6%)	177 (50.1%)	901 (16.1%)	145 (42.0%)
Weight loss	165 (13.2%)	191 (54.1%)	357 (6.4%)	117 (33.9%)
Headache	324 (25.9%)	175 (49.6%)	881 (15.8%)	129 (37.4%)
Dizziness	366 (29.2%)	174 (49.3%)	953 (17.1%)	125 (36.2%)
Bone pain	207 (16.5%)	141 (39.9%)	518 (9.3%)	129 (37.4%)
Lack of appetite	142 (11.3%)	116 (32.9%)	315 (5.6%)	80 (23.2%)
Shoulder pain	200 (16.0%)	92 (26.1%)	513 (9.2%)	88 (25.5%)
Lymphadenopathy	35 (2.8%)	79 (22.4%)	70 (1.3%)	72 (20.9%)
Night sweats	113 (9.0%)	89 (25.2%)	258 (4.6%)	61 (17.7%)
Changes in sleep	178 (14.2%)	90 (25.5%)	453 (8.1%)	44 (12.8%)
Hemoptysis	31 (2.5%)	72 (20.4%)	36 (0.6%)	43 (12.5%)
Hoarseness	55 (4.4%)	45 (12.7%)	78 (1.4%)	22 (6.4%)
Finger clubbing	1 (0.1%)	28 (7.9%)	1 (0.0%)	11 (3.2%)

For peer review only

Appendix 7. Multivariate analysis of symptoms and signs in patients with and without chronic respiratory disease

Symptom or sign	Chronic respiratory disease			No chronic respiratory disease		
	Univariate Odds ratio (95%CI)	Multivariate Odds ratio (95%CI)	Multivariate P value	Univariate Odds ratio (95%CI)	Multivariate Odds ratio (95%CI)	Multivariate P value
Finger clubbing	47.3 (6.1 - 364.5)	17.8 (1.3 - 247.1)	0.0322	>1,000 (0.0 - >1,000)	267.7 (0.1 - >1,000)	0.1783
Chest crackles or wheeze	9.4 (6.3 - 14.2)*	4.9 (2.6 - 9.0)	<0.0001	9.8 (7.0 - 13.9)*	3.2 (2.0 - 5.2)	<0.0001
Hemoptysis	12.5 (6.2 - 25.3)*	4.4 (1.7 - 11.5)	0.0028	20.3 (10.2 - 40.5)*	3.8 (1.5 - 9.8)	0.0049
Weight loss	7.1 (4.7 - 10.5)*	4.0 (2.2 - 7.4)	<0.0001	3.8 (2.8 - 5.3)*	1.6 (1.0 - 2.5)	0.0643
Lympadenopathy	7.1 (3.9 - 13.0)*	3.3 (1.3 - 7.9)	0.0089	12.0 (7.2 - 19.9)*	8.5 (4.3 - 17.0)	<0.0001
Fatigue	5.2 (3.6 - 7.6)*	2.9 (1.6 - 5.5)	0.0008	4.2 (3.2 - 5.6)*	1.7 (1.1 - 2.6)	0.0128
Back pain	4.6 (3.2 - 6.6)*	2.4 (1.4 - 4.1)	0.0014	4.8 (3.6 - 6.4)*	2.1 (1.4 - 3.2)	0.0003
Cough	6.5 (4.2 - 10.2)*	2.2 (1.1 - 4.3)	0.0189	12.2 (9.0 - 16.6)*	6.3 (4.2 - 9.3)	<0.0001
Bone pain	3.8 (2.6 - 5.5)*	2.1 (1.1 - 4.0)	0.0168	5.3 (3.9 - 7.2)*	2.5 (1.6 - 3.9)	0.0001
Shortness of breath	6.5 (4.1 - 10.3)*	1.6 (0.8 - 3.2)	0.1688	5.1 (3.9 - 6.7)*	1.9 (1.3 - 2.9)	0.0024
Changes in bowel habits	2.7 (2.0 - 3.8)*	1.3 (0.7 - 2.3)	0.4474	2.5 (1.9 - 3.4)*	0.9 (0.6 - 1.4)	0.7286
Night sweats	3.1 (2.1 - 4.7)*	1.2 (0.6 - 2.4)	0.5393	3.8 (2.6 - 5.7)*	0.9 (0.5 - 1.7)	0.8542
Ankle swelling	2.8 (2.0 - 3.9)*	1.1 (0.6 - 2.0)	0.6696	3.1 (2.4 - 4.0)*	1.2 (0.8 - 1.8)	0.3121
Shoulder pain	1.6 (1.1 - 2.4)	1.1 (0.6 - 2.0)	0.7589	2.9 (2.1 - 4.0)*	1.6 (1.0 - 2.5)	0.0484
Hoarseness	2.5 (1.4 - 4.4)	1.0 (0.5 - 2.3)	0.9617	4.1 (2.2 - 7.7)*	0.9 (0.4 - 2.2)	0.8729
Headache	2.5 (1.9 - 3.5)*	0.9 (0.5 - 1.7)	0.8551	2.2 (1.7 - 2.9)*	1.0 (0.7 - 1.6)	0.8319
Chest Pain	2.6 (1.9 - 3.6)*	0.9 (0.5 - 1.6)	0.7953	3.7 (2.8 - 4.8)*	1.5 (1.0 - 2.2)	0.0494
Muscle weakness	2.3 (1.7 - 3.2)*	0.9 (0.5 - 1.7)	0.7901	3.1 (2.3 - 4.1)*	1.1 (0.7 - 1.7)	0.6809
Dizziness	2.3 (1.7 - 3.3)*	0.9 (0.5 - 1.6)	0.7450	1.8 (1.3 - 2.4)*	0.5 (0.3 - 0.8)	0.0027
Lack of appetite	2.6 (1.8 - 3.8)*	0.5 (0.3 - 1.0)	0.0667	1.8 (1.3 - 2.6)	0.5 (0.3 - 0.9)	0.0122
Changes in sleep	1.6 (1.1 - 2.3)	0.5 (0.3 - 0.9)	0.0233	1.1 (0.7 - 1.6)	0.3 (0.2 - 0.6)	0.0004
Fever	1.6 (1.2 - 2.2)	0.3 (0.2 - 0.6)	0.0003	2.5 (1.9 - 3.3)*	0.6 (0.4 - 0.9)	0.0229

Note: Models adjusted for comorbidities using van Walraven weighted score

*Significant at p<0.0001

For peer review only

STROBE Statement—Checklist of items that should be included in reports of ***case-control studies***

	Item No	Recommendation	Page No
**Title and abstract**	1	(*a*) Indicate the study's design with a commonly used term in the title or the abstract	1, 3
		(*b*) Provide in the abstract an informative and balanced summary of what was done and what was found	3
**Introduction**			
Background/rationale	2	Explain the scientific background and rationale for the investigation being reported	5
Objectives	3	State specific objectives, including any prespecified hypotheses	5
**Methods**			
Study design	4	Present key elements of study design early in the paper	5,6
Setting	5	Describe the setting, locations, and relevant dates, including periods of recruitment, exposure, follow-up, and data collection	6, 7, 8
Participants	6	(*a*) Give the eligibility criteria, and the sources and methods of case ascertainment and control selection. Give the rationale for the choice of cases and controls	6-8
		(*b*) For matched studies, give matching criteria and the number of controls per case	6-8
Variables	7	Clearly define all outcomes, exposures, predictors, potential confounders, and effect modifiers. Give diagnostic criteria, if applicable	6-8
Data sources/ measurement	8*	For each variable of interest, give sources of data and details of methods of assessment (measurement). Describe comparability of assessment methods if there is more than one group	6-8
Bias	9	Describe any efforts to address potential sources of bias	6-8
Study size	10	Explain how the study size was arrived at	9
Quantitative variables	11	Explain how quantitative variables were handled in the analyses. If applicable, describe which groupings were chosen and why	7-8
Statistical methods	12	(*a*) Describe all statistical methods, including those used to control for confounding	8-9
		(*b*) Describe any methods used to examine subgroups and interactions	8-9
		(*c*) Explain how missing data were addressed	8-9
		(*d*) If applicable, explain how matching of cases and controls was addressed	8-9
		(*e*) Describe any sensitivity analyses	8-9
**Results**			
Participants	13*	(a) Report numbers of individuals at each stage of study—eg numbers potentially eligible, examined for eligibility, confirmed eligible, included in the study, completing follow-up, and analysed	9
		(b) Give reasons for non-participation at each stage	9
		(c) Consider use of a flow diagram	Figure 1
Descriptive data	14*	(a) Give characteristics of study participants (eg demographic, clinical, social) and information on exposures and potential confounders	9-10
		(b) Indicate number of participants with missing data for each variable of interest	10-11

For peer review only - http://bmjopen.bmj.com/site/about/guidelines.xhtml

Outcome data	15*	Report numbers in each exposure category, or summary measures of exposure	9-11

For peer review only

Main results	16	(*a*) Give unadjusted estimates and, if applicable, confounder-adjusted estimates and their precision (eg, 95% confidence interval). Make clear which confounders were adjusted for and why they were included	10-11
		(*b*) Report category boundaries when continuous variables were categorized	n/a
		(*c*) If relevant, consider translating estimates of relative risk into absolute risk for a meaningful time period	n/a
Other analyses	17	Report other analyses done—eg analyses of subgroups and interactions, and sensitivity analyses	11-12
**Discussion**			
Key results	18	Summarise key results with reference to study objectives	12
Limitations	19	Discuss limitations of the study, taking into account sources of potential bias or imprecision. Discuss both direction and magnitude of any potential bias	13-14
Interpretation	20	Give a cautious overall interpretation of results considering objectives, limitations, multiplicity of analyses, results from similar studies, and other relevant evidence	14-15
Generalisability	21	Discuss the generalisability (external validity) of the study results	14-15
**Other information**			
Funding	22	Give the source of funding and the role of the funders for the present study and, if applicable, for the original study on which the present article is based	16

*Give information separately for cases and controls.

Note: An Explanation and Elaboration article discusses each checklist item and gives methodological background and published examples of transparent reporting. The STROBE checklist is best used in conjunction with this article (freely available on the Web sites of PLoS Medicine at http://www.plosmedicine.org/, Annals of Internal Medicine at http://www.annals.org/, and Epidemiology at http://www.epidem.com/). Information on the STROBE Initiative is available at http://www.strobe-statement.org.

For peer review only

BMJ Open

Symptoms and signs of lung cancer prior to diagnosis: Case-control study using electronic health records from ambulatory care within a large US-based tertiary care center

Journal:	*BMJ Open*
Manuscript ID	bmjopen-2022-068832.R2
Article Type:	Original research
Date Submitted by the Author:	21-Mar-2023
Complete List of Authors:	Prado, Maria G.; University of Washington, Department of Family Medicine<br>Kessler, Larry ; University of Washington, Health Services<br>Au, Margaret A; University of Washington, Department of Family Medicine<br>Burkhardt, Hannah; University of Washington, Department of Biomedical Informatics and Medical Education<br>Zigman Suchsland, Monica; University of Washington<br>Kowalski, Lesleigh; University of Washington, Department of Family Medicine<br>Stephens, KA; University of Washington, Department of Family Medicine<br>Yetisgen, Meliha; University of Washington, Department of Biomedical Informatics and Medical Education<br>Walter, Fiona M; Queen Mary University of London, Wolfson Institute of Population Health, Barts and The London School of Medicine and Dentistry; University of Cambridge, The Primary Care Unit Department of Public Health and Primary Care<br>Neal, Richard; University of Exeter<br>Lybarger, Kevin; George Mason University, Department of Information Sciences and Technology<br>Thompson, Caroline; The University of North Carolina at Chapel Hill, Department of Epidemiology; San Diego State University<br>Al Achkar, Morhaf; University of Washington, Department of Family Medicine<br>Sarma, Elizabeth; NIH, National Cancer Institute<br>Turner, Grace; University of Washington, Department of Biomedical Informatics and Medical Education<br>Farjah, Farhood ; University of Washington, Department of Surgery<br>Thompson, Matthew; University of Washington, Department of Family Medicine
**Primary Subject Heading**:	Oncology
Secondary Subject Heading:	Health informatics, Diagnostics, General practice / Family practice
Keywords:	Health informatics < BIOTECHNOLOGY & BIOINFORMATICS, Thoracic medicine < INTERNAL MEDICINE, Respiratory tract tumours <

	ONCOLOGY, PRIMARY CARE

SCHOLARONE™
Manuscripts

I, the Submitting Author has the right to grant and does grant on behalf of all authors of the Work (as defined in the below author licence), an exclusive licence and/or a non-exclusive licence for contributions from authors who are: i) UK Crown employees; ii) where BMJ has agreed a CC-BY licence shall apply, and/or iii) in accordance with the terms applicable for US Federal Government officers or employees acting as part of their official duties; on a worldwide, perpetual, irrevocable, royalty-free basis to BMJ Publishing Group Ltd ("BMJ") its licensees and where the relevant Journal is co-owned by BMJ to the co-owners of the Journal, to publish the Work in this journal and any other BMJ products and to exploit all rights, as set out in our licence.

The Submitting Author accepts and understands that any supply made under these terms is made by BMJ to the Submitting Author unless you are acting as an employee on behalf of your employer or a postgraduate student of an affiliated institution which is paying any applicable article publishing charge ("APC") for Open Access articles. Where the Submitting Author wishes to make the Work available on an Open Access basis (and intends to pay the relevant APC), the terms of reuse of such Open Access shall be governed by a Creative Commons licence – details of these licences and which Creative Commons licence will apply to this Work are set out in our licence referred to above.

Other than as permitted in any relevant BMJ Author's Self Archiving Policies, I confirm this Work has not been accepted for publication elsewhere, is not being considered for publication elsewhere and does not duplicate material already published. I confirm all authors consent to publication of this Work and authorise the granting of this licence.

For peer review only

Symptoms and signs of lung cancer prior to diagnosis: Case-control study using electronic health records from ambulatory care within a large US-based tertiary care center

Maria G. Prado 1

Larry G. Kessler 3

Margaret A Au 1

Hannah Burkhardt 2

Monica Zigman Suchsland 1

Lesleigh Kowalski 1

Kari A. Stephens 1

Meliha Yetisgen 2

Fiona M. Walter 5,6

Richard D Neal 7

Kevin Lybarger 11

Caroline Thompson 8, 9

Morhaf Al Achkar 1

Elizabeth A. Sarma 10

Grace Turner 2

Farhood Farjah 4

Matthew Thompson 1

Affiliations

1 Department of Family Medicine, University of Washington, Seattle, WA, USA

2 Department of Biomedical Informatics and Medical Education, University of Washington, Seattle, WA, USA

For peer review only

3 Department of Health Systems and Population Health, School of Public Health, University of Washington, Seattle, WA, USA

4 Department of Surgery, University of Washington, Seattle, WA, USA

5 Wolfson Institute of Population Health, Barts and The London School of Medicine and Dentistry, Queen Mary University of London, UK

6 The Primary Care Unit, Department of Public Health and Primary Care, University of Cambridge, UK

7 University of Exeter Medical School, University of Exeter, Exeter, UK

8 Department of Epidemiology, Gillings School of Global Public Health, The University of North Carolina at Chapel Hill, Chapel Hill, NC, USA

9 Division of Epidemiology and Biostatistics, School of Public Health, San Diego State University, San Diego, CA, USA

10 Healthcare Delivery Research Program, Division of Cancer Control and Population Sciences, National Cancer Institute, Bethesda, MD, USA

11 Department of Information Sciences and Technology, George Mason University, Fairfax, VA, USA

Corresponding author: Matthew Thompson.

mjt@uw.edu

University of Washington, Box 354696

4225 Roosevelt NE, Suite 308, Seattle, WA 98105

Tel #: (206) 616-8149

For peer review only

Abstract

Objective: Lung cancer is the most common cause of cancer-related death in the United States (US). While most patients are diagnosed following symptomatic presentation, no studies have compared symptoms and physical examination signs at or prior to diagnosis from electronic health records (EHR) in the US. We aimed to identify symptoms and signs in patients prior to diagnosis in EHR data.

Design: Case-control study

Setting: Ambulatory care clinics at a large tertiary care academic health center in the US

Participants, Outcomes: We studied 698 primary lung cancer cases in adults diagnosed between January 1, 2012 and December 31, 2019, and 6,841 controls matched by age, sex, smoking status, and type of clinic. Coded and free-text data from the EHR were extracted from 2 years prior to diagnosis date for cases and index date for controls. Univariate and multivariable conditional logistic regression were used to identify symptoms and signs associated with lung cancer at time of diagnosis, and 1, 3, 6, and 12 months before the diagnosis/index dates.

Results: Eleven symptoms and signs recorded during the study period were associated with a significantly higher chance of being a lung cancer case in multivariable analyses. Of these, seven were significantly associated with lung cancer six months prior to diagnosis: hemoptysis (OR 3.2, 95%CI 1.9-5.3), cough (OR 3.1, 95%CI 2.4-4.0), chest crackles or wheeze (OR 3.1, 95%CI 2.3-4.1), bone pain (OR 2.7, 95%CI 2.1-3.6), back pain (OR 2.5, 95%CI 1.9-3.2), weight loss (OR 2.1, 95%CI 1.5-2.8) and fatigue (OR 1.6, 95%CI 1.3-2.1).

Conclusions: Patients diagnosed with lung cancer appear to have symptoms and signs recorded in the EHR that distinguish them from similar matched patients in ambulatory care, often six months or more before diagnosis. These findings suggest opportunities to improve the diagnostic process for lung cancer.

For peer review only

Strengths and limitations of this study

Strengths

- Using Natural Language Processing (NLP) techniques to extract symptoms and signs from unstructured data provides a more complete dataset of clinical features presence compared to using coded data alone.
- Case control design recruited cases from ambulatory care population, and controls were randomly selected in a 10:1 ratio based on case clinic type, to reduce the possibility of bias.

Limitations

- Criteria for selection of cases and controls differed slightly; Cases were selected based on a date of the first lung cancer diagnostic code in the EHR, whereas controls were selected based on having a visit to the matched type of clinic type within 3 months of the case diagnosis date.
- Controls were not linked to cancer registry, so it is possible, though we believe highly unlikely, that there were a few cases among our controls who had a diagnosis of lung cancer in the cancer registry but no such diagnosis recorded in the EHR at any time (in our time window).
- Availability and timing of symptom data for cases and controls is based on number and frequency of patient interactions with the healthcare system which could be due to a range of factors.

For peer review only

Introduction

Lung cancer is the third most common cancer and the leading cause of cancer death in the United States (US).[1] Most patients with lung cancer are diagnosed following presentation to healthcare settings with symptoms or diagnosed incidentally, and many patients (47%) present with late-stage disease (stages 3 or 4).[2] Screening for lung cancer remains low in the US, with an estimated 6.6% of adults receiving screening in 2019.[3,4] In addition to optimizing screening, early detection efforts have focused on recognition of lung cancer symptoms with an overall goal of identifying patients at earlier, more treatable stages of the disease.[5–7] These symptoms range from 'alarm' symptoms, such as hemoptysis (a rare symptom), to relatively non-specific symptoms, such as persistent cough or unexpected weight loss.[6]

Diagnosing lung cancer based on non-specific symptom presentation is challenging, as these symptoms are more commonly associated with benign conditions or may be overlooked for long periods of time. A study of over 43 million patients using Medicare claims data identified a median time from symptom onset to diagnosis of approximately six months.[8] However, claims data lack the granularity needed to identify which clinical features patients present and how these might be used to differentiate patients with lung cancer from the vast majority of patients with benign conditions. To fill this gap, we examined the frequency and association of symptoms and physical examination signs in patients in ambulatory care prior to lung cancer diagnosis and matched controls.

Methods

Study design

We performed a case-control study using data from the University of Washington Medicine (UWM) electronic health records (EHR) and the Seattle/Puget Sound Surveillance, Epidemiology, and End Results (SEER) Program, a National Cancer Institute-supported national cancer registry.[9] This study was approved by the University of Washington Human Subjects Division (STUDY 000013191). A patient and caregiver stakeholder group was involved over a period of 2 years involving regular meetings in the design of this study and in the interpretation of the findings.

For peer review only - http://bmjopen.bmj.com/site/about/guidelines.xhtml

Setting

Cases and controls were identified from patients who received ambulatory care at UWM, a large tertiary care academic health center.

Participants

Cases were identified from UWM patients aged 18 years or older, with a first primary lung cancer diagnosis (see International Classification of Diseases (ICD) 9 and 10 codes in Appendix 1) between January 1, 2012 and December 31, 2019, who had an established relationship with a UWM ambulatory care setting in the 2 years before the date of their first recorded lung cancer ICD code in the EHR (EHR diagnosis date). We chose the above study period because of the limited quality of the UWM EHR data prior to 2012. We defined ambulatory care as at least one encounter in family medicine, internal medicine, women's health, obstetrics and gynecology, urgent care, and/or emergency medicine. We used linkage to the regional SEER registry to verify cancer incident cases. Cases were excluded if they did not match with the SEER registry, or if they had a first primary tumor located in anatomy other than the lung, or had evidence of a history of any of the following cancers identified using histology codes in SEER: tracheal cancer, mesothelioma, Kaposi sarcoma, lymphoma, or leukemia. Controls were identified from UWM patients with at least one encounter with the same type of ambulatory clinic within 3 months of the EHR diagnosis date of the index case (matching date). This 3-month window was chosen to avoid potential seasonal differences in respiratory symptoms. For each case, 10 controls were individually matched to the index case by age, sex (male, female), smoking status (ever vs. never), and type of ambulatory care clinic where lung cancer case presented (emergency medicine vs other clinics listed above). We chose a 10:1 control: case match because we recognize the wide variety of patients presenting to ambulatory care settings. Controls were excluded if they had any lung cancer ICD codes in their EHR prior to their matched case diagnosis (index) date. Excluded cancers in cases (based on histology codes from the SEER registry) were not identified in controls as registry data was not available for controls. We also excluded any cases and controls who did not have any ICD codes in any

For peer review only - http://bmjopen.bmj.com/site/about/guidelines.xhtml

encounter in the 2 years prior to diagnosis date (cases) or index date (controls) to ensure availability of data on pre-diagnosis symptoms and signs.

Data Collection

The UWM enterprise-wide data warehouse (EDW) was used to obtain data; this provides a central repository that integrates EHR across the UWM health care system including ambulatory care, specialty care and hospital services. Cases were identified during the study period using ICD codes (Appendix 1) and were linked to SEER to ensure accuracy of case identification and obtain history of previous cancers, histology (for exclusions and lung cancer type), and stage at diagnosis. The date of diagnosis was determined by date of pathology report at UWM. For cases that did not have a diagnosis through pathology or had a discrepancy greater than 30 days between date of pathology and first recorded lung cancer ICD code, two of three clinicians (MT, LKF, MAlA) reviewed the EHR of these cases to adjudicate dates. Controls were randomly sampled from within the matching strata, based on this adjudicated date of diagnosis.

Cases who had undergone lung cancer screening using low-dose computed tomography (LDCT) within the 12 months prior to diagnosis date were identified from billing code (Current Procedural Terminology or CPT 71271) and/or ICD codes (V76.0 [ICD-9] or Z12.2 [ICD-10].

An EHR data extraction protocol was applied to all encounters in the 2-year period prior and up to six months following the diagnosis date (cases) and index date (controls). These data comprised of demographics (e.g., age, sex, race, ethnicity), all ICD codes and CPT procedure codes linked to encounters such as laboratory tests, imaging procedures, and pathology data. We also extracted corresponding unstructured clinical notes for any of the above encounters from inpatient and outpatient settings. Clinical note types included progress notes, telephone encounters, hospital admission and discharge notes, notes of consultations with generalist and specialist clinicians, and nursing record notes. ICD codes recorded during the 2-year period prior to diagnosis for cases or prior to index date for controls were searched for the presence of 31 potential comorbidities to calculate the Elixhauser comorbidity index.[10] We excluded lung

For peer review only - http://bmjopen.bmj.com/site/about/guidelines.xhtml

cancer ICD code information from this calculation. These index scores were then used to calculate van Walraven weighted scores for each patient, a range of -19 to 89.[11,12]

Symptoms and signs

We identified symptoms and signs using coded data and unstructured data. A list of symptoms and signs which have previously been reported in cohort or case-control studies of individuals with lung cancer were identified from systematic reviews, hand review of individual studies, and from contact with experts in oncology, cardiothoracic surgery, and primary care (FW, RN, FF, MT, see Appendix 2).[5,6,13–18] These were mapped to ICD codes, and used to search the extracted EHR coded data for any encounters that included any of these ICD codes in the 2-year observation period.

Symptoms and signs were automatically extracted from free-text clinical notes using natural language processing (NLP), including notes for all visit types in the 2-year period. In previous work, we developed a deep learning symptom extraction model that generates structured semantic representations of symptoms.[19] The annotation scheme and extraction architecture from this prior work represents symptoms using event-based approach. Each symptom event includes a trigger span that identifies the specific symptom (e.g. "cough" or "shortness of breath") and multiple attributes that characterize the symptom. The attributes most relevant to this work are the *Assertion* value, which indicates whether the symptom is *present*, *absent*, *possible*, etc., and the *Anatomy*, which indicates the anatomical location of the symptom (e.g. "chest wall" or "lower back").

Structured symptom predictions were generated using the Span-based Event Extractor architecture in Appendix 3. Each clinical note is split into sentences, which feed into the extractor. The words (tokens) of each sentence are mapped to a vector space using a clinical version of the Bidirectional Encoder Representations from Transformers (BERT) model (no model fine-tuning). The BERT mapping of each sentence then feeds into a bidirectional Long Short-Term Memory (LSTM) network, which adapts the BERT encoding to the target extraction task. All possible token spans for the sentence are enumerated, and self-attention is used to

For peer review only - http://bmjopen.bmj.com/site/about/guidelines.xhtml

create a representation for each span, $g_{c,i}$. Each of the enumerated spans is then classified using feedforward neural networks, ϕ_c, that operate on the span representation, $g_{c,i}$. The span scoring layer, ϕ_c, identifies the symptom triggers and attributes. Clinical notes frequently describe multiple symptoms within a sentence, and the relationships between the identified symptoms and attributes must be resolved. The identified symptom triggers are paired with the associated symptom attributes through the role scoring layer, ψ_d, which consists of a feedforward neural network that operates on span representation pairs. The output of the Span-based Event Extractor is a structured symptom representation, where identified symptoms are assigned multiple attributes.

In our original symptom work, we trained the Span-based Event Extractor on the COVID-19 Annotated Clinical Text Corpus (CACT).[19] To support the current research, we adapted the symptom extractor to the lung cancer domain. The domain adaptation involved creating the Lung Cancer Annotated Clinical Text (LACT) Corpus, composed of 270 notes from lung cancer patients (170 training and 100 test notes).[20] We trained the lung cancer symptom extractor by combining the CACT and LACT training sets. On the LACT test set, the lung cancer symptom extractor achieved 0.72 F1 for symptom identification and 0.65 F1 for assertion prediction. This extraction performance is comparable to the LACT inter-rater agreement of 0.82 F1 for symptom identification and 0.79 F1 for assertion prediction, indicating the model is achieving approximately human-level performance. We included the extracted symptoms and signs with assertion value present. All models were developed using the Python deep learning packages by PyTorch and Transformers.[21,22] The Span-based Event Extractor will be released through UW-BIoNLP github (https://github.com/uw-bionlp). The clinical notes will not be released for confidentiality purposes.

Data analysis

Frequencies and counts were calculated for characteristics of cases and controls. The number of symptoms and signs obtained from coded data was compared to that obtained from free-text data using descriptive statistics. The proportion of patients with evidence of each

For peer review only - http://bmjopen.bmj.com/site/about/guidelines.xhtml

symptom/sign occurring in the 2-year period prior to the diagnosis or index date was described for cases and controls. Odds of patients' case status, based on symptoms and signs identified from a combined dataset of coded and free-text data, were estimated using unadjusted conditional logistic regression. Symptoms and signs associated with lung cancer in unadjusted regressions ($p < 0.1$) were included into multivariable conditional logistic regression analyses. We used the van Walraven comorbidity score to adjust for population differences in comorbidity burden. Analyses were repeated excluding symptom and sign data from 1, 3, 6, and 12 months before the diagnosis (or index) date. Lag times were chosen to provide information on the pattern of symptom-related visits over time and identify the symptoms and signs presenting furthest from diagnosis. We conducted secondary analyses investigating the potential effect of chronic respiratory disease (CRD) status, as defined by the presence of ICD codes within the Elixhauser chronic respiratory disease subgroup, on presence of symptoms and signs in the pre-diagnostic interval. We expected patients with CRD to present with symptoms and signs similar to those that present in early lung cancer. We assessed the effect of CRD by repeating the conditional logistic regression model including CRD as a covariate.

Statistical analyses were conducted using Python 3.7 with the packages SciPy (version 1.4.1) and Statsmodels (version 0.11.1). The study was reported in line with the STROBE guidelines.[23]

Patient and public involvement

We established a technical expert panel (TEP) that included patients with lung cancer and caregivers of patients with lung cancer. The TEP reflected on their personal experience with lung cancer symptoms as well as the lung cancer symptoms we identified in the EHR. They discussed and advised on study methods, data analysis, and communication and visualization of results.

Results

Participants

For peer review only - http://bmjopen.bmj.com/site/about/guidelines.xhtml

Selection of cases & controls

A total of 7,883 patients with lung cancer ICD codes were identified in the UWM EDW over the study period. Following linkage of these patients and those identified as having a primary lung tumor from SEER, 4,115 patients were identified common to both, including 741 cases. After matching 7,410 controls, a chart review resulted in exclusion of 43 additional cases. Controls that were matched to these 43 cases were excluded (n = 422), resulting in 698 cases matched to 6,841 controls (Figure 1).

Description of cases and controls

Cases and controls were similar in terms of sex and race (cases 50.6% male, 75.5% White; controls 50.5% male, 75.7% White, see Table 1), as well as ethnicity (cases 3.3% Hispanic, controls 3.6%). Cases had higher comorbidity scores (*M* = 14.9, *SD* = 11.6) than controls (*M* = 4.4, *SD* = 8.6). Cases also had a greater median number of health care visits over the 2-year period prior to diagnosis (51.0, 95%CI: 28.0-97.8) than controls (23.0, 95%CI: 9.0-53.0). The difference in median number of health care visits was greater in the last 3-month period prior to the diagnosis/index date (cases 21.0, 95%CI: 12.0-35.0 vs. controls 5.0, 95%CI: 2.0-11.0) than in the 2nd, 3rd, or 4th quarters prior to diagnosis. The stage distribution of cases was as follows: Stage 1- 29%, Stage 2- 7%, Stage 3- 17%, and Stage 4 -42% (5% were Stage 0 or Unknown Stage).

Table 1. Characteristics of patients with lung cancer (cases) and matched controls in ambulatory care

**Characteristic**	**Cases (n=698)**	**Controls (n=6841)**
**Age, years**		
<60	161 (23.1%)	1479 (21.6%)
60-69	257 (36.8%)	2514 (36.7%)
70-79	183 (26.2%)	1865 (27.3%)
80+	97 (13.9%)	983 (14.4%)

For peer review only - http://bmjopen.bmj.com/site/about/guidelines.xhtml

**Race**		
American Indian or Alaska Native	6 (0.9%)	78 (1.1%)
Asian	76 (10.9%)	535 (7.8%)
Black or African American	69 (9.9%)	525 (7.7%)
Multiple races	5 (0.7%)	44 (0.6%)
Native Hawaiian or Other Pacific Islander	4 (0.6%)	40 (0.6%)
Unknown	11 (1.6%)	442 (6.5%)
White	527 (75.5%)	5177 (75.7%)
**Ethnicity**		
Hispanic or Latino	23 (3.3%)	244 (3.6%)
Not Hispanic or Latino	630 (90.3%)	5782 (84.5%)
Unknown	45 (6.4%)	815 (11.9%)
**Sex**		
Male	353 (50.6%)	3452 (50.5%)
**Comorbidity - Elixhauser van Walraven weighted Score, mean (SD)**	14.9 (11.6)	4.4 (8.6)
**Number of clinic visits per patient, median (IQR)**		
In entire data window prior to diagnosis/index	51.0 (28.0 - 97.8)	23.0 (9.0 - 53.0)
In 1st quarter prior to diagnosis/index	21.0 (12.0 - 35.0)	5.0 (2.0 - 11.0)
In 2nd quarter prior to diagnosis/index	7.0 (3.0 - 14.0)	5.0 (2.0 - 11.0)
In 3rd quarter prior to diagnosis/index	7.0 (3.0 - 12.0)	5.0 (2.0 - 11.0)
In 4th quarter prior to diagnosis/index	6.0 (3.0 - 13.0)	5.0 (2.0 - 11.0)

Frequency of symptoms and signs extracted from coded and free-text data

Of the 22 symptoms and signs that we systematically examined, NLP identified 20 of the 22 symptoms and signs in greater proportions of patients affected than from the coded data alone (see Appendix 4). In comparison to coded data, we saw a range of 12.9% to 97.6% greater symptom and signs reports with NLP of textual clinical notes. In contrast, a greater proportion of patients had two symptoms and signs (shoulder pain, lymphadenopathy) identified from coded rather than free-text data.

For peer review only

For peer review only - http://bmjopen.bmj.com/site/about/guidelines.xhtml

Comparison of frequency of symptoms and signs between cases and controls

The frequency of all 22 symptoms and signs examined was higher in cases than controls (see Table 2). Moreover, the ranking of symptoms and signs differed slightly between cases and controls, with cases reporting cough (82.1%), shortness of breath (73.8%), fatigue (68.2%), ankle swelling (64.0%), and chest pain (57.7%), whereas controls reported ankle swelling (26.9%), cough (24.2%), shortness of breath (23.6%), fatigue (23.2%) and chest pain (20.5%) most frequently. Hemoptysis occurred relatively infrequently among cases (16.5%) and rarely among controls (1.0%).

Table 2. Comparison of frequency of symptoms and signs identified in coded or free-text data in cases compared to controls

Symptom or sign	Cases (n=698)	Controls (n=6841)
Cough	573 (82.1%)	1654 (24.2%)
Shortness of breath	515 (73.8%)	1613 (23.6%)
Fatigue	476 (68.2%)	1587 (23.2%)
Ankle swelling	447 (64.0%)	1838 (26.9%)
Chest Pain	403 (57.7%)	1401 (20.5%)
Chest crackles or wheeze	397 (56.9%)	575 (8.4%)
Back pain	350 (50.1%)	946 (13.8%)
Change in bowel habits	336 (48.1%)	1155 (16.9%)
Muscle weakness	334 (47.9%)	1102 (16.1%)
Fever	322 (46.1%)	1334 (19.5%)
Weight loss	308 (44.1%)	522 (7.6%)
Headache	304 (43.6%)	1205 (17.6%)
Dizziness	299 (42.8%)	1319 (19.3%)
Bone pain	270 (38.7%)	725 (10.6%)
Lack of appetite	196 (28.1%)	457 (6.7%)
Shoulder pain	180 (25.8%)	713 (10.4%)
Lymphadenopathy	151 (21.6%)	105 (1.5%)
Night sweats	150 (21.5%)	371 (5.4%)
Changes in sleep	134 (19.2%)	631 (9.2%)
Hemoptysis	115 (16.5%)	67 (1.0%)
Hoarseness	67 (9.6%)	133 (1.9%)
Finger clubbing	39 (5.6%)	2 (0.0%)

Univariate associations of symptoms and signs between cases and controls

For peer review only - http://bmjopen.bmj.com/site/about/guidelines.xhtml

In models adjusted for comorbidity score, when considered independently, all 22 symptoms and signs had odds ratios that were significantly different between cases and controls (all $p <$ 0.0001, see Table 3). The symptoms and signs with the largest odds ratios (OR) significantly associated with a higher chance of being a case were finger clubbing (OR 175.7, 95%CI: 40.1-770.0), hemoptysis (OR 14.5, 95%CI: 10.2-20.8), cough (OR 11.1, 95%CI: 8.8-13.9), chest crackles or wheeze (OR 9.9, 95%CI: 8.1-12.2), and lymphadenopathy (OR 9.4, 95%CI: 6.9-12.8).

Multivariable associations of symptoms and signs between cases and controls

We included all 22 symptoms and signs from the univariate analysis and comorbidity score in a multivariable analysis. After mutual adjustment, 15 had significant ORs (all $p < 0.05$, see Table 3). The presence of 11 symptoms and signs were associated with a significantly higher odds of being a case, with ORs ranging from 1.4 (chest pain) to 50.1 (finger clubbing). The largest ORs were noted for finger clubbing (OR 50.1, 95%CI: 8.9-283.3), lymphadenopathy (OR 5.8, 95%CI: 3.8-8.8), cough (OR 4.7, 95%CI: 3.5-6.3), hemoptysis (OR 3.5, 95%CI: 2.2-5.5) and chest crackles or wheeze (OR 3.2, 95%CI: 2.4-4.3). In contrast, the presence of four symptoms was associated with a significantly higher odds of being a control: fever (OR 0.4, 95%CI: 0.3-0.6), changes in sleep (OR 0.5, 95%CI: 0.3-0.6), dizziness (OR 0.6, 95%CI: 0.4-0.8), and lack of appetite (OR 0.7, 95%CI: 0.5-0.9).

Table 3. Univariate and multivariate analyses of symptoms and signs identified in coded or free-text data of cases compared to controls, adjusted for comorbidity (descending order by multivariate odds ratios)

Symptom or sign	Univariate Odds ratio (95%CI)	Multivariate Odds ratio (95%CI)	Multivariate P value
Finger clubbing	175.7 (40.1 - 770.0)*	50.1 (8.9 - 283.3)	<0.0001
Lymphadenopathy	9.4 (6.9 - 12.8)*	5.8 (3.8 - 8.8)	<0.0001
Cough	11.1 (8.8 - 13.9)*	4.7 (3.5 - 6.3)	<0.0001
Hemoptysis	14.5 (10.2 - 20.8)*	3.5 (2.2 - 5.5)	<0.0001
Chest crackles or wheeze	9.9 (8.1 - 12.2)*	3.2 (2.4 - 4.3)	<0.0001
Weight loss	5.9 (4.8 - 7.2)*	2.9 (2.2 - 3.9)	<0.0001
Back pain	4.7 (3.9 - 5.7)*	2.4 (1.8 - 3.1)	<0.0001
Bone pain	4.6 (3.8 - 5.7)*	2.3 (1.7 - 3.1)	<0.0001
Shortness of breath	6.0 (4.9 - 7.3)*	1.9 (1.4 - 2.5)	<0.0001
Fatigue	4.8 (4.0 - 5.8)*	1.8 (1.4 - 2.4)	<0.0001
Chest Pain	3.6 (3.0 - 4.3)*	1.4 (1.1 - 1.8)	0.0118
Shoulder pain	2.3 (1.8 - 2.8)*	1.3 (1.0 - 1.7)	0.1111

For peer review only - http://bmjopen.bmj.com/site/about/guidelines.xhtml

Ankle swelling	3.3 (2.7 - 4.0)*	1.1 (0.9 - 1.5)	0.3643
Headache	2.5 (2.1 - 3.0)*	1.1 (0.8 - 1.4)	0.5619
Hoarseness	3.5 (2.5 - 5.0)*	1.1 (0.7 - 1.7)	0.8447
Change in bowel habits	3.0 (2.5 - 3.6)*	1.0 (0.8 - 1.4)	0.8880
Muscle weakness	2.9 (2.4 - 3.5)*	1.0 (0.7 - 1.3)	0.9581
Night sweats	3.3 (2.6 - 4.2)*	0.8 (0.6 - 1.2)	0.2998
Lack of appetite	2.6 (2.1 - 3.3)*	0.7 (0.5 - 0.9)	0.0193
Dizziness	2.0 (1.7 - 2.4)*	0.6 (0.4 - 0.8)	0.0004
Changes in sleep	1.3 (1.1 - 1.7)*	0.5 (0.3 - 0.6)	<0.0001
Fever	2.1 (1.7 - 2.5)*	0.4 (0.3 - 0.6)	<0.0001

Note: Conditional logistic regression models adjusted for comorbidities using van Walraven weighted score with each symptom or sign modeled individually (univariate) and mutually adjusted (multivariate)
*Significant at p<0.0001 for univariate analysis

For peer review only

We repeated the multivariable analysis, excluding symptoms and signs recorded in periods of 1, 3, 6 and 12 months prior to diagnosis (see Figure 2). Some symptoms and signs remained significantly associated with cases up to 6 months prior to diagnosis (cough, hemoptysis, chest crackles and wheeze, weight loss, back pain, bone pain, fatigue). Of these, all except weight loss were also significantly associated with cases 12 months prior to diagnosis. Other symptoms and signs became significantly associated with being a case closer to the date of diagnosis: shortness of breath and chest pain (3 months prior to diagnosis), lymphadenopathy and finger clubbing (1 month prior) (see Appendix 5).

Secondary analyses

To determine whether the associations were robust to the presence of CRD, we performed a secondary conditional logistic regression that was adjusted for CRD, along with all our matching variables and comorbidity score. The presence of CRD appeared to have no statistically significant effect when directly added as a covariate (OR: 1.05, 95%CI: (0.81, 1.36, p = 0.7229, see Appendices 6 & 7).

Discussion

Main findings

This is the first case-control study in the US to use routine, prospectively collected EHR data to describe the frequency of symptoms and signs of lung cancer and estimate associations with incident lung cancer cases compared to non-lung cancer patients receiving routine ambulatory

For peer review only - http://bmjopen.bmj.com/site/about/guidelines.xhtml

care in the same time period. Our findings provide unique information on symptoms and signs associated with a higher chance of a patient in ambulatory care being diagnosed with lung cancer, and the duration of these associations prior to their cancer diagnosis. In contrast to prior work on national databases, extracting clinicians' documentation of clinical features from their free text clinical notes using NLP provided more complete symptom identification data, rather than relying on data available only in coded, structured data collected in routine care. Our findings provide evidence-based, quantitative support for the development of decision rules around the diagnostic workup of symptomatic patients, which could lead to the improvement of earlier diagnosis of lung cancer. Of the 22 symptoms and signs studied, 11 were found in adjusted models to be associated with a higher chance of being a lung cancer case, and most of these 11 were present and still significantly associated up to 12 months prior to diagnosis; this suggests opportunities for improved screening practices that may lead to earlier diagnosis and possibly improved outcomes.

Our findings also suggest that the clinical presentation of lung cancer appears to be similar, regardless of the presence of other comorbidities, CRD, or smoking. For patients and clinicians this is important as several of the symptoms or signs we identified may currently be dismissed as being attributable to underlying smoking or comorbid conditions.

Comparison with existing literature

Several of the symptoms and signs we found as having statistically significant odds ratios have been identified in studies using data from ambulatory care in other healthcare systems, especially hemoptysis and cough. However, among the symptoms and signs Hamilton and colleagues (2005) found to be associated with being a lung cancer case in the United Kingdom (UK), loss of appetite had the highest OR (86.0), whereas we failed to identify an association with lung cancer.[5] This may be due to a difference in study populations or our use of NLP in EHR data.

Our findings also provide evidence of the temporality of a 'clinical signal' for lung cancer based on symptoms and signs documented in the EHR, at least six and up to 12 months prior to

For peer review only - http://bmjopen.bmj.com/site/about/guidelines.xhtml

diagnosis, consistent with a Medicare claims study. Data from our study and Nadpara and colleagues' (2015) study, which used claims data, provide evidence for time intervals from first presentation with symptoms to diagnosis that are on the upper range (six months) of those reported using analysis of coded symptoms in primary care databases in several UK and European studies.[8] These describe the overall time interval from first symptom recording in medical records to diagnosis ranging from 3- to 6-months.[6,24,25] While not directly comparable, qualitative research from patients with lung cancer and caregivers describe changes noticeable to the individual more than 12 months before attending a health care visit.[17,26,27]

Strengths and limitations

Using NLP to extract symptoms and signs from unstructured data allowed us to capture a more complete dataset of symptom presence compared to using coded data alone. We selected cases from an empaneled ambulatory care population, where we expected EHR data would be available for the period of interest in this study and attempted to exclude patients who were attending only for secondary or tertiary care provided at UWM. Controls were randomly selected based on case clinic type, to reduce the possibility of bias, and duration of follow-up time and availability of data for cases and controls were similar, particularly in visit frequency. We used a robust design where we matched 10 controls to 1 case, providing greater power and precision, and matched on smoking so that our analyses could not be confounded based on ever vs. never exposure to smoking.

Limitations included criteria for selection of cases and controls differed slightly. As is customary in incident case-control studies, cases were selected based on a diagnosis date defined as the date of the first lung cancer ICD code in the EHR. In this way, we captured the diagnostic path from symptom presentation to diagnosis for all cases. Controls were selected based on having a visit to the matched case clinic type (to account for difference in emergency vs other forms of ambulatory care) within 3 months of the case diagnosis date, however the timing of control selection does not necessarily reflect a "pathway to diagnosis" for some other condition, just recent routine care. Additionally, because we did not link to SEER for the control population, we

For peer review only - http://bmjopen.bmj.com/site/about/guidelines.xhtml

were unable to apply two of the case exclusion criteria to our control sample: 1) no current or prior history of lung cancer in SEER, although we did check the UW EHR for concurrent lung-cancer related ICD codes and medical history so this should be rare, and 2) no prior history of tracheal cancer, mesothelioma, Kaposi sarcoma, lymphoma, or leukemia in SEER. Additionally, EHR data can sometimes be subject to misclassification. For example, detailed EHR smoking history may be unreliable and the EHR does not reliably capture health literacy or socioeconomic status; however, we used a very broad definition of smoking (ever vs. never) and used a comorbidity score to control for health status. Finally, availability and timing of symptom data for cases and controls is based on patient interactions with the healthcare system, not a pre-specified protocol of data collection. Patients who have more contact with their providers (which could be due to a range of factors) may have had more data captured.

Implications for clinicians, researchers, policy makers

Differentiating patients who may have symptoms or signs of lung cancer from those attending ambulatory care is a critical and challenging step in the earlier detection of this cancer. Our findings not only identify the 'red flag' (highly specific, but infrequent) symptoms and signs that primary care providers should be aware of (e.g., hemoptysis), but also highlight which of a larger range of 'non-specific' symptoms and signs should equally raise suspicion such as bone pain and weight loss. Furthermore, our findings support the importance of clinical documentation, and continuity of care to identify and act on sustained changes in patients' clinical presentations.

Confirmation of our findings using datasets from other healthcare systems in the U.S. are needed and could be enhanced by more advanced machine learning modelling to incorporate additional clinical variable including quantitative data such as changes in body weight or results of routinely collected laboratory tests, given emerging evidence for associations between weight loss and minor deviations of hemoglobin or platelet count with incident cancer.[28] Given the low uptake of low dose CT screening for lung cancer in the U.S., our findings provide support for revising current priorities to improve early diagnosis of lung cancer.[29]

For peer review only - http://bmjopen.bmj.com/site/about/guidelines.xhtml

Conclusions

Patients in ambulatory care settings who are subsequently diagnosed with lung cancer appear to have symptoms and signs that distinguish them from other patients, often months before lung cancer diagnosis. To improve earlier detection of lung cancer, interventions are urgently needed that promote earlier screening based on symptomatic presentations in ambulatory care that may lead to an earlier detection and treatment of lung cancer.

For peer review only

Author Contributions: MGP extracted data from UW Medicine and linked to SEER Cancer Registry, supported study management and execution, wrote the manuscript, provided critical comments, edited the manuscript, and approved its final version. LGK assisted with design of the study and supported its execution, provided advice and expertise for study design, analyses and interpretation of data, wrote the manuscript, provided critical comments, edited the manuscript, and approved its final version. MAA performed the analyses, provided advice and expertise for study design, conducted analyses and interpretation of data, provided critical comments, edited the manuscript, and approved its final version. HB supported data extraction and data linkage, assisted with analyses, created figures and tables, assisted with interpretation of data, provided critical comments, edited the manuscript, and approved its final version. MZS assisted with design of the study and supported its execution, extracted data from UW Medicine and linked to SEER Cancer Registry, provided further advice and expertise for study design, and interpretation of data, provided critical comments, edited the manuscript, and approved its final version. LK assisted with design of the study and supported its execution, provided advice and expertise for study design, clinical interpretation of data, provided critical comments, edited the manuscript, and approved its final version. KAS assisted with design of the study, extracted data from UW Medicine and linked to SEER Cancer Registry, provided advice and expertise for study design, interpretation of data, provided critical comments, edited the manuscript, and approved its final version. MY created the natural language annotation tool and extracted free text data, assisted with interpretation of data, provided

critical comments, edited the manuscript, and approved its final version. FMW provided advice and expertise for study design, clinical input and interpretation of data, provided critical comments, edited the manuscript, and approved its final version. RDN provided advice and expertise for study design, clinical input and interpretation of data, provided critical comments, edited the manuscript, and approved its final version. KL created the natural language annotation tool and extracted free text data, assisted with interpretation of data, provided critical comments, edited the manuscript, and approved its final version. CT provided advice and expertise for study design, analytic methods and interpretation of data, provided critical comments, edited the manuscript, and approved its final version. MAlA provided advice and expertise for study design, clinical input and interpretation of data, provided critical comments, edited the manuscript, and approved its final version. EAS provided advice and expertise for study design, clinical input and interpretation of data, provided critical comments, edited the manuscript, and approved its final version. GT supported implementation of the natural language annotation tool and extracted free text data, assisted with interpretation of data, provided critical comments, edited the manuscript, and approved its final version. FF provided advice and expertise for study design, clinical input and interpretation of data, provided critical comments, edited the manuscript, and approved its final version. MT was the Principal Investigator for the study and is its guarantor, designed the study and supervised its execution, provided clinical guidance, interpreted data, wrote the manuscript, edited the manuscript, and approved its final version.

Funding: This research was funded by the Gordon and Betty Moore Foundation through Grant GBMF8837 to the University of Washington. This research is linked to the CanTest Collaborative, which is funded by Cancer Research UK [C8640/A23385], of which FMW is Director and RDN and MT are Associate Directors. This research was supported by the Cancer Surveillance System of the Fred Hutchinson Cancer Research Center, which is funded by Contract Numbers HHSN261201800004I & N01 PC-2018-00004 from the Surveillance, Epidemiology and End Results (SEER) Program of the National Cancer Institute with additional support from the Fred Hutchinson Cancer Research Center and the State of Washington.

For peer review only - http://bmjopen.bmj.com/site/about/guidelines.xhtml

For peer review only

The views expressed are those of the authors and do not necessarily represent the official position of the National Cancer Institute, the National Institute of Health, or Department of Health and Human Services.

Informed Consent Statement: Not applicable.

Data Sharing Statement: Fully anonymized data may be available on reasonable request to the corresponding author, once appropriate data sharing and ethics approvals have been obtained.

Acknowledgments: We would like to thank the patients and clinicians at University of Washington Medicine, and the members of our TEP.

Conflicts of Interest: The authors declare no conflict of interest.

Ethical Approval Statement: The study was conducted according to the guidelines of the Declaration of Helsinki, and was classified as Exempt by the University of Washington Human Subjects Division (STUDY 000013191).

Figure 1. Flow chart of case and control selection

Figure 2: Multivariable analysis of symptoms or signs of cases compared to controls with symptom and sign data excluded from 1, 3, 6, and 12 months prior to diagnosis/index date

References

1. Centers for Diseases Control and Prevention. Leading cancer cases and deaths, all Races/Ethnicities, male and female, 2018. Accessed January 16, 2022. https://gis.cdc.gov/grasp/USCS/DataViz.html

2. American Lung Association. State of Lung Cancer 2020 Report. Published online 2020:15.

3. Fedewa SA, Bandi P, Smith RA, Silvestri GA, Jemal A. Lung Cancer Screening Rates During the COVID-19 Pandemic. *Chest*. Published online July 2021:S0012369221013647. doi:10.1016/j.chest.2021.07.030

For peer review only - http://bmjopen.bmj.com/site/about/guidelines.xhtml

4. The National Lung Screening Trial Research Team. Reduced Lung-Cancer Mortality with Low-Dose Computed Tomographic Screening. *N Engl J Med*. 2011;365(5):395-409. doi:10.1056/NEJMoa1102873

5. Hamilton W, Peters TJ, Round A, Sharp D. What are the clinical features of lung cancer before the diagnosis is made? A population based case-control study. *Thorax*. 2005;60(12):1059-1065. doi:10.1136/thx.2005.045880

6. Walter FM, Rubin G, Bankhead C, et al. Symptoms and other factors associated with time to diagnosis and stage of lung cancer: a prospective cohort study. *Br J Cancer*. 2015;112(S1):S6-S13. doi:10.1038/bjc.2015.30

7. Koo MM, Hamilton W, Walter FM, Rubin GP, Lyratzopoulos G. Symptom Signatures and Diagnostic Timeliness in Cancer Patients: A Review of Current Evidence. *Neoplasia*. 2018;20(2):165-174. doi:10.1016/j.neo.2017.11.005

8. Nadpara PA, Madhavan SS, Tworek C, Sambamoorthi U, Hendryx M, Almubarak M. Guideline-concordant lung cancer care and associated health outcomes among elderly patients in the United States. *J Geriatr Oncol*. 2015;6(2):101-110. doi:10.1016/j.jgo.2015.01.001

9. Cancer Statistics Review, 1975-2018 - SEER Statistics. Accessed January 16, 2022. https://seer.cancer.gov/csr/1975_2018/

10. Elixhauser A, Steiner C, Harris DR, Coffey RM. Comorbidity Measures for Use with Administrative Data: *Med Care*. 1998;36(1):8-27. doi:10.1097/00005650-199801000-00004

11. van Walraven C, Austin PC, Jennings A, Quan H, Forster AJ. A Modification of the Elixhauser Comorbidity Measures Into a Point System for Hospital Death Using Administrative Data. *Med Care*. 2009;47(6):626-633. doi:10.1097/MLR.0b013e31819432e5

12. Thompson NR, Fan Y, Dalton JE, et al. A New Elixhauser-based Comorbidity Summary Measure to Predict In-Hospital Mortality. *Med Care*. 2015;53(4):374-379. doi:10.1097/MLR.0000000000000326

13. Hippisley-Cox J, Coupland C. Symptoms and risk factors to identify men with suspected cancer in primary care: derivation and validation of an algorithm. *Br J Gen Pract*. 2013;63(606):e1-e10. doi:10.3399/bjgp13X660724

14. Gould MK, Ghaus SJ, Olsson JK, Schultz EM. Timeliness of Care in Veterans With Non-small Cell Lung Cancer. *Chest*. 2008;133(5):1167-1173. doi:10.1378/chest.07-2654

15. Ades AE, Biswas M, Welton NJ, Hamilton W. Symptom lead time distribution in lung cancer: natural history and prospects for early diagnosis. *Int J Epidemiol*. 2014;43(6):1865-1873. doi:10.1093/ije/dyu174

For peer review only

16. Redaniel MT, Martin RM, Ridd MJ, Wade J, Jeffreys M. Diagnostic Intervals and Its Association with Breast, Prostate, Lung and Colorectal Cancer Survival in England: Historical Cohort Study Using the Clinical Practice Research Datalink. Metze K, ed. *PLOS ONE*. 2015;10(5):e0126608. doi:10.1371/journal.pone.0126608

17. Corner J, Hopkinson J, Fitzsimmons D, Barclay S, Muers M. Is late diagnosis of lung cancer inevitable? Interview study of patients' recollections of symptoms before diagnosis. *Thorax*. 2005;60(4):314-319. doi:10.1136/thx.2004.029264

18. Tod AM, Craven J, Allmark P. Diagnostic delay in lung cancer: a qualitative study: Diagnostic delay in lung cancer. *J Adv Nurs*. 2008;61(3):336-343. doi:10.1111/j.1365-2648.2007.04542.x

19. Lybarger K, Ostendorf M, Thompson M, Yetisgen M. Extracting COVID-19 diagnoses and symptoms from clinical text: A new annotated corpus and neural event extraction framework. *J Biomed Inform*. 2021;117:103761. doi:10.1016/j.jbi.2021.103761

20. Turner G, Chang J, Dorvall N, et al. Domain Adaptation of a Deep Learning Symptom Extractor for Different Patient Populations and Clinical Settings. In: *AMIA 2022 Informatics Summit*.

21. Paszke A, Gross S, Massa F, et al. PyTorch: An Imperative Style, High-Performance Deep Learning Library. Published online December 3, 2019. Accessed February 28, 2023. http://arxiv.org/abs/1912.01703

22. Wolf T, Debut L, Sanh V, et al. Transformers: State-of-the-Art Natural Language Processing. In: *Proceedings of the 2020 Conference on Empirical Methods in Natural Language Processing: System Demonstrations*. Association for Computational Linguistics; 2020:38-45. doi:10.18653/v1/2020.emnlp-demos.6

23. von Elm E, Altman DG, Egger M, Pocock SJ, Gøtzsche PC, Vandenbroucke JP. The Strengthening the Reporting of Observational Studies in Epidemiology (STROBE) Statement: Guidelines for reporting observational studies. *Int J Surg*. 2014;12(12):1495-1499. doi:10.1016/j.ijsu.2014.07.013

24. Ellis PM, Vandermeer R. Delays in the diagnosis of lung cancer. *J Thorac Dis*. 2011;3(3):183-188. doi:10.3978/j.issn.2072-1439.2011.01.01

25. Koyi H, Hillerdal G, Brandén E. Patient's and doctors' delays in the diagnosis of chest tumors. *Lung Cancer*. 2002;35(1):53-57. doi:10.1016/S0169-5002(01)00293-8

26. Al Achkar M, Zigman Suchsland M, Walter FM, Neal RD, Goulart BHL, Thompson MJ. Experiences along the diagnostic pathway for patients with advanced lung cancer in the USA: a qualitative study. *BMJ Open*. 2021;11(4):e045056. doi:10.1136/bmjopen-2020-045056

For peer review only

27. Corner J, Hopkinson J, Roffe L. Experience of health changes and reasons for delay in seeking care: a UK study of the months prior to the diagnosis of lung cancer. *Soc Sci Med 1982*. 2006;62(6):1381-1391. doi:10.1016/j.socscimed.2005.08.012

28. Nicholson BD, Aveyard P, Koshiaris C, et al. Combining simple blood tests to identify primary care patients with unexpected weight loss for cancer investigation: Clinical risk score development, internal validation, and net benefit analysis. *PLOS Med*. 2021;18(8):e1003728. doi:10.1371/journal.pmed.1003728

29. Sarma EA, Kobrin SC, Thompson MJ. A Proposal to Improve the Early Diagnosis of Symptomatic Cancers in the United States. *Cancer Prev Res (Phila Pa)*. 2020;13(9):715-720. doi:10.1158/1940-6207.CAPR-20-0115

For peer review only

Figure 1. Flow chart of case and control selection

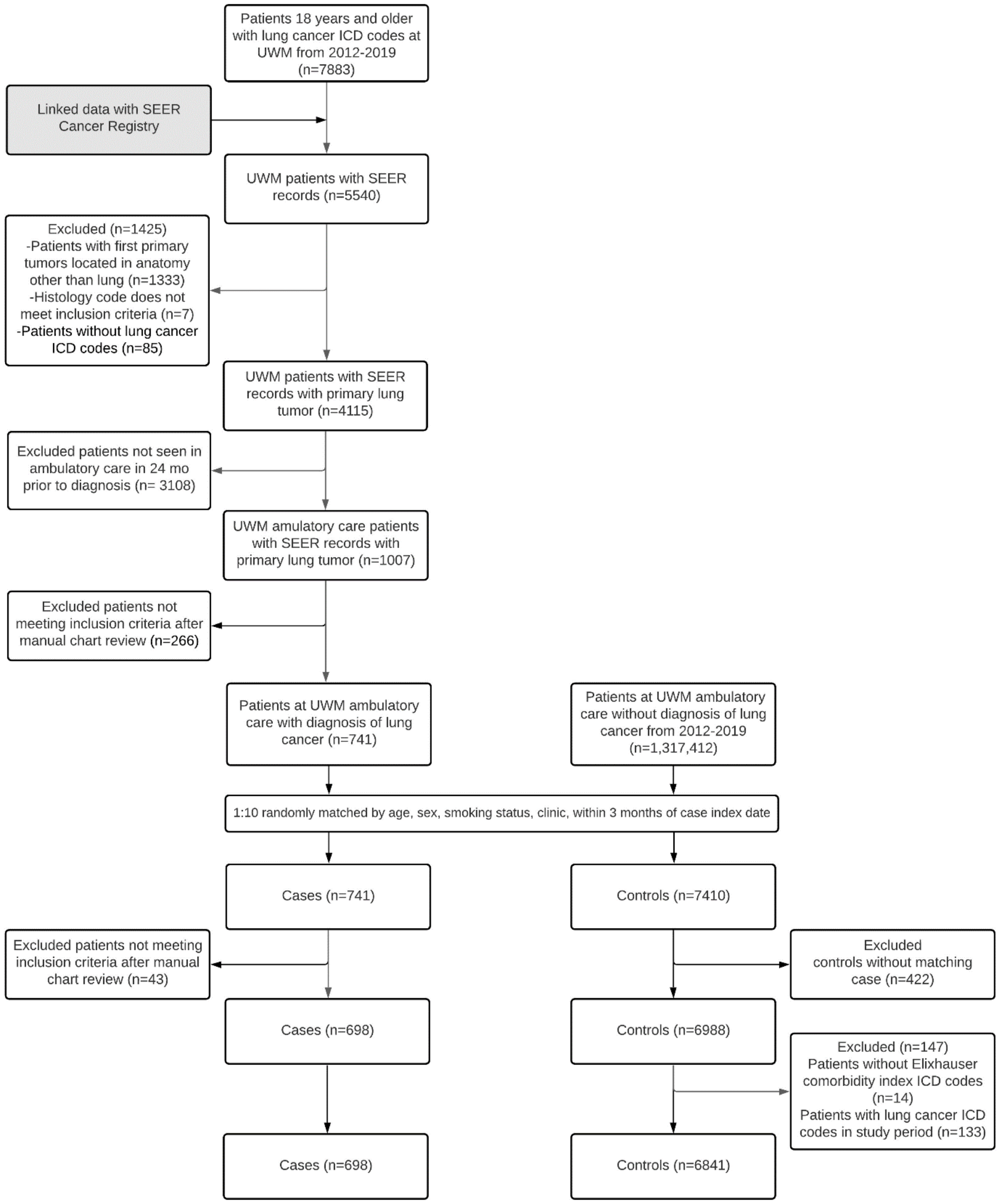

Figure 2: Multivariable analysis of symptoms or signs of cases compared to controls with symptom and sign data excluded from 1, 3, 6, and 12 months prior to diagnosis/index date

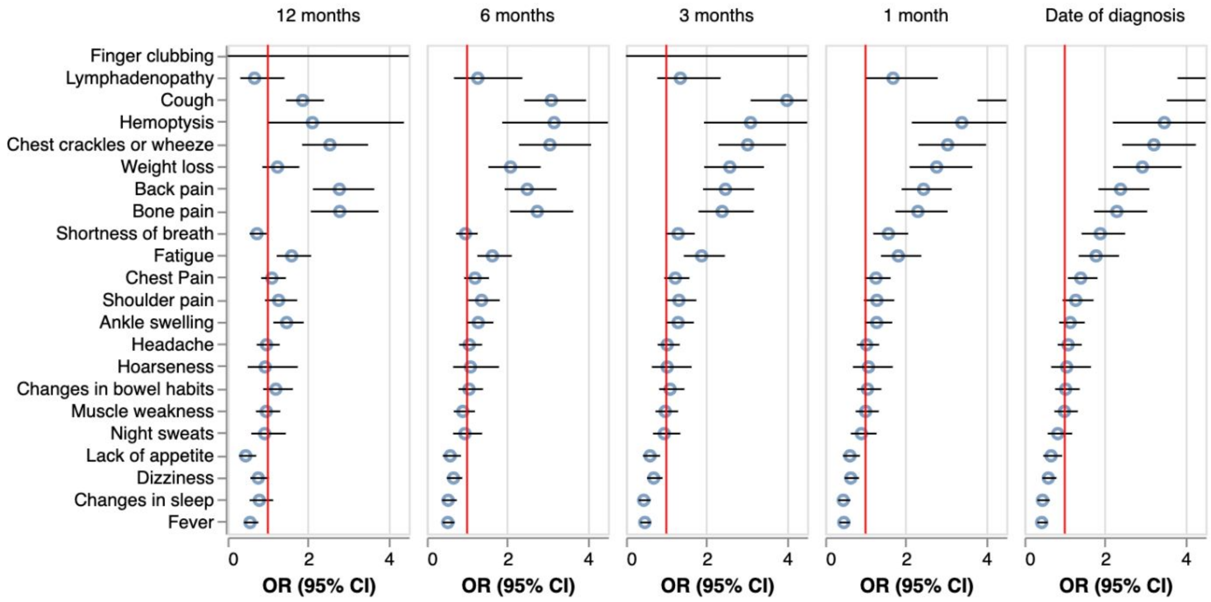

Note: Mutual adjustment of all symptoms and signs in using a conditional logistic regression model stratified by time prior to date of diagnosis. Models additionally adjusted for comorbidities using van Walraven weighted score. For the complete set of results, see Appendix 5.

Symptoms and signs of lung cancer prior to diagnosis: Comparative study using natural language processing of electronic health records

Appendix 1. Diagnostic codes used to identify cases of lung cancer

ICD 9: 162.2 – 162.9

- 162.2 - Malignant neoplasm of main bronchus
- 162.3 - Malignant neoplasm of upper lobe, bronchus or lung
- 162.4 - Malignant neoplasm of middle lobe, bronchus or lung
- 162.5 - Malignant neoplasm of lower lobe, bronchus or lung
- 162.8 - Malignant neoplasm of other parts of bronchus or lung
- 162.9 - Malignant neoplasm of bronchus and lung, unspecified

ICD 10: C34.0 – C34.9

- C34.0 - Malignant neoplasm of main bronchus
- C34.00 - Malignant neoplasm of unspecified main bronchus
- C34.01 - Malignant neoplasm of right main bronchus
- C34.02 - Malignant neoplasm of left main bronchus
- C34.1 - Malignant neoplasm of upper lobe, bronchus or lung
- C34.10 - Malignant neoplasm of upper lobe, unspecified bronchus or lung
- C34.11 - Malignant neoplasm of upper lobe, right bronchus or lung
- C34.12 - Malignant neoplasm of upper lobe, left bronchus or lung
- C34.2 - Malignant neoplasm of middle lobe, bronchus or lung
- C34.3 - Malignant neoplasm of lower lobe, bronchus or lung
- C34.30 - Malignant neoplasm of lower lobe, unspecified bronchus or lung
- C34.31 - Malignant neoplasm of lower lobe, right bronchus or lung
- C34.32 - Malignant neoplasm of lower lobe, left bronchus or lung
- C34.8 - Malignant neoplasm of overlapping sites of bronchus and lung
- C34.80 - Malignant neoplasm of overlapping sites of unspecified bronchus and lung
- C34.81 - Malignant neoplasm of overlapping sites of right bronchus and lung
- C34.82 - Malignant neoplasm of overlapping sites of left bronchus and lung
- C34.9 - Malignant neoplasm of unspecified part of bronchus or lung
- C34.90 - Malignant neoplasm of unspecified part of unspecified bronchus or lung
- C34.91 - Malignant neoplasm of unspecified part of right bronchus or lung
- C34.92 - Malignant neoplasm of unspecified part of left bronchus or lung

Excluded ICD Diagnostic Codes

- ICD-9: 162.0
- ICD-10: C33

Excluded Histology codes

- Mesothelioma: 9050-9055
- Kaposi Sarcoma: 9140
- Lymphoma/leukemia: M9590-M9992

For peer review only

Appendix 2. Symptoms and signs Identified in peer-reviewed literature previously associated with lung cancer in primary care populations

Symptom or sign	ICD 9 code(s)	ICD10 code(s)	References
Ankle swelling	782.3	R60.9	[1]Ellis (2011)
Back pain	724.1	M54.6	[1]Ellis (2011) [2]Molassiotis (2010)
Bone pain	733.9	M85.80	[3]Gould (2008) [4]Nadpara (2015)
Changes in bowel habits	787.99	R19.4	[5]Corner (2005)
Changes in sleep	780.50	G47.9	[5]Corner (2005)
Chest Pain	786.5<br>786.50<br>786.51<br>786.52<br>786.59	R07.9<br>R07.81	[1]Ellis (2011) [4]Nadpara (2015) [5]Corner (2005) [6]Chowienczyk, Hamilton (2020) [7]Walter (2015) [8]Hamilton (2005) [9]Ades (2014) [10]Redaniel (2015) [11]Tod (2008) [12]Mitchell (2013)
Chest crackles or wheeze	786.7	R09.89	[10]Redaniel (2015)
Cough	786.2<br>491.0	R05	[1]Ellis (2011) [2]Molassiotis (2010) [4]Nadpara (2015) [5]Corner (2005) [6]Chowienczyk, Hamilton (2020) [7]Walter (2015) [9]Ades (2014) [10]Redaniel (2015) [11]Tod (2008) [12]Mitchell (2013) [13]Menon (2019)
Dizziness	780.4	R42	[2]Molassiotis (2010)
Fatigue/tiredness	780.79	R53.81<br>R53.8<br>R53.83<br>R53.1	[1]Ellis (2011) [2]Molassiotis (2010) [5]Corner (2005) [6]Chowienczyk, Hamilton (2020) [7]Walter (2015) [8]Hamilton (2005) [10]Redaniel (2015) [11]Tod (2008) [13]Menon (2019)
Fever	780.6<br>780.60	R50.9	[4]Nadpara (2015)
Finger clubbing	781.5	R68.3	[4]Nadpara (2015) [8]Hamilton (2005) [10]Redaniel (2015)
Headache	784.0	R51	[1]Ellis (2011)
Hemoptysis	786.3<br>786.30<br>786.39	R04.2	[1]Ellis (2011) [4]Nadpara (2015) [5]Corner [6]Chowienczyk, Hamilton (2020) [7]Walter (2015) [8]Hamilton (2005) [10]Redaniel (2015) (2005) [11]Tod (2008) [12]Mitchell (2013) [13]Menon (2019) [14]Hippisley-Cox (2011)

For peer review only

Hoarseness	784.49<br>784.42	R49.8<br>R49.0	[1]Ellis (2011) [2]Molassiotis (2010) [7]Walter (2015) [10]Redaniel (2015) [11]Tod (2008) [12]Mitchell (2013)
Lack of appetite	783	R63.0	[1]Ellis (2011) [2]Molassiotis (2010) [5]Corner (2005) [6]Chowienczyk, Hamilton (2020) [7]Walter (2015) [8]Hamilton (2005) [13]Menon (2019)
Lympadenopathy	785.6	R59.9	[10]Redaniel (2015) [12]Mitchell (2013)
Muscle weakness	728.87	M62.81	[4]Nadpara (2015) [12]Mitchell (2013)
Night sweats	780.8	R61	[3]Gould (2008) [5]Corner (2005)
Shortness of breath	786.05<br>786.0<br>786.9	R06.02<br>R06.00<br>R06.09	[1]Ellis (2011) [2]Molassiotis (2010) [4]Nadpara (2015) [5]Corner (2005) [6]Chowienczyk, Hamilton (2020) [7]Walter (2015) [8]Hamilton (2005) [10]Redaniel (2015) [12]Mitchell (2013) [13]Menon (2019)
Shoulder pain	719.41	M25.511<br>M25.512<br>M25.519	[10]Redaniel (2015) [12]Mitchell (2013)
Weight loss	783.21	R63.4	[1]Ellis (2011) [4]Nadpara (2015) [5]Corner (2005) [6]Chowienczyk, Hamilton (2020) [7]Walter (2015) [8]Hamilton (2005) [10]Redaniel (2015) [11]Tod (2008) [12]Mitchell (2013)
Wheezing and stridor	786.07<br>786.1	R06.2<br>R06.1	[4]Nadpara (2015) [10]Redaniel (2015)

1. Ellis PM, Vandermeer R. Delays in the diagnosis of lung cancer. *J Thorac Dis*. 2011;3(3):183-188. doi:10.3978/j.issn.2072-1439.2011.01.01

2. Molassiotis A, Wilson B, Brunton L, Chandler C. Mapping patients' experiences from initial change in health to cancer diagnosis: a qualitative exploration of patient and system factors mediating this process. *Eur J Cancer Care (Engl)*. 2010;19(1):98-109. doi:10.1111/j.1365-2354.2008.01020.x

3. Gould MK, Ghaus SJ, Olsson JK, Schultz EM. Timeliness of Care in Veterans With Non-small Cell Lung Cancer. *Chest*. 2008;133(5):1167-1173. doi:10.1378/chest.07-2654

4. Nadpara PA, Madhavan SS, Tworek C, Sambamoorthi U, Hendryx M, Almubarak M. Guideline-concordant lung cancer care and associated health outcomes among elderly patients in the United States. *J Geriatr Oncol*. 2015;6(2):101-110. doi:10.1016/j.jgo.2015.01.001

5. Corner J, Hopkinson J, Fitzsimmons D, Barclay S, Muers M. Is late diagnosis of lung cancer inevitable? Interview study of patients' recollections of symptoms before diagnosis. *Thorax*. 2005;60(4):314-319. doi:10.1136/thx.2004.029264

For peer review only

6. Chowienczyk S, Price S, Hamilton W. Changes in the presenting symptoms of lung cancer from 2000–2017: a serial cross-sectional study of observational records in UK primary care. *Br J Gen Pract*. 2020;70(692):e193-e199. doi:10.3399/bjgp20X708137

7. Walter FM, Rubin G, Bankhead C, et al. Symptoms and other factors associated with time to diagnosis and stage of lung cancer: a prospective cohort study. *Br J Cancer*. 2015;112(S1):S6-S13. doi:10.1038/bjc.2015.30

8. Hamilton W. What are the clinical features of lung cancer before the diagnosis is made? A population based case-control study. *Thorax*. 2005;60(12):1059-1065. doi:10.1136/thx.2005.045880

9. Ades AE, Biswas M, Welton NJ, Hamilton W. Symptom lead time distribution in lung cancer: natural history and prospects for early diagnosis. *Int J Epidemiol*. 2014;43(6):1865-1873. doi:10.1093/ije/dyu174

10. Redaniel MT, Martin RM, Ridd MJ, Wade J, Jeffreys M. Diagnostic Intervals and Its Association with Breast, Prostate, Lung and Colorectal Cancer Survival in England: Historical Cohort Study Using the Clinical Practice Research Datalink. Metze K, ed. *PLOS ONE*. 2015;10(5):e0126608. doi:10.1371/journal.pone.0126608

11. Tod AM, Craven J, Allmark P. Diagnostic delay in lung cancer: a qualitative study: Diagnostic delay in lung cancer. *J Adv Nurs*. 2008;61(3):336-343. doi:10.1111/j.1365-2648.2007.04542.x

12. Mitchell ED, Rubin G, Macleod U. Understanding diagnosis of lung cancer in primary care: qualitative synthesis of significant event audit reports. *Br J Gen Pract*. 2013;63(606):e37-e46. doi:10.3399/bjgp13X660760

13. Menon U, Vedsted P, Zalounina Falborg A, et al. Time intervals and routes to diagnosis for lung cancer in 10 jurisdictions: cross-sectional study findings from the International Cancer Benchmarking Partnership (ICBP). *BMJ Open*. 2019;9(11):e025895. doi:10.1136/bmjopen-2018-025895

For peer review only

Appendix 3. Span-based Event Extractor

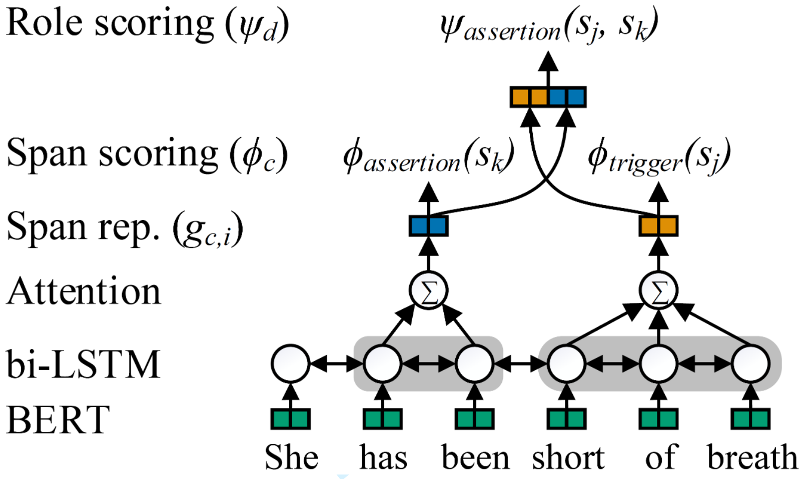

For peer review only

Appendix 4. Comparison of the number of patients with symptoms and signs extracted from the electronic medical record of cases or controls from coded fields versus free-text data using natural language processing (NLP)

Symptom or sign	Identified from NLP (% of patients)	Identified from coded data (% of patients)	Identified from either coded data or NLP (% of patients)	NLP adds (NLP adds n/coded or NLP n)
Cough	1700 (22.6%)	1139 (15.1%)	2227 (29.5%)	1088 (48.9%)
Shortness of breath	1580 (21.0%)	1111 (14.7%)	2128 (28.2%)	1017 (47.8%)
Chest Pain	1241 (16.5%)	981 (13.0%)	1804 (23.9%)	823 (45.6%)
Fatigue	1489 (19.8%)	959 (12.7%)	2063 (27.4%)	1104 (53.5%)
Shoulder pain	513 (6.8%)	594 (7.9%)	893 (11.9%)	299 (33.5%)
Dizziness	1331 (17.7%)	536 (7.1%)	1618 (21.5%)	1082 (66.9%)
Ankle swelling	2081 (27.6%)	509 (6.8%)	2285 (30.3%)	1776 (77.7%)
Headache	1281 (17.0%)	415 (5.5%)	1509 (20.0%)	1094 (72.5%)
Weight loss	646 (8.6%)	328 (4.4%)	830 (11.0%)	502 (60.5%)
Fever	1517 (20.1%)	252 (3.3%)	1656 (22.0%)	1404 (84.8%)
Chest crackles or wheeze	834 (11.1%)	242 (3.2%)	972 (12.9%)	730 (75.1%)
Lympadenopathy	52 (0.7%)	223 (3.0%)	256 (3.4%)	33 (12.9%)
Bone pain	829 (11.0%)	216 (2.9%)	995 (13.2%)	779 (78.3%)
Muscle weakness	1327 (17.6%)	205 (2.7%)	1436 (19.1%)	1231 (85.7%)
Back pain	1220 (16.2%)	154 (2.0%)	1296 (17.2%)	1142 (88.1%)
Changes in sleep	662 (8.8%)	137 (1.8%)	765 (10.2%)	628 (82.1%)
Hoarseness	130 (1.7%)	118 (1.6%)	200 (2.7%)	82 (41.0%)
Hemoptysis	133 (1.8%)	94 (1.3%)	182 (2.4%)	88 (48.4%)
Night sweats	480 (6.4%)	72 (1.0%)	521 (6.9%)	449 (86.2%)
Lack of appetite	626 (8.3%)	59 (0.8%)	653 (8.7%)	594 (91.0%)
Change in bowel habits	1465 (19.4%)	59 (0.8%)	1491 (19.8%)	1432 (96.0%)
Finger clubbing	41 (0.5%)	1 (0.0%)	41 (0.5%)	40 (97.6%)

For peer review only

Appendix 5. Multivariable analysis of symptoms or signs of cases compared to controls at 1, 3, 6 and 12 months prior to diagnosis/index date

Symptom or sign	12 months OR	6 months OR	3 months OR	1 month OR	At diagnosis OR
**Finger clubbing**	>1,000 (0.0 - >1,000)	>1,000 (0.0 - >1,000)	>1,000 (0.0 - >1,000)	60.7 (10.6 - 348.7)***	50.1 (8.9 - 283.3)***
**Lymphadenopathy**	0.7 (0.3 - 1.4)	1.3 (0.7 - 2.4)	1.3 (0.8 - 2.3)	1.7 (1.0 - 2.8)*	5.8 (3.8 - 8.8)***
**Cough**	1.9 (1.5 - 2.4)***	3.1 (2.4 - 4.0)***	4.0 (3.1 - 5.2)***	5.0 (3.8 - 6.5)***	4.7 (3.5 - 6.3)***
**Hemoptysis**	2.1 (1.0 - 4.4)*	3.2 (1.9 - 5.3)***	3.1 (1.9 - 4.9)***	3.4 (2.2 - 5.4)***	3.5 (2.2 - 5.5)***
**Chest crackles or wheeze**	2.5 (1.9 - 3.5)***	3.1 (2.3 - 4.1)***	3.0 (2.3 - 4.0)***	3.0 (2.3 - 4.0)***	3.2 (2.4 - 4.3)***
**Weight loss**	1.2 (0.9 - 1.8)	2.1 (1.5 - 2.8)***	2.6 (1.9 - 3.4)***	2.8 (2.1 - 3.7)***	2.9 (2.2 - 3.9)***
**Back pain**	2.8 (2.1 - 3.6)***	2.5 (1.9 - 3.2)***	2.5 (1.9 - 3.2)***	2.4 (1.9 - 3.1)***	2.4 (1.8 - 3.1)***
**Bone pain**	2.8 (2.1 - 3.7)***	2.7 (2.1 - 3.6)***	2.4 (1.8 - 3.2)***	2.3 (1.7 - 3.0)***	2.3 (1.7 - 3.0)***
**Shortness of breath**	0.7 (0.5 - 1.0)*	1.0 (0.7 - 1.3)	1.3 (1.0 - 1.7)	1.6 (1.2 - 2.1)**	1.9 (1.4 - 2.5)***
**Fatigue**	1.6 (1.2 - 2.1)***	1.6 (1.3 - 2.1)***	1.9 (1.4 - 2.5)***	1.8 (1.4 - 2.4)***	1.8 (1.3 - 2.3)***
**Chest Pain**	1.1 (0.8 - 1.4)	1.2 (0.9 - 1.5)	1.2 (1.0 - 1.6)	1.3 (1.0 - 1.6)	1.4 (1.1 - 1.8)*
**Shoulder pain**	1.3 (0.9 - 1.7)	1.4 (1.0 - 1.8)*	1.3 (1.0 - 1.7)	1.3 (1.0 - 1.7)	1.3 (0.9 - 1.7)
**Ankle swelling**	1.5 (1.1 - 1.9)**	1.3 (1.0 - 1.7)	1.3 (1.0 - 1.7)	1.3 (1.0 - 1.7)	1.1 (0.9 - 1.5)
**Headache**	1.0 (0.7 - 1.3)	1.1 (0.8 - 1.4)	1.0 (0.8 - 1.3)	1.0 (0.8 - 1.3)	1.1 (0.8 - 1.4)
**Hoarseness**	0.9 (0.5 - 1.7)	1.1 (0.7 - 1.8)	1.0 (0.6 - 1.6)	1.1 (0.7 - 1.7)	1.0 (0.7 - 1.7)
**Changes in bowel habits**	1.2 (0.9 - 1.6)	1.0 (0.8 - 1.4)	1.1 (0.8 - 1.5)	1.0 (0.8 - 1.4)	1.0 (0.8 - 1.4)
**Muscle weakness**	1.0 (0.7 - 1.3)	0.9 (0.7 - 1.2)	1.0 (0.7 - 1.3)	1.0 (0.8 - 1.3)	1.0 (0.7 - 1.3)
**Night sweats**	0.9 (0.6 - 1.4)	0.9 (0.7 - 1.4)	0.9 (0.7 - 1.3)	0.9 (0.6 - 1.3)	0.8 (0.6 - 1.2)
**Lack of appetite**	0.5 (0.3 - 0.7)***	0.6 (0.4 - 0.8)**	0.6 (0.4 - 0.8)**	0.6 (0.4 - 0.9)**	0.7 (0.5 - 0.9)*
**Dizziness**	0.8 (0.6 - 1.0)	0.7 (0.5 - 0.9)**	0.7 (0.5 - 0.9)**	0.6 (0.5 - 0.8)**	0.6 (0.4 - 0.8)***
**Changes in sleep**	0.8 (0.5 - 1.1)	0.5 (0.4 - 0.7)***	0.4 (0.3 - 0.6)***	0.4 (0.3 - 0.6)***	0.4 (0.3 - 0.6)***
**Fever**	0.6 (0.4 - 0.8)***	0.5 (0.4 - 0.7)***	0.5 (0.4 - 0.6)***	0.5 (0.3 - 0.6)***	0.4 (0.3 - 0.6)***

Note: Models adjusted for comorbidities using van Walraven weighted score. Confidence intervals for significant ORs do not incorporate 1.0 due to rounding.

* p<0.05

** p<0.01

*** p<0.001

For peer review only

Appendix 6. Frequency of symptoms and signs in cases and controls with and without chronic respiratory disease

Symptom or sign	Chronic respiratory disease		No chronic respiratory disease	
	Control (n=1252)	Case (n=353)	Control (n=5589)	Case (n=345)
Cough	636 (50.8%)	312 (88.4%)	1018 (18.2%)	261 (75.7%)
Shortness of breath	623 (49.8%)	307 (87.0%)	990 (17.7%)	208 (60.3%)
Fatigue	459 (36.7%)	266 (75.4%)	1128 (20.2%)	210 (60.9%)
Ankle swelling	516 (41.2%)	250 (70.8%)	1322 (23.7%)	197 (57.1%)
Chest Pain	439 (35.1%)	228 (64.6%)	962 (17.2%)	175 (50.7%)
Chest crackles or wheeze	307 (24.5%)	268 (75.9%)	268 (4.8%)	129 (37.4%)
Back pain	278 (22.2%)	191 (54.1%)	668 (12.0%)	159 (46.1%)
Changes in bowel habits	337 (26.9%)	195 (55.2%)	818 (14.6%)	141 (40.9%)
Muscle weakness	327 (26.1%)	177 (50.1%)	775 (13.9%)	157 (45.5%)
Fever	433 (34.6%)	177 (50.1%)	901 (16.1%)	145 (42.0%)
Weight loss	165 (13.2%)	191 (54.1%)	357 (6.4%)	117 (33.9%)
Headache	324 (25.9%)	175 (49.6%)	881 (15.8%)	129 (37.4%)
Dizziness	366 (29.2%)	174 (49.3%)	953 (17.1%)	125 (36.2%)
Bone pain	207 (16.5%)	141 (39.9%)	518 (9.3%)	129 (37.4%)
Lack of appetite	142 (11.3%)	116 (32.9%)	315 (5.6%)	80 (23.2%)
Shoulder pain	200 (16.0%)	92 (26.1%)	513 (9.2%)	88 (25.5%)
Lymphadenopathy	35 (2.8%)	79 (22.4%)	70 (1.3%)	72 (20.9%)
Night sweats	113 (9.0%)	89 (25.2%)	258 (4.6%)	61 (17.7%)
Changes in sleep	178 (14.2%)	90 (25.5%)	453 (8.1%)	44 (12.8%)
Hemoptysis	31 (2.5%)	72 (20.4%)	36 (0.6%)	43 (12.5%)
Hoarseness	55 (4.4%)	45 (12.7%)	78 (1.4%)	22 (6.4%)
Finger clubbing	1 (0.1%)	28 (7.9%)	1 (0.0%)	11 (3.2%)

For peer review only

Appendix 7. Multivariate analysis of symptoms and signs in patients with and without chronic respiratory disease

Symptom or sign	Chronic respiratory disease			No chronic respiratory disease		
	Univariate Odds ratio (95%CI)	Multivariate Odds ratio (95%CI)	Multivariate P value	Univariate Odds ratio (95%CI)	Multivariate Odds ratio (95%CI)	Multivariate P value
Finger clubbing	47.3 (6.1 - 364.5)	17.8 (1.3 - 247.1)	0.0322	>1,000 (0.0 - >1,000)	267.7 (0.1 - >1,000)	0.1783
Chest crackles or wheeze	9.4 (6.3 - 14.2)*	4.9 (2.6 - 9.0)	<0.0001	9.8 (7.0 - 13.9)*	3.2 (2.0 - 5.2)	<0.0001
Hemoptysis	12.5 (6.2 - 25.3)*	4.4 (1.7 - 11.5)	0.0028	20.3 (10.2 - 40.5)*	3.8 (1.5 - 9.8)	0.0049
Weight loss	7.1 (4.7 - 10.5)*	4.0 (2.2 - 7.4)	<0.0001	3.8 (2.8 - 5.3)*	1.6 (1.0 - 2.5)	0.0643
Lympadenopathy	7.1 (3.9 - 13.0)*	3.3 (1.3 - 7.9)	0.0089	12.0 (7.2 - 19.9)*	8.5 (4.3 - 17.0)	<0.0001
Fatigue	5.2 (3.6 - 7.6)*	2.9 (1.6 - 5.5)	0.0008	4.2 (3.2 - 5.6)*	1.7 (1.1 - 2.6)	0.0128
Back pain	4.6 (3.2 - 6.6)*	2.4 (1.4 - 4.1)	0.0014	4.8 (3.6 - 6.4)*	2.1 (1.4 - 3.2)	0.0003
Cough	6.5 (4.2 - 10.2)*	2.2 (1.1 - 4.3)	0.0189	12.2 (9.0 - 16.6)*	6.3 (4.2 - 9.3)	<0.0001
Bone pain	3.8 (2.6 - 5.5)*	2.1 (1.1 - 4.0)	0.0168	5.3 (3.9 - 7.2)*	2.5 (1.6 - 3.9)	0.0001
Shortness of breath	6.5 (4.1 - 10.3)*	1.6 (0.8 - 3.2)	0.1688	5.1 (3.9 - 6.7)*	1.9 (1.3 - 2.9)	0.0024
Changes in bowel habits	2.7 (2.0 - 3.8)*	1.3 (0.7 - 2.3)	0.4474	2.5 (1.9 - 3.4)*	0.9 (0.6 - 1.4)	0.7286
Night sweats	3.1 (2.1 - 4.7)*	1.2 (0.6 - 2.4)	0.5393	3.8 (2.6 - 5.7)*	0.9 (0.5 - 1.7)	0.8542
Ankle swelling	2.8 (2.0 - 3.9)*	1.1 (0.6 - 2.0)	0.6696	3.1 (2.4 - 4.0)*	1.2 (0.8 - 1.8)	0.3121
Shoulder pain	1.6 (1.1 - 2.4)	1.1 (0.6 - 2.0)	0.7589	2.9 (2.1 - 4.0)*	1.6 (1.0 - 2.5)	0.0484
Hoarseness	2.5 (1.4 - 4.4)	1.0 (0.5 - 2.3)	0.9617	4.1 (2.2 - 7.7)*	0.9 (0.4 - 2.2)	0.8729
Headache	2.5 (1.9 - 3.5)*	0.9 (0.5 - 1.7)	0.8551	2.2 (1.7 - 2.9)*	1.0 (0.7 - 1.6)	0.8319
Chest Pain	2.6 (1.9 - 3.6)*	0.9 (0.5 - 1.6)	0.7953	3.7 (2.8 - 4.8)*	1.5 (1.0 - 2.2)	0.0494
Muscle weakness	2.3 (1.7 - 3.2)*	0.9 (0.5 - 1.7)	0.7901	3.1 (2.3 - 4.1)*	1.1 (0.7 - 1.7)	0.6809
Dizziness	2.3 (1.7 - 3.3)*	0.9 (0.5 - 1.6)	0.7450	1.8 (1.3 - 2.4)*	0.5 (0.3 - 0.8)	0.0027
Lack of appetite	2.6 (1.8 - 3.8)*	0.5 (0.3 - 1.0)	0.0667	1.8 (1.3 - 2.6)	0.5 (0.3 - 0.9)	0.0122
Changes in sleep	1.6 (1.1 - 2.3)	0.5 (0.3 - 0.9)	0.0233	1.1 (0.7 - 1.6)	0.3 (0.2 - 0.6)	0.0004
Fever	1.6 (1.2 - 2.2)	0.3 (0.2 - 0.6)	0.0003	2.5 (1.9 - 3.3)*	0.6 (0.4 - 0.9)	0.0229

Note: Models adjusted for comorbidities using van Walraven weighted score

*Significant at p<0.0001

For peer review only

STROBE Statement—Checklist of items that should be included in reports of ***case-control studies***

	Item No	Recommendation	Page No
**Title and abstract**	1	(*a*) Indicate the study's design with a commonly used term in the title or the abstract	1, 3
		(*b*) Provide in the abstract an informative and balanced summary of what was done and what was found	3
**Introduction**			
Background/rationale	2	Explain the scientific background and rationale for the investigation being reported	5
Objectives	3	State specific objectives, including any prespecified hypotheses	5
**Methods**			
Study design	4	Present key elements of study design early in the paper	5,6
Setting	5	Describe the setting, locations, and relevant dates, including periods of recruitment, exposure, follow-up, and data collection	6, 7, 8
Participants	6	(*a*) Give the eligibility criteria, and the sources and methods of case ascertainment and control selection. Give the rationale for the choice of cases and controls	6-8
		(*b*) For matched studies, give matching criteria and the number of controls per case	6-8
Variables	7	Clearly define all outcomes, exposures, predictors, potential confounders, and effect modifiers. Give diagnostic criteria, if applicable	6-8
Data sources/ measurement	8*	For each variable of interest, give sources of data and details of methods of assessment (measurement). Describe comparability of assessment methods if there is more than one group	6-8
Bias	9	Describe any efforts to address potential sources of bias	6-8
Study size	10	Explain how the study size was arrived at	9
Quantitative variables	11	Explain how quantitative variables were handled in the analyses. If applicable, describe which groupings were chosen and why	7-8
Statistical methods	12	(*a*) Describe all statistical methods, including those used to control for confounding	8-9
		(*b*) Describe any methods used to examine subgroups and interactions	8-9
		(*c*) Explain how missing data were addressed	8-9
		(*d*) If applicable, explain how matching of cases and controls was addressed	8-9
		(*e*) Describe any sensitivity analyses	8-9
**Results**			
Participants	13*	(a) Report numbers of individuals at each stage of study—eg numbers potentially eligible, examined for eligibility, confirmed eligible, included in the study, completing follow-up, and analysed	9
		(b) Give reasons for non-participation at each stage	9
		(c) Consider use of a flow diagram	Figure 1
Descriptive data	14*	(a) Give characteristics of study participants (eg demographic, clinical, social) and information on exposures and potential confounders	9-10
		(b) Indicate number of participants with missing data for each variable of interest	10-11

For peer review only

Outcome data	15*	Report numbers in each exposure category, or summary measures of exposure	9-11

For peer review only

For peer review only - http://bmjopen.bmj.com/site/about/guidelines.xhtml

Main results	16	(*a*) Give unadjusted estimates and, if applicable, confounder-adjusted estimates and their precision (eg, 95% confidence interval). Make clear which confounders were adjusted for and why they were included	10-11
		(*b*) Report category boundaries when continuous variables were categorized	n/a
		(*c*) If relevant, consider translating estimates of relative risk into absolute risk for a meaningful time period	n/a
Other analyses	17	Report other analyses done—eg analyses of subgroups and interactions, and sensitivity analyses	11-12
**Discussion**			
Key results	18	Summarise key results with reference to study objectives	12
Limitations	19	Discuss limitations of the study, taking into account sources of potential bias or imprecision. Discuss both direction and magnitude of any potential bias	13-14
Interpretation	20	Give a cautious overall interpretation of results considering objectives, limitations, multiplicity of analyses, results from similar studies, and other relevant evidence	14-15
Generalisability	21	Discuss the generalisability (external validity) of the study results	14-15
**Other information**			
Funding	22	Give the source of funding and the role of the funders for the present study and, if applicable, for the original study on which the present article is based	16

*Give information separately for cases and controls.

Note: An Explanation and Elaboration article discusses each checklist item and gives methodological background and published examples of transparent reporting. The STROBE checklist is best used in conjunction with this article (freely available on the Web sites of PLoS Medicine at http://www.plosmedicine.org/, Annals of Internal Medicine at http://www.annals.org/, and Epidemiology at http://www.epidem.com/). Information on the STROBE Initiative is available at http://www.strobe-statement.org.

For peer review only
